# Influenza A H5N1 Immigration Is Filtered Out at Some International Borders

**DOI:** 10.1371/journal.pone.0001697

**Published:** 2008-02-27

**Authors:** Robert G. Wallace, Walter M. Fitch

**Affiliations:** Department of Ecology and Evolutionary Biology, University of California Irvine, Irvine, California, United States of America; University of Montreal, Canada

## Abstract

**Background:**

Geographic spread of highly pathogenic influenza A H5N1, the bird flu strain, appears a necessary condition for accelerating the evolution of a related human-to-human infection. As H5N1 spreads the virus diversifies in response to the variety of socioecological environments encountered, increasing the chance a human infection emerges. Genetic phylogenies have for the most part provided only qualitative evidence that localities differ in H5N1 diversity. For the first time H5N1 variation is quantified across geographic space.

**Methodology and Principal Findings:**

We constructed a statistical phylogeography of 481 H5N1 hemagglutinin genetic sequences from samples collected across 28 Eurasian and African localities through 2006. The MigraPhyla protocol showed southern China was a source of multiple H5N1 strains. Nested clade analysis indicated H5N1 was widely dispersed across southern China by both limited dispersal and long distance colonization. The UniFrac metric, a measure of shared phylogenetic history, grouped H5N1 from Indonesia, Japan, Thailand and Vietnam with those from southeastern Chinese provinces engaged in intensive international trade. Finally, H5N1's accumulative phylogenetic diversity was greatest in southern China and declined beyond. The gradient was interrupted by areas of greater and lesser phylogenetic dispersion, indicating H5N1 migration was restricted at some geopolitical borders. Thailand and Vietnam, just south of China, showed significant phylogenetic clustering, suggesting newly invasive H5N1 strains have been repeatedly filtered out at their northern borders even as both countries suffered recurring outbreaks of endemic strains. In contrast, Japan, while successful in controlling outbreaks, has been subjected to multiple introductions of the virus.

**Conclusions:**

The analysis demonstrates phylogenies can provide local health officials with more than hypotheses about relatedness. Pathogen dispersal, the functional relationships among disease ecologies across localities, and the efficacy of control efforts can also be inferred, all from viral genetic sequences alone.

## Introduction

Highly pathogenic influenza A H5N1 has killed millions of birds from a variety of taxonomic orders. Since 2003, an additional 353 human infections have been confirmed with a mortality rate over 60% (WHO, 24 January 2008). The infection's malignant pathogenesis [1] and what appear to be lengthening, though still limited, chains of human transmission [2], [3] have placed the world on high alert. H5N1 appears to be the leading candidate among several influenza subtypes to evolve a human-to-human infection that sets off a worldwide pandemic.

Since early 2005 avian H5N1 has migrated out of East Asia across Eurasia and as far west as England and West Africa [4]. There are apparent dangers in the extent of such geographic spread above and beyond starting new outbreaks. First, H5N1 is finding the regions of the world where animal health monitoring remains underdeveloped or degraded by national structural adjustment programs associated with international loans [5], [6]. Unchecked transmission in these areas increases the genetic variation with which H5N1 can evolve human-specific phenotypes. In spreading over three continents fast-evolving H5N1 also contacts an increasing variety of socioecological environments, including locale-specific combinations of prevalent host types, modes of poultry farming, and animal health measures.

Geographic variation in H5N1 spread, then, appears of essential importance to public health. Whether some localities have been subjected to greater H5N1 immigration is one of several pertinent questions that remain little-addressed. Are some localities more resistant to introgression? Do some countries host greater H5N1 diversity? Circumstantial evidence, derived largely by visually inspecting H5N1 phylogenies and by the relative frequencies of reassortant genotypes, has suggested southern China is the source of, and plays host to, multiple H5N1 strains [7], [8]. We present here the first attempt to quantify such diversity across geographic space using a statistical phylogeography of 481 hemagglutinin nucleotide sequences from H5N1 samples isolated 1996–2006 in 28 Eurasian and African localities (Table S1). Specifically, we test the hypothesis H5N1 diversity declines from southern China and that some localities host greater diversity than expected along the spatial cline.

## Results

### Influenza A H5N1 Migration

As a first step we tracked the migration of highly pathogenic H5N1 during its evolutionary history. A phylogenetic tree of the 481 hemagglutinin sequences was constructed via gamma-corrected, general time-reversible maximum likelihood (Figure S1). The migration events through the tree were traced with MigraPhyla [9], [10]. The method infers the localities of the tree nodes by maximum parsimony, and by Monte Carlo simulation tests the significance of migration events that accrue back and forth between all pairs of localities over time.

**Figure 1:**
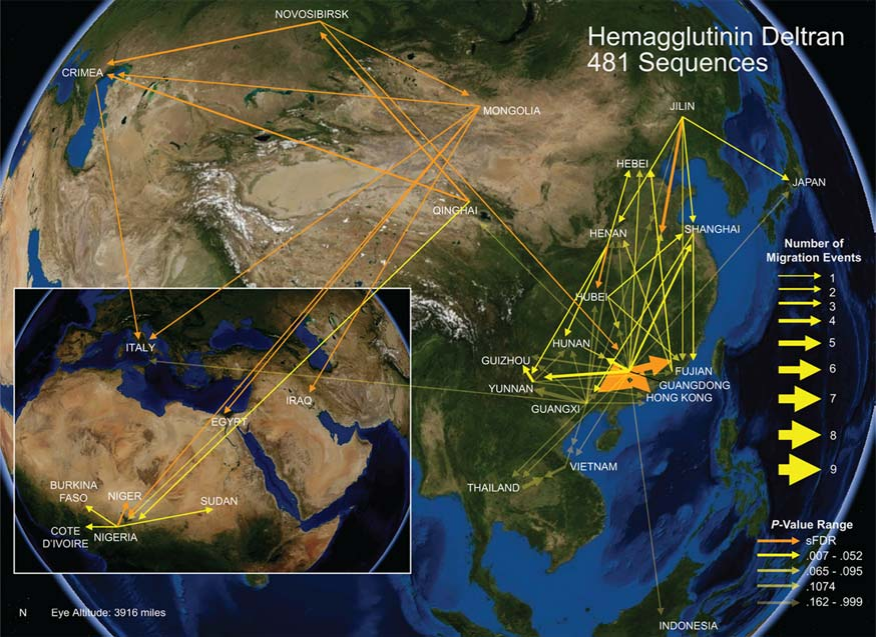
H5N1 Migration as Inferred by Parsimony. Map of H5N1 migration events inferred by parsimony through maximum likelihood phylogeny for 481 hemagglutinin nucleotide sequences sampled across 28 Eurasian and African localities 1996–2006 (2≤*n*≤65 isolates per locality). Orange vectors are statistically significant (α = .05) under an upper-tail Monte Carlo test of 10,000 trials and a sparse false discovery rate (sFDR) correction. Non-significant vectors are color-coded by Monte Carlo *P* value: the brighter the yellow, the greater the support. Quintiles are defined by breaks in ranked *P* values of more than .01, except within the final quintile. The map is based on satellite photos made available in World Wind 1.4 (http://worldwind.arc.nasa.gov/).

MigraPhyla shows that the Chinese province of Guangdong was a primary source for multiple regional and international H5N1 migration events, including for all four regional clades: 1, 2.1, 2.2, and 2.3 (Figure 1) [7], [9]. With only about half the number of viruses sequenced as Guangxi, Thailand, and Indonesia, in this analysis Guangdong accounted for 41 of the 115 (36%) total migration events inferred (Figure S2). The results may be an artifact of the method of inference, which searches for the most parsimonious distribution of ancestral localities through the original genetic tree, often favoring dispersal from a single or limited number of epicenters [9]. However, Guangdong's widespread representation across multiple tree clades for both hemagglutinin and neuraminidase is unmistakable (Figures S3 and S4), imparting the province an integral role in H5N1's epidemiology. Even if H5N1 strains emerged elsewhere in the region instead, Guangdong's socioeconomic centrality may have acted as an epidemiological attractant, drawing in novel poultry trade-borne strains from around southern China before dispersing them again back out across China and beyond [11].

The phylogeography of H5N1 involves much more than spread from a primary epicenter. Regional dynamics are apparent, including among Chinese provinces and in Africa (Figure 1). Secondary epicenters, such as Qinghai and Nigeria, seeded tertiary outbreaks, including, in the case of Novosibirsk, back east into China [12]. The origins of other outbreaks, on the other hand, appear more straight-forward. In spite of a ten-fold increase in the number of samples from our previous analysis, Indonesia's epidemic still appears founded by a single migration event from southern China [9], [13]. A few viruses from a single batch of infected poultry could very well have acted as the progenitors of outbreaks now stretching across 31 of Indonesia's 33 provinces.

**Figure 2:**
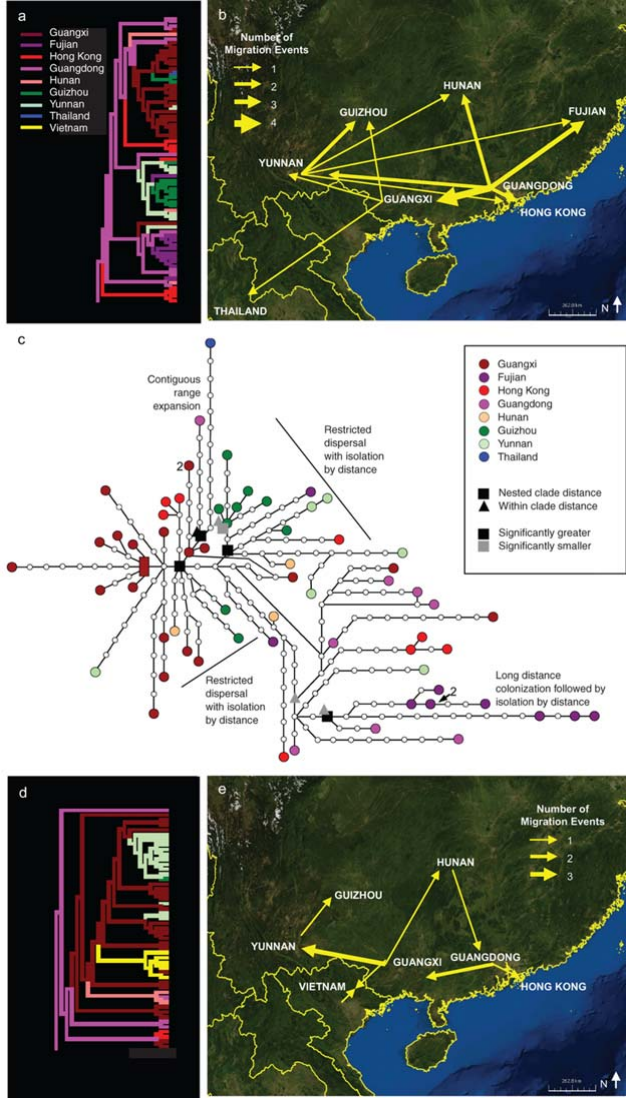
H5N1 Migration in Southern China. a) H5N1 migration events as assigned by parsimony for 67 samples of a single clade 2.3 H5N1 strain circulating in southern China 2005–2006 and b) mapped onto a World Wind satellite photo. c) Statistical parsimony haplotype network for same 67 samples. Rectangle represents the hypothesized ancestral genotype. All genotypes, except two, are represented by a single isolate. The exceptions are represented by two isolates (‘2’). Squares represent significant nested clade distances and triangles significant within clade distances as determined by nested clade analysis. Black shapes represent nodes at which subclades express significantly greater distances than expected by chance begin and extend to the tips of the branch. Gray shapes represent the starting node of a subclade of lesser distance than expected by chance. d) Migration events through section of H5N1 maximum likelihood tree showing 49 samples of a single clade 2.3 H5N1 strain circulating in southern China 2004–2006 and sister group to the 67 isolates shown in (a). e) The migration events for the 49 isolates mapped onto a World Wind satellite photo.

As patterns of migration and trade within countries can qualitatively differ from those among countries [14], we next explored H5N1 spread within China. A section of the original hemagglutinin maximum likelihood tree was used to map the migration events for 67 samples of a single clade 2.3 H5N1 strain circulating in southern China 2005–2006 (Table S1, Figure 2a,b). Over the course of a year, the strain traversed much of southern China and moved into Thailand [15]. The result was reproduced by nested clade analysis [16], [17], another phylogeographic method (Figure 2c). For 12 of 16 nested clades with geographic variation across H5N1 genotypes, nested clade analysis's permutation tests failed to reject the null hypothesis that H5N1 was randomly distributed across southern China. Four of the five subclades whose nested clade distances were significantly different from randomness proved more widely dispersed from their sister subclades than expected by chance. Templeton's [17], [18] inference key showed the significant clades to be defined alternatively by restricted dispersal with genetic isolation by distance and long-distance colonization and expansion. A closely related co-circulating strain, as represented by 49 clade 2.3 samples isolated 2004–2006, engaged in similarly extensive dispersal (Figure 2d,e).

### Evolutionary History Shared Across Localities

In tracing migration events through a genetic tree we can learn much about H5N1's dispersal patterns, including their extent and magnitude. However, there remains little understanding of the functional relationships among the localities' disease ecologies. To clarify some of the mechanisms involved in H5N1 spread we conducted two additional analyses. In the first, we determined which Eurasian and African localities hosted similar H5N1 evolutionary histories by conducting a principal components analysis on the UniFrac metric for the full hemagglutinin phylogenetic tree. UniFrac measures the fraction of the phylogenetic distance leading to samples of either one of two localities, but not to both [19]. It measures the branch length unique to each locality. Conversely, the pair of localities with the smallest UniFrac distance shares the greatest amount of evolutionary history.

**Figure 3:**
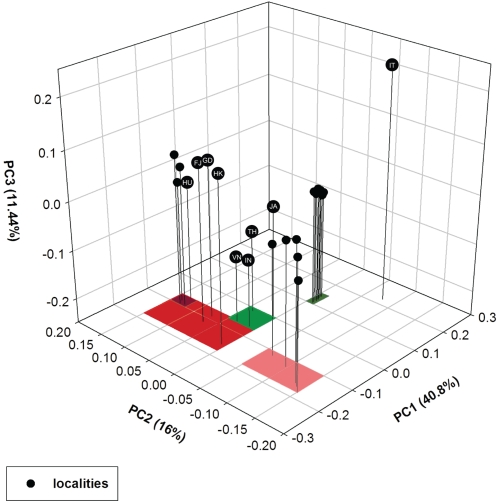
UniFrac PCA of H5N1 Shared History by Locality. First three principal components for PCA of UniFrac metric for 481 H5N1 hemagglutinin sequences across 28 localities. Localities in red are situated in southern China, in dark red southwestern China (Hunan, Guizhou, Yunnan, and Guangxi), in pink northern China, in green in nearby Asian countries (Indonesia, Japan, Thailand, and Vietnam), and in olive green regions reached by the Qinghai-like strain (e.g., Mongolia, Crimea, Iraq, Africa). Localities referred to in the text include FJ = Fujian, GD = Guangdong, HK = Hong Kong, HU = Hunan, IN = Indonesia, IT = Italy, JA = Japan, TH = Thailand, VN = Vietnam.

The first three principal components accounted for 68% of the total variation in shared phylogenetic history across localities (Figure 3). PC1 separated eastern Chinese localities from several nearby Asian countries and, farther west, areas infected by the Qinghai-like H5N1 strain. In effect, PC1 reproduces the major patterns depicted in the MigraPhyla map (Figure 1), with nearby localities sharing H5N1 evolutionary history. We learn in addition that China's H5N1 strains group together north and south to the exclusion of those outside the country, with one exception. Hunan, a southern province, grouped with the other Asian countries: Indonesia, Japan, Thailand, and Vietnam. As measured here Hunan appears peripheral to a phylogeographic structure arrayed over northern and southern China.

On the other hand, PC2 divided northern from southern China, implying that however integrated the two H5N1 socioecologies may be, each region also hosts its own localized diffusion. The other Asian countries nearby clustered together along this second axis with southern China, in particular with Guangdong, Fujian, and Hong Kong, the southern face of China's international trade, indicating a shared phylogeography. Chinese provinces farther west (Hunan, Guizhou, Yunnan, and Guangxi), and historically less integrated with trade [20], clustered on their own. Finally, localities infected by the Qinghai-like strain tightly clustered in the PCA space. In a matter of months, across three continents, the Qinghai-like strain successfully invaded a large section of what was, until 2005, H5N1-free territory [4].

### Phylogenetic Structure Across Localities

**Figure 4:**
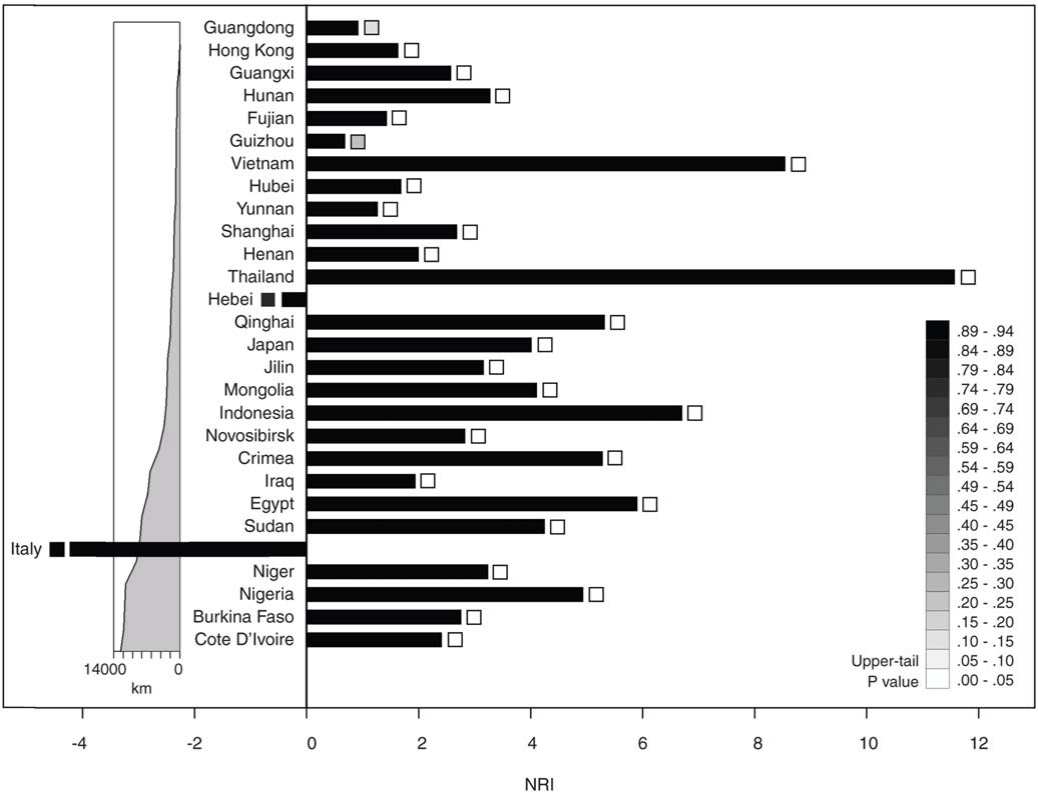
Phylogenetic Dispersion Across H5N1 Localities. Net relatedness index (NRI) across 28 localities listed by great circle distance (*km*) from Guangdong. The upper-tail *P* values for the Monte Carlo test for phylogenetic clustering are colorized along a grayscale and shown in boxes at the tips of their respective index bars.

We next tested the extent to which the H5N1 isolates were distributed across the phylogenetic tree. Some localities may host samples from widely disparate phylogenetic clades, while other localities may host samples from a single clade alone. Two measures of phylogenetic dispersion, the net relatedness index (NRI) and the nearest taxon index (NTI), were rank-ordered by great circle distance from Guangdong, a putative primary epicenter for H5N1 (Figures 4 and S5). The indices represent standardized effect sizes of community phylogenetic structure [21]. NRI is defined for the mean phylogenetic distance (MPD) among a locality's samples, measuring dispersion over the whole tree. NTI is defined for the mean phylogenetic distance between nearest tree neighbors (MNND), measuring the extent to which locality samples are clustered at the tree's tips.

The great majority of localities in the study were defined by statistically significant phylogenetic clustering associated with local evolutionary radiation. However, some localities were less clustered than others. The NRI results show localities within China listed down through Hebei in Figure 3, except Hunan, tended to be less phylogenetically clustered than localities below Hebei. NRI for two of these localities, Guangdong (NRI = 0.9141) and Guizhou (0.6794), were statistically indistinguishable from random dispersion. The lesser clustering for areas nearer Guangdong agrees with previous work indicating southern Chinese provinces have hosted multiple H5N1 strains [6].

On the other hand, Vietnam (8.5366) and Thailand (11.5646), although relatively close to Guangdong, showed extreme phylogenetic clustering (*P* = 0). The result suggests an as yet unknown mechanism has repeatedly filtered out H5N1 as the virus moved from southern China towards or into Indochina. Whether the filter is embodied by customs regulation, limited transportation infrastructure, or the vagaries of the poultry market remains an open question. The filtering occurred even as repeated outbreaks accrued within Indochina, among regional localities [9], and a limited number of migration events were recorded between the two areas.

Other borders appear less resistant to invasion when exposed. Japan (NRI = 4.0085) hosted widely differing H5N1 strains that originated in northern and southern China (Figure 1), by way of South Korea and China direct [22], [23], and, in late 2006, the Qinghai-like strain. Nigeria (4.9317) has hosted multiple introductions of the strain [24]. Nigeria's greater NRI score may have resulted in part from how quickly introgressions of related haplotypes followed each other into the country. Ducatez *et al*. [25] report near-simultaneous outbreaks in Nigeria were caused by different Qinghai-like sublineages.

At the other extreme, Italy's (NRI = −4.2206) H5N1 samples were significantly evenly distributed across the phylogeny. Italy hosted two Qinghai-like strains and what appears to be an older haplotype (A/mallard/Italy/3401/2005(H5N1)) from Guangxi direct (Figure 1). The Guangxi connection, however, is likely an artifact of long-branch attraction in the original gene tree (Figure S1). Chinese province Hebei (−0.4348) is nearly, though not significantly, evenly distributed. The three lone Hebei isolates included in the study were derived from different strains and localities (Figure 1) [9]. Multiple introductions from several sources can provide a population sink greater genetic variation than expressed by any single native source [26].

### Spatial Correlation and Sampling Effort

Our analyses of UniFrac and the indices of phylogenetic dispersion did not directly test for spatial correlation. To determine how the phylogenetic measures reviewed here were related to each other, to spatial distance, and to sample size, we conducted two convergence tests and a pairwise Mantel test for correspondence [27] for six distance matrices defined for the H5N1 hemagglutinin phylogeny. The six matrices were 1) MigraPhyla migration events through the phylogeny symmetricized across the migration matrix diagonal, showing average two-way traffic between localities; 2) differences in isolate sample size; 3) geographic distances; 4) UniFrac distances; 5) differences in MPD; and 6) differences in MNND.

**Figure 5:**
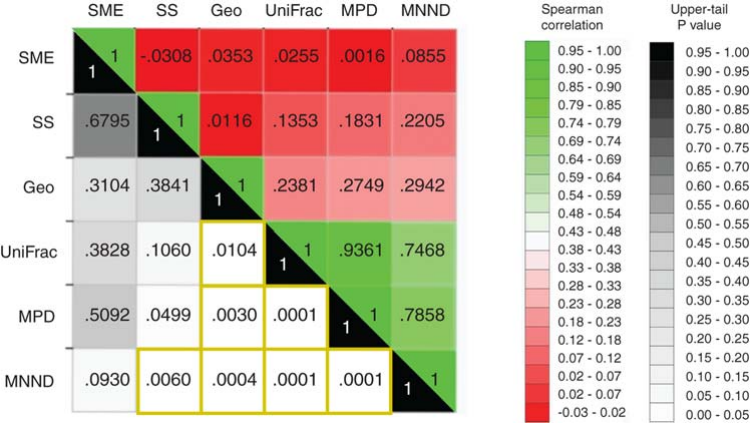
Generalized Mantel Test Across H5N1 Distance Matrices. Pairwise Mantel test for correspondence based on Spearman correlation ranks for six distance matrices defined for H5N1 phylogeny of 481 hemagglutinin nucleotide sequences across 28 Eurasian and African localities: 1) migration events through the phylogeny symmetricized across the migration matrix diagonal (SME), 2) differences in isolates sample size (SS), 3) geographic distances (Geo), 4) UniFrac distances, 5) mean phylogenetic distances (MPD), and 6) mean nearest neighbor distances (MNND). Correlations are blocked out in color and upper-tail *P* values in gray scale. *P* values for pairwise comparisons between localities significant under false discovery rate correction are boxed in gold borders.

The test for global convergence across distance matrices (CADM) rejected overall incongruence (*P* = .0001)—at least two of the matrices converged (Table S2). The *a posteriori* convergence tests we next conducted failed to reject the null hypothesis that the matrix of symmetricized migration events (*P* = .30) was incongruent with the other five matrices. The generalized Mantel test, based on Spearman correlation of ranks and a false discovery rate correction, identified seven pairwise comparisons as significantly convergent (Figure 5). In rank order of the strength of correlation, the significant pairs were MPD-UniFrac (*r* = .93), MPD-MNND (.78), MNND-UniFrac (.74), geographic distance-MNND (.29), geographic distance-MPD (.27), geographic distance-UniFrac (.23), and sample size difference-MNND (.22).

The Mantel tests showed that the shared evolutionary history and phylogenetic dispersion for these H5N1 localities were, as of 2006, tightly associated. Indeed, the third principal component in the UniFrac PCA (Figure 2), which we left unexplained above, strongly correlated with NRI (*r* = .66). A second result supported what the NRI results alone suggested: phylogenetic dispersion appears spatially autocorrelated, with important exceptions (*e.g.*, Italy, Iraq). Finally, MNND was dependent on sample size and significantly varied with sampling effort. MPD, however, did not, implying that the conclusion southern China hosted greater overall H5N1 diversity is statistically unrelated to differential sampling across space.

## Discussion

Our MigraPhyla analysis through 2006 agreed with previous work showing several regional and international strains of highly pathogenic influenza A H5N1 originated in southern China (Figure 1). Spatially finer analysis indicated novel outbreaks widely dispersed across southern China over the course of a year, by both limited dispersal and longer strikes (Figure 2). Oyana *et al.* [28] showed the time of dispersal through China following an initial outbreak may be compressed several orders of magnitude to a matter of weeks. The exact mechanism for H5N1 spread through China remains only loosely documented and requires additional attention. Migratory birds, the poultry trade, and the transport of free-range ducks have all been implicated in outbreaks across Southeast Asia [7], [29], [30].

A principal components analysis of the UniFrac metric identified combinations of localities that shared the greatest amount of H5N1 evolutionary history (Figure 3). UniFrac has so far been largely applied to determining the effects of environment type on microbial evolution, particularly for bacteria. This appears the first time the metric has been used in a strictly phylogeographic context. The method offers a means by which to identify groups of localities, at a variety of spatial scales, whose isolates evolve together out from a local common ancestor.

The PCA showed variation in H5N1's shared history segregated along orthogonal axes defined by separate phylogeographic processes. First, localities in China grouped to the exclusion of nearby Asian countries, the rest of Eurasia, and Africa. Along the second axis northern and southern China separated. In spite of clear epidemiological interaction between the two Chinese regions, each also hosted its own localized diffusion. Indonesia, Japan, Thailand and Vietnam also grouped with southern China, particularly with southeastern provinces engaged in intensive international trade. The latter result suggests H5N1 migration among these areas is largely structured by poultry shipments, even as migratory birds may also seed local outbreaks (*e.g.,* Lake Qinghai) [7], [31], [32].

The indices of dispersion reported here, another phylogenetic measure, show H5N1's accumulative diversity drops off from southern China, the putative region of origin. The step gradient is punctuated by areas of greater and lesser phylogenetic spread. These interruptions might arise from a variation in epidemiological permeability across provincial and international borders. Some localities, Thailand and Vietnam for example, appear more resistant to H5N1 introduction than others. Identifying these localities—and their relevant borders—may aid efforts at learning what socioecological factors block (or enable) H5N1 migration at a variety of spatial scales. The methods presented here may also provide some of the metrics needed to define criteria for bird flu mitigation. For instance, localities with diminishing increases in accumulative phylogenetic dispersion may increasingly filter out H5N1 immigration.

H5N1 UniFrac and phylogenetic dispersion appeared strongly correlated. We propose that localities in regions newly invaded by a single strain share both viral phylogenetic history and, by definition, limited dispersion (no other strains have yet invaded). The most obvious example is that of the Qinghai-like strain that dominates Europe and Africa. Similarly, local areas subjected to repeated invasions from a nearby source share many related strains before subsequent differentiation by isolation by distance accrues with additional geographic spread. Localities abutting sources, then, share both phylogenetic history and a comparatively evener dispersion. There are important exceptions for H5H1, as Thailand and Vietnam demonstrate.

This appears the first time measures of phylogenetic community structure have been derived from the genetic sequences of a single spreading pathogen. The indices of phylogenetic dispersion have typically been applied at the community level (*e.g.*, among plant species in a neotropical forest) [33]. The H5N1 results did agree with what are emerging as general principles in studies of community assembly. We discuss two here. By simulation analysis Kraft *et al.* [34] show the process of habitat filtering that produces phylogenetic clustering is best detected by the net relatedness index than by the nearest taxon index, as was the case here for H5N1. The assembly rules for H5N1 diversity, however, may arise as much from biogeographic processes limiting dispersal as from socioecological factors filtering out emerging strains [35].

The balance between the two processes may shift back and forth. A H5N1 strain that spreads out to a broader regional scale temporally diminishes the effects of isolation by distance, including on measures of phylogenetic dispersion. Since its emergence highly pathogenic H5N1 has undergone a series of demic selection events wherein a strain emerges to outcompete indigent strains across a wide spatial extent. The Z reassortant outcompeted at least five other strains to emerge as the dominant genotype in East Asia [36]. The Qinghai-like strain has dispersed across Eurasia and into Africa [4]. The Fujian-like strain is the most recent to be detected [8]. For areas already hosting endemic bird flu, a newly dispersed strain will increase phylogenetic evenness. For areas never before invaded, an expanding strain by definition causes significant phylogenetic clustering, in this case, accompanied by a shared phylogenetic history (see localities infected by the Qinghai-like strain in Figure 3). Areas that evade invasion by a widely dispersed strain altogether may do so by chance, but may instead do so by virtue of a socioecological filter blocking entry.

In spreading, H5N1 changes the relationship between phylogenetic dispersion and shared history across localities. A spreading pathogen also changes the combination of interventions necessary for its control [37]. Intervention strategies that may have worked when H5N1 outbreaks were confined to Southeast Asia [38] are for the most part rendered obsolete. A stockpile of oseltamivir or vaccines amassed for blockading any potential outbreak of human-specific strains in East Asia becomes of dubious utility with bird flu spread across—and evolving in—Eurasia and Africa. Intervention strategies must undergo qualitative changes when the epidemic's increasing spatial extent changes the mix of locally endemic strains.

Kraft *et al.* also show habitat filtering generates stronger phylogenetic structure when novel derived traits are favored, as appears the case in influenza. Strains of seasonal influenza A H3N2 with the greatest change at 18 HA1 codons serve as progenitors for the next year's dominant strain [39]. Debate continues over whether the positive selection to which antigenic sites are subjected is continual or punctuated [40]–[42]. Other work suggests influenza evolution results from punctuated changes in antigenic properties rather than genetic change alone [43]. Yet another model proposes these antigenic changes arise from shifts in host population immunity [44]. By whatever mechanism antigenic drift emerges, an accumulation of derived traits driven by host immune response appears favored.

Influenza's accelerated evolution, however, is complicated by multiple reversals and parallelisms, with specific amino acid residues evolving time and again [40], [45]. Parallelisms and correlated evolution among residue sites may allow H5N1 to infect cycles of host species repeatedly encountered across geographic space [9]. The homoplasies may complicate phylogenetic reconstruction, but the capacity for parallel changes can be considered a character itself, a notion that harkens back to E. Ray Lankester's original intent in defining ‘homoplasy’ [46]. Homoplasies are a real feature of influenza's biology and can be thought of as a type of homology, not its antonym. Indeed, for certain influenzas, parallel changes may serve as a predictor of the emergence of a new dominant strain [40]. The relationships among influenza's molecular evolution, phylogenetic structure, and geographic spread remain an active field of research.

The methodologies described here may benefit studies of other animal and human pathogens. The phylogeographic dynamics of a variety of newly emergent threats such as HIV, SARS, and hantavirus, as well as those of deeper historical origin, such as HTLV and KSHV, may be better illuminated by the approach. For many of these pathogens the key problem for control efforts may not be in determining whether these phylogenetic variables are spatially autocorrelated, but in explaining their residuals, what spatial distance does not explain [47]. Assuming pathogen spread is in part deterministic, arising from specific causes rather than by chance alone, localities with greater and lesser dispersion than expected may be more exposed to, or protected from, new strains of the disease under investigation. In using genetic sequences to learn which of such outcomes prevail where, health officials may be able to better track the finer patterns in pathogen spread.

## Materials and Methods

### Samples

From GenBank [48] and the Influenza Sequence Database [49] we downloaded 481 influenza A H5N1 hemagglutinin and 429 neuraminidase nucleotide sequences isolated from a variety of host species 1996–2006 across 28 Eurasian and African localities (Table S1). The hemagglutinin sequences used in this study ranged over 1716 positions, indels included, covering all of the precursor peptide, HA1 and HA2, except HA2's final six nucleotide sites. The neuraminidase sequences sampled for this study covered the entire monomer, across 1419 sites, indels included. Neuraminidase sequences were downloaded only for hosts that also contributed hemagglutinin sequences to the study.

Our analyses used a convenience sample of sequences made publicly available by a variety of researchers from around the world, sampling at a variety of geographic scales. The localities sampled were represented by a variable number of isolates (*n* = 2 to 65) and included the Chinese city of Shanghai; the Hong Kong Special Administrative Region of China; the Chinese provinces of Hebei, Henan, Hubei, Hunan, Guangdong, Guangxi, Guizhou, Fujian, Jilin, Qinghai, and Yunnan; the countries of Burkina Faso, Cote d'Ivoire, Egypt, Indonesia, Iraq, Italy, Japan, Mongolia, Niger, Nigeria, Sudan, Thailand, and Vietnam; the Russian city of Novosibirsk and its environs; and the Crimean region. Duplicate sequences sampled from the same locality add no phylogeographic information to the analysis and were not included among the 481 hemagglutinin samples used. On the other hand, duplicate sequences from different localities were included, as they are by definition differentiated by a migration event.

### Phylogeny

The nucleotide sequences were aligned in ClustalW [50]. Leading and trailing gaps from truncated sequencing were treated as missing data. Gaps within the sequences (*e.g.*, of a length of 19–20 residues in the neuraminidase stalk) were treated as real indels and defined as a fifth character state. Tree-building was a two-step process: A preliminary input tree was first constructed by maximum parsimony in PAUP* 4.0b10 [51]. Character state changes were ordered by outgroup comparison with isolate A/chicken/Scotland/59(H5N1). Most-parsimonious trees were heuristically generated by stepwise addition and branch swapping via tree-bisection-recombination. One tree was chosen by visual inspection as representative of the set of 1000 and used as the preliminary input tree for the construction of a maximum likelihood tree. The maximum likelihood tree was created via a gamma-corrected, general time-reversible model in GARLI [52]. Bootstrap values were derived in PAUP by a fast-heuristic search of 1000 replicates.

### Statistical Phylogeography

As in Slatkin and Maddison [53], the localities of the isolates in the resulting gene tree were assigned to the tips as a single character with 28 states. Moving recursively up the tree, PAUP* 4.0 assigned the localities of ancestral nodes by maximum parsimony, such that the tree supported the fewest possible migration events between localities consistent with the gene tree. PAUP's assignments may represent only one of several possible most-parsimonious traces. For the hemagglutinin tree several nodes support multiple localities (Figure S3) [54].

For each gene, the total number of migration events occurring through the tree as assigned by PAUP was tallied for every possible pair of localities using the MigraPhyla protocol [9], [10]. A Monte Carlo test of 10,000 trials was conducted to determine the probability that the frequencies of migration events between each pair of localities in the original migration tree were more than expected when the localities were randomly distributed across the tree's tips. The localities of the real data sets were randomized without changing the sample sizes for each locality. The migration events among localities through the gene tree were inferred by DELTRAN optimization and the migration events among localities summed for each randomized trial. Under DELTRAN, character state changes in the phylogeny—here, migration events—are delayed and concentrated toward the tips of the tree. To test the significance of the resulting *P* values across all localities, α, the nominal Type 1 error rate, was controlled for multiple tests across locality pairs with a sparse false discovery rate correction [9]. The series of programs for running the MigraPhyla protocol are currently available at http://pd.bio.uci.edu/ee/WallaceR/MigraPhyla.html.

To determine the degree to which the hemagglutinin and neuraminidase differed in migration structure we reran the DELTRAN migration analysis on the 429 H5N1 isolates for which hemagglutinin and neuraminidase sequences (HA429 and NA429) were both available (Table S1 and Figure S4). All localities from the HA481 analysis were included, except Burkina Faso, for which no neuraminidase sequences were available at the time of analysis. As a first effort at exploring the effects of sampling schema on our migration estimates, we also included the HA481 migration matrix without migration events to and from Burkina Faso. To compare the three matrices' structure we conducted a singular value decomposition of each matrix and calculated the angles at which each resulting eigenvector differed between the genes [9]. Specifically, we calculated the arccos of the scalar product of the resulting eigenvectors, divided by the product of their norms, contrasting the three migration matrices in round robin fashion. Many of the eigenvectors differed orthogonally (Figure S6). HA429 and HA481 showed the least convergence, indicating changing sampling effort across localities can change the resulting migration structure. A fuller sensitivity analysis exploring the effects of sampling on migration estimates is planned.

### Methodological Equivalence

In an effort at testing for methodological equivalence [55], we compared a map of migration events derived by MigraPhyla for 67 Chinese clade 2.3 hemagglutinin sequences isolated 2005–2006 to the results of a nested clade analysis (NCA) [16], [17], [56], [57] of the same isolates.

For the MigraPhyla analysis, the node localities—and their associated migration events—were retained from the original tree of 481 sequences. For the NCA analysis a gene network of the 67 isolates was first constructed via statistical parsimony in TCS 1.21 [58]. Traditional phylogenies can fail to account for many of the inherent properties of intraspecific genealogies, such as extant ancestral taxa, multifurcated tree branching, and true homoplasies brought about by recombination. These properties can be assimilated by a parsimonious network of isolates with statistically supported connections [59]–[61]. The best network expresses a high probability (≥95%) its linkages—regardless of the number of nucleotide steps between isolates—are true. In short, the network assimilates statistically supported deviations from parsimony. TCS also calculates the root probabilities for each isolate using Castelloe and Templeton's [62] heuristic.

Loops in the network represent homoplasies, including possible recombination events. The method hypothesized isolate A/chicken/Guangxi/463/2006(H5N1), with the greatest root probability, to be the ancestral haplotype, almost certainly incorrect with many isolates sampled in 2005 also included here. As expected for an RNA pathogen with a high mutation rate and a low sample-to-population ratio, the resulting network also contained many more missing haplotypes than such networks typically represent. Viral haplotype networks have for the most part been restricted to characterizing within-patient evolution, the diversity from which is comparatively restricted [63].

In spite of these complications, the geographic associations among the haplotypes were tested via NCA. To statistically test for associations between the parsimonious network and geography the branches are first organized in a nested fashion by single steps in the network. The nesting begins at the tip isolates and includes those internal taxa one step away—the 0-step—from the terminal. These are subsequently nested into a nest of 1-step clades, then 2-step clades, and so on, until the next nest would include all the isolates inside. Two distances are now constructed: the clade distance, measuring the geographic spread of a clade (say, within a 1-step nest); and the nested clade distance, measuring the geographic distances among clades within the same nested category (among the 1-step nest clades within their 2-step nest).

With GeoDis 2.5 [18] the statistical significance of the two distances across the clade nests was determined by contrasting them with nonparametric distributions of distances generated by Monte Carlo randomizations of the data, under the constraint haplotype frequencies and sample sizes are preserved [57]. If patterns are significant they require biological interpretation. Templeton [17] provides a key (updated on the GeoDis website) with which the distribution of significant results across the nests can be used to discern which evolutionary process likely caused the results at each nest level. The factors potentially influencing haplotype distribution include ongoing restricted gene flow or dispersal, historical range expansion, ancestral allopatric fragmentation, hybridization, and inadequate sampling.

### Shared Phylogenetic History and Dispersion

We determined which localities shared similar H5N1 evolutionary histories by conducting a principal components analysis on the UniFrac metric for the phylogenetic tree. UniFrac measures the phylogenetic distance between the samples of two localities in terms of the branch length in the tree that is unique to each locality [19], [64]. The pair of localities with the smallest UniFrac metric shares the greatest amount of evolutionary history. UniFrac was weighted here by the relative abundances across localities and standardized by the different rates of evolution across branches.

We next tested for geographic structure in the extent to which H5N1 isolates were distributed across the phylogenetic tree. Two measures of phylogenetic dispersion, the net relatedness index (NRI) and the nearest taxon index (NTI), represent standardized effect sizes of community phylogenetic structure [21], [65]. They define for each locality the differences between the average phylogenetic distance among samples for the observed data and their analogues for randomized null data, scaled by the standard deviation of the null data. The phylogenetic distances were derived from the original maximum likelihood tree. NRI is defined for the mean phylogenetic distance (MPD) among a locality's samples, measuring dispersion over the whole tree. NTI is defined for the mean phylogenetic distance between nearest tree neighbors (MNND), measuring the extent to which locality samples are clustered at the tree's tips.

The significance of the dispersion indices was determined in Phylocom 3.41 [66] via one-tail tests against a null model of 999 randomized trials and 50 independent swaps. For each independent swap, the cells of submatrices in the locality matrix defined by presence/absence “checkerboards” are swapped [67], [68]. The marginals of the locality matrix for each swap are constrained to those of the original matrix, preserving the localities' sample frequencies.

To determine whether the phylogenetic measures reviewed here were related to each other or to spatial distance or sample size, we conducted global and *a posteriori* convergence tests and a generalized pairwise Mantel test for correspondence in CADM [27], [69] for six distance matrices defined for the H5N1 hemagglutinin phylogeny. The six matrices were 1) MigraPhyla migration events through the phylogeny symmetricized across the migration matrix diagonal (SME), showing average two-way traffic between localities; 2) differences in isolate sample size (SS); 3) geographic distances (Geo); 4) UniFrac distances; 5) MPD distances; and 6) MNND distances.

The CADM method, based on Friedman's chi-square and Kendall's coefficient of concordance *W*, tests the null of overall incongruence across all inputted distance matrices [27]. The significance of the chi-square is determined by way of a one-tail Mantel-like permutation test of, in this case, 9999 trials, permuting the labels of the matrices' rows and columns (and not the distances themselves or their ranks). If the null is rejected, the *a posteriori* tests can be used to identify the incongruent matrices. Unlike the global test, in which all the matrices in the analysis are permutated, only a single distance matrix is permutated at a time for the *a posteriori* tests. Finally, Mantel tests can be conducted to identify pairs of congruent matrices. The tests are based on Spearman's correlation coefficient and conducted in round robin fashion across the matrices.

## Supporting Information

Table S1 Study H5N1 hemagglutinin (HA) and neuraminidase (NA) sequences by sample locality.
(0.08 MB PDF)

Table S2 Global and a posteriori convergence across distance matrices (CADM) tests for six distance matrices defined by the H5N1 hemagglutinin phylogeny.
(0.02 MB DOC)

Figure S1 a) Maximum likelihood radial phylogeny for 481 sequences of H5N1 hemagglutinin (HA) sampled across 28 localities in Eurasia and Africa. Tree is rooted by isolate A/chicken/Scotland/59. b) 50% majority rule consensus tree with bootstrap values for 1000 replicates.
(0.16 MB PDF)

Figure S2 Migration events defined via DELTRAN parsimony through H5N1 maximum likelihood phylogeny of 481 hemagglutinin sequences sampled across 28 localities in Eurasia and Africa.
(0.25 MB PDF)

Figure S3 H5N1 migration events assigned on H5N1 hemagglutinin tree by DELTRAN parsimony. Change in color represents migration event. PAUP assignments may represent only one of several possible most-parsimonious traces. Mesquite trace here shows several nodes support multiple localities. Branches that support multiple localities are striped with associated colors. For the best view of isolate names and branch colors, zoom in to at least 800% in Adobe Acrobat.
(0.08 MB PDF)

Figure S4 Map of DELTRAN H5N1 migration events inferred through maximum-likelihood phylogeny for 429 hemagglutinin (a) and neuraminidase (b) nucleotide sequences sampled across 28 Eurasian and African localities 1996–2006 (1≤n≤64 isolates per locality). Orange vectors are statistically significant (α = .05) under an upper-tail Monte Carlo test of 10,000 trials and a sparse false discovery rate (sFDR) correction. Non-significant vectors are color-coded by Monte Carlo *P* value: the brighter the yellow, the greater the support. Quintiles are defined by breaks in ranked *P* values of more than .01, except within the final quintile. The map is based on satellite photos made available in World Wind 1.4 (http://worldwind.arc.nasa.gov/).
(0.54 MB PDF)

Figure S5 Net relatedness index (NRI), nearest taxon index (NTI), and associated upper-tail *P* values across 28 localities listed by great circle distance from Guangdong. The original phylogenetic tree of 481 hemagglutinin sequences from which the indices were calculated was constructed by maximum likelihood (Figure S1).
(0.11 MB PDF)

Figure S6 A scalar product test for phylogeographic concordance. Degrees difference between eigenvectors for a round robin comparison of migration matrices for the H5N1 hemagglutinin phylogeny of 429 sequences (HA429), the neuraminidase phylogeny of 429 sequences (NA429), and the hemagglutinin phylogeny of 481 sequences (HA481). For HA481 migration events to Burkina Faso were left out as no Burkina Faso neuraminidase sequences were available. For clarity's sake, only the real components of the differences in the eigenvectors are shown. The imaginary components, between eigenvectors 3 and 11 across the three comparisons, suggest pulsing transient diffusion for H5N1 phylogeography. Many of the eigenvectors differ orthogonally (by around 90 degrees) with the smallest average differences for HA429/NA429 and NA429/HA481.
(0.16 MB PDF)
