# Supplementary material for: Influenza A H5N1 Immigration Is Filtered Out at Some International Borders
Source: PLoS One. 2008 Feb 27;3(2):e1697. doi: 10.1371/journal.pone.0001697 (PMC2244808; doi:10.1371/journal.pone.0001697)
Supplement: Table S1 — Study H5N1 hemagglutinin (HA) and neuraminidase (NA) sequences by sample locality. (0.08 MB PDF) [file pone.0001697.s001.pdf]

Table S1. Study H5N1 hemagglutinin (HA) and neuraminidase (NA) sequences by sample locality.

Locality assigned	Isolate name	Hemagglutinin GenBank/IDB accession number	HA sites sequenced	Neuraminidase GenBank/IDB accession number	NA sites sequenced	Year isolated	Authors	China clade 2.3
Burkina Faso	A/chicken/Burkina Faso/5346-11/2006	EF090649	1207	-	-	2006	1	
Burkina Faso	A/chicken/Burkina Faso/5346-14/2006	EF090650	1176	-	-	2006	1	
Burkina Faso	A/hooded vulture/Burkina Faso/5346-10/2006	EF090648	1200	-	-	2006	1	
Burkina Faso	A/guinea fowl/Burkina Faso/5346-26/2006	EF090647	1181	-	-	2006	1	
Cote d'Ivoire	A/chicken/Cote d'Ivoire/1787-34/2006	CY016811	1722	CY016813	1344	2006	2	
Cote d'Ivoire	A/duck/Cote d'Ivoire/1787-18/2006	CY016803	1734	CY016805	1351	2006	2	
Crimea	A/cat/Dagestan/87/06	DQ864720	1779	-	-	2006	3	
Crimea	A/chicken/Volgograd/236/06	DQ864719	1779	-	-	2006	3	
Crimea	A/chicken/Krasnodar/199/06	DQ864718	1720	-	-	2006	3	
Crimea	A/chicken/Adygea/203/06	DQ864715	1751	-	-	2006	3	
Crimea	A/Cygnus olor/Astrakhan/Ast05-2-3/2005	DQ358746	1754	DQ358747	1374	2005	4	
Crimea	A/Cygnus olor/Astrakhan/Ast05-2-4/2005	DQ363918	1754	DQ363919	1373	2005	4	
Crimea	A/Cygnus olor/Astrakhan/Ast05-2-5/2005	DQ365004	1754	DQ365005	1373	2005	4	
Crimea	A/Cygnus olor/Astrakhan/Ast05-2-7/2005	DQ363923	1754	DQ363924	1373	2005	4	
Crimea	A/chicken/Crimea/08/2005	DQ650663	1754	DQ650665	1372	2005	5	
Crimea	A/chicken/Crimea/04/2005	DQ650659	1754	DQ650661	1372	2005	5	
Crimea	A/goose/Crimea/615/05	DQ864717	1766	-	-	2005	3	
Egypt	A/chicken/Egypt/2253-1/2006	CY020645	1734	CY020647	1358	2006	6	
Egypt	A/chicken/Egypt/10845-NAMRU3/2006	EF042622	1596	-	-	2006	7	
Egypt	A/Egypt/5614-NAMRU3/2006	EF042621	1596	-	-	2006	7	
Egypt	A/Egypt/3458-NAMRU3/2006	EF042619	1596	-	-	2006	7	
Egypt	A/Egypt/5494-NAMRU3/2006	EF042620	1596	-	-	2006	7	
Egypt	A/Egypt/3105-NAMRU3/2006	EF042618	1596	-	-	2006	7	
Egypt	A/Egypt/2947-NAMRU3/2006	EF042617	1596	-	-	2006	7	
Egypt	A/Egypt/2783-NAMRU3/2006	EF042615	1596	-	-	2006	7	
Egypt	A/Egypt/2763-NAMRU3/2006	EF042614	1596	-	-	2006	7	
Egypt	A/Egypt/12374-NAMRU3/2006	EF061116	1639	EF222324	1350	2006	7	
Egypt	A/duck/Egypt/2253-3/2006	CY016899	1743	CY016901	1352	2006	2	
Egypt	A/turkey/Egypt/5613NAMRU3-T/2006	DQ837590	1603	-	-	2006	8	
Egypt	A/Chicken/Egypt/5611NAMRU3-AN/2006	DQ837588	1613	-	-	2006	8	
Egypt	A/Chicken/Egypt/5612NAMRU3-S/2006	DQ837589	1604	-	-	2006	8	
Egypt	A/Egypt/2782-NAMRU3/2006	DQ464377	1640	-	-	2006	9	
Egypt	A/chicken/Egypt/960N3-004/2006	DQ447199	1673	-	-	2006	9	
Egypt	A/teal/Egypt/14051-NAMRU3/2005	EF042624	1596	-	-	2005	7	
Fujian	A/chicken/Fujian/584/2006	DQ992831	1695	EF124268	1341	2006	10	67
Fujian	A/duck/Fujian/668/2006	DQ992832	1662	EF124269	1326	2006	10	67
Fujian	A/chicken/Fujian/10313/2005	DQ992823	1665	EF124261	1347	2005	10	67
Fujian	A/chicken/Fujian/10567/2005	DQ992824	1668	EF124262	1065	2005	10	67
Fujian	A/chicken/Fujian/11933/2005	DQ992828	1695	EF124266	1347	2005	10	67
Fujian	A/chicken/Fujian/12239/2005	DQ992830	1680	EF124340	1269	2005	10	67
Fujian	A/duck/Fujian/12032/2005	DQ992829	1668	EF124267	1350	2005	10	67
Fujian	A/duck/Fujian/11094/2005	DQ992826	1695	EF124264	1296	2005	10	67
Fujian	A/chicken/Fujian/9821/2005	DQ992821	1695	EF124259	1326	2005	10	67
Fujian	A/chicken/Fujian/1042/2005	DQ320876	1698	DQ321008	1347	2005	11	
Fujian	A/Duck/Fujian/1734/05	DQ095629	1703	DQ095669	1357	2005	12	67
Fujian	A/duck/Fujian/897/2005	DQ320875	1695	DQ321007	1347	2005	11	
Fujian	A/goose/Fujian/bb/2003	DQ997405	1779	DQ997406	1458	2003	13	
Fujian	A/duck/Fujian/01/2002	AY585357	1707	AY585399	1357	2002	14	
Fujian	A/duck/Fujian/13/2002	AY585358	1707	AY585400	1357	2002	14	
Fujian	A/duck/Fujian/17/2001	AY585372	1708	AY585401	1418	2001	14	
Fujian	A/swine/Fujian/F1/2001	AY747617	1779	AY747618	1458	2001	15	
Fujian	A/duck/Fujian/19/2000	AY585359	1707	AY585402	1355	2000	14	
Guangdong	A/chicken/Shantou/1233/2006	DQ992779	1695	EF124245	1326	2006	10	67
Guangdong	A/chicken/Shantou/3840/2006	DQ992785	1692	EF124248	1338	2006	10	67
Guangdong	A/goose/Shantou/2086/2006	DQ992781	1671	EF124312	1404	2006	10	
Guangdong	A/goose/Shantou/3265/2006	DQ992783	1686	EF124246	1338	2006	10	67
Guangdong	A/goose/Shantou/3295/2006	DQ992784	1695	EF124247	1338	2006	10	67
Guangdong	A/goose/Shantou/239/2006	DQ992778	1662	EF124311	1386	2006	10	
Guangdong	A/Guinea fowl/Shantou/1341/2006	DQ992780	1698	EF124309	1338	2006	10	
Guangdong	A/pheasant/Shantou/2239/2006	DQ992782	1647	EF124212	1350	2006	10	49
Guangdong	A/Guangzhou/1/2006	DQ842489	1650	DQ842490	1296	2006	16	67
Guangdong	A/goose/Guangzhou/471/2006	DQ842487	1650	DQ842488	1296	2006	16	67
Guangdong	A/duck/Shantou/13323/2005	DQ992776	1695	EF124243	1338	2005	10	67
Guangdong	A/goose/Shantou/18442/2005	DQ992777	1647	EF124244	1350	2005	10	67
Guangdong	A/Chicken/Shantou/810/05	DQ095626	1693	DQ095666	1357	2005	12	49

Guangdong	A/Goose/Shantou/1621/05	DQ095628	1701	DQ095668	1356	2005	12	
Guangdong	A/goose/Shantou/2216/2005	DQ320915	1698	DQ321047	1347	2005	11	
Guangdong	A/Quail/Shantou/911/05	DQ095627	1703	DQ095667	1357	2005	12	49
Guangdong	A/chicken/Guangdong/174/04	AY609312	1779	AY609314	1397	2004	17	
Guangdong	A/chicken/Guangdong/178/04	AY737296	1776	AY737299	1350	2004	17	49
Guangdong	A/chicken/Guangdong/191/04	AY737289	1776	AY737291	1398	2004	17	
Guangdong	A/duck/Guangdong/173/04	AY737304	1776	AY737308	1398	2004	17	
Guangdong	A/duck/Shantou/4610/2003	DQ320914	1689	DQ321046	1347	2003	11	
Guangdong	A/duck/Guangdong/22/2002	AY585362	1708	AY585406	1412	2002	14	
Guangdong	A/goose/Guangdong/xb/2001	DQ997522	1779	DQ997523	1399	2001	13	
Guangdong	A/duck/Guangdong/01/2001	AY585360	1708	AY585403	1414	2001	14	
Guangdong	A/duck/Guangdong/07/2000	AY585373	1708	AY585404	1417	2000	14	
Guangdong	A/duck/Guangdong/12/2000	AY585361	1708	AY585405	1359	2000	14	
Guangdong	A/duck/Guangdong/40/2000	AY585374	1708	AY585407	1401	2000	14	
Guangdong	A/Goose/Guangdong/1/96	AF144305	1760	AF144304	1458	1996	18	
Guangxi	A/chicken/Guangxi/1212/2006	DQ993031	1683	EF124330	1350	2006	10	67
Guangxi	A/chicken/Guangxi/1951/2006	DQ993026	1695	EF124338	1350	2006	10	67
Guangxi	A/chicken/Guangxi/463/2006	DQ992745	1695	EF124272	1350	2006	10	67
Guangxi	A/chicken/Guangxi/683/2006	DQ993114	1695	EF124274	1350	2006	10	67
Guangxi	A/duck/Guangxi/1258/2006	DQ993030	1686	EF124331	1347	2006	10	67
Guangxi	A/duck/Guangxi/150/2006	DQ992742	1695	EF124229	1311	2006	10	67
Guangxi	A/duck/Guangxi/1830/2006	DQ993023	1695	EF124336	1350	2006	10	67
Guangxi	A/duck/Guangxi/2143/2006	DQ993022	1695	EF124339	1290	2006	10	67
Guangxi	A/duck/Guangxi/288/2006	DQ992744	1695	EF124231	1314	2006	10	67
Guangxi	A/duck/Guangxi/392/2006	DQ992746	1647	EF124232	1350	2006	10	67
Guangxi	A/duck/Guangxi/89/2006	DQ992741	1695	EF124209	1311	2006	10	49
Guangxi	A/goose/Guangxi/224/2006	DQ992743	1632	EF124230	1311	2006	10	67
Guangxi	A/goose/Guangxi/1458/2006	DQ993027	1695	EF124333	1350	2006	10	67
Guangxi	A/goose/Guangxi/1633/2006	DQ993025	1695	EF124335	1341	2006	10	67
Guangxi	A/goose/Guangxi/1898/2006	DQ993024	1695	EF124337	1350	2006	10	67
Guangxi	A/goose/Guangxi/52/2006	DQ992740	1665	-	-	2006	10	67
Guangxi	A/goose/Guangxi/532/2006	DQ993117	1695	EF124273	1350	2006	10	67
Guangxi	A/goose/Guangxi/582/2006	DQ992747	1647	EF124233	1350	2006	10	67
Guangxi	A/chicken/Guangxi/3154/2005	DQ992718	1695	EF124279	1308	2005	10	49
Guangxi	A/chicken/Guangxi/3791/2005	DQ99274	1695	EF124285	1308	2005	10	49
Guangxi	A/duck/Guangxi/2775/2005	DQ992714	1695	EF124275	1308	2005	10	
Guangxi	A/duck/Guangxi/2926/2005	DQ992715	1695	EF124276	1308	2005	10	49
Guangxi	A/duck/Guangxi/3085/2005	DQ992717	1695	EF124278	1308	2005	10	49
Guangxi	A/duck/Guangxi/3548/2005	DQ992721	1695	EF124282	1308	2005	10	49
Guangxi	A/duck/Guangxi/3741/2005	DQ992723	1695	EF124284	1308	2005	10	49
Guangxi	A/duck/Guangxi/3819/2005	DQ992725	1695	EF124286	1308	2005	10	49
Guangxi	A/duck/Guangxi/4016/2005	DQ992726	1695	EF124287	1308	2005	10	49
Guangxi	A/duck/Guangxi/4184/2005	DQ992727	1695	EF124288	1347	2005	10	49
Guangxi	A/duck/Guangxi/4196/2005	DQ992728	1695	EF124289	1341	2005	10	49
Guangxi	A/duck/Guangxi/4830/2005	DQ992733	1695	EF124222	1350	2005	10	67
Guangxi	A/duck/Guangxi/5270/2005	DQ992737	1695	EF124226	1350	2005	10	67
Guangxi	A/duck/Guangxi/5457/2005	DQ992739	1695	EF124228	1338	2005	10	67
Guangxi	A/goose/Guangxi/3017/2005	DQ992716	1695	EF124277	1308	2005	10	49
Guangxi	A/goose/Guangxi/3316/2005	DQ992719	1695	EF124280	1308	2005	10	49
Guangxi	A/goose/Guangxi/3714/2005	DQ992722	1695	EF124283	1308	2005	10	49
Guangxi	A/goose/Guangxi/4513/2005	DQ992731	1695	EF124221	1350	2005	10	67
Guangxi	A/duck/Guangxi/793/2005	DQ320899	1694	DQ321031	1347	2005	19	49
Guangxi	A/chicken/Guangxi/604/2005	DQ320898	1695	DQ321030	1347	2005	11	49
Guangxi	A/duck/Guangxi/951/2005	DQ320900	1695	DQ321032	1347	2005	11	49
Guangxi	A/goose/Guangxi/345/2005	DQ320896	1695	DQ321028	1323	2005	11	49
Guangxi	A/quail/Guangxi/575/2005	DQ320897	1694	DQ321029	1338	2005	11	49
Guangxi	A/chicken/Guangxi/2439/2004	DQ320893	1693	DQ321025	1395	2004	11	
Guangxi	A/chicken/Guangxi/2448/2004	DQ320894	1693	DQ321026	1368	2004	11	49
Guangxi	A/chicken/Guangxi/2461/2004	DQ320895	1690	DQ321027	1347	2004	11	49
Guangxi	A/duck/Guangxi/1311/2004	DQ320883	1689	DQ321015	1407	2004	11	
Guangxi	A/duck/Guangxi/1378/2004	DQ320884	1698	DQ321016	1407	2004	11	
Guangxi	A/duck/Guangxi/1586/2004	DQ320885	1698	DQ321017	1407	2004	11	
Guangxi	A/duck/Guangxi/1681/2004	DQ320886	1698	DQ321018	1407	2004	11	
Guangxi	A/duck/Guangxi/1793/2004	DQ320887	1698	DQ321019	1380	2004	11	
Guangxi	A/duck/Guangxi/2291/2004	DQ320890	1698	DQ321022	1407	2004	11	
Guangxi	A/duck/Guangxi/2396/2004	DQ320892	1698	DQ321024	1392	2004	11	
Guangxi	A/duck/Guangxi/351/2004	DQ320877	1695	DQ321009	1347	2004	11	49
Guangxi	A/duck/Guangxi/380/2004	DQ320878	1695	DQ321010	1326	2004	11	49
Guangxi	A/goose/Guangxi/1097/2004	DQ320881	1698	DQ321013	1380	2004	11	
Guangxi	A/goose/Guangxi/1832/2004	DQ320888	1671	DQ321020	1407	2004	11	
Guangxi	A/goose/Guangxi/2112/2004	DQ320889	1698	DQ321021	1407	2004	11	
Guangxi	A/goose/Guangxi/2383/2004	DQ320891	1698	DQ321023	1407	2004	11	
Guangxi	A/goose/Guangxi/914/2004	DQ320880	1695	DQ321012	1407	2004	11	
Guangxi	A/mallard/Guangxi/wt/2004	DQ997218	1779	DQ997211	1398	2004	13	
Guangxi	A/swine/Guangxi/wz/2004	DQ997262	1779	DQ997263	1399	2004	13	
Guangxi	A/duck/Guangxi/53/2002	AY585366	1708	AY585412	1361	2002	14	

Guangxi	A/duck/Guangxi/22/2001	AY585364	1708	AY585409	1414	2001	14	
Guangxi	A/duck/Guangxi/35/2001	AY585365	1708	AY585410	1414	2001	14	
Guangxi	A/duck/Guangxi/50/2001	AY585375	1708	AY585411	1354	2001	14	
Guangxi	A/duck/Guangxi/07/1999	AY585363	1708	AY585408	1421	1999	14	
Guizhou	A/chicken/Guiyang/1218/2006	DQ992772	1668	-	-	2006	10	
Guizhou	A/chicken/Guiyang/29/2006	DQ992763	1674	EF124239	1314	2006	10	67
Guizhou	A/chicken/Guiyang/441/2006	DQ992766	1668	EF124208	1326	2006	10	
Guizhou	A/chicken/Guiyang/846/2006	DQ992769	1698	-	-	2006	10	
Guizhou	A/duck/Guiyang/1260/2006	DQ992773	1665	-	-	2006	10	67
Guizhou	A/duck/Guiyang/1418/2006	DQ992775	1695	-	-	2006	10	67
Guizhou	A/duck/Guiyang/293/2006	DQ992764	1695	EF124240	1314	2006	10	67
Guizhou	A/duck/Guiyang/497/2006	DQ992767	1695	EF124241	1311	2006	10	67
Guizhou	A/goose/Guiyang/1175/2006	DQ992771	1668	-	-	2006	10	
Guizhou	A/goose/Guiyang/1304/2006	DQ992774	1695	-	-	2006	10	67
Guizhou	A/goose/Guiyang/337/2006	DQ992765	1668	EF124207	1320	2006	10	
Guizhou	A/chicken/Guiyang/2173/2005	DQ992752	1695	EF124211	1347	2005	10	49
Guizhou	A/chicken/Guiyang/3055/2005	DQ992755	1695	EF124305	1350	2005	10	
Guizhou	A/chicken/Guiyang/3570/2005	DQ992758	1662	EF124308	1326	2005	10	
Guizhou	A/chicken/Guiyang/3721/2005	DQ992759	1695	EF124237	1350	2005	10	67
Guizhou	A/chicken/Guiyang/4059/2005	DQ992762	1677	EF124238	1314	2005	10	67
Guizhou	A/duck/Guiyang/2231/2005	DQ992753	1644	EF124206	1347	2005	10	
Guizhou	A/duck/Guiyang/3009/2005	DQ992754	1596	EF124304	1326	2005	10	
Guizhou	A/duck/Guiyang/3242/2005	DQ992756	1695	EF124306	1350	2005	10	
Guizhou	A/duck/Guiyang/3834/2005	DQ992760	1647	EF124302	1350	2005	10	67
Guizhou	A/duck/Guiyang/3996/2005	DQ992761	1689	EF124303	1350	2005	10	67
Hebei	A/chicken/Hebei/326/2005	DQ343150	1707	DQ349118	1350	2005	20	
Hebei	A/chicken/Hebei/108/02	DQ343152	1707	DQ349116	1350	2002	20	
Hebei	A/chicken/Hebei/718/2001	DQ343151	1707	DQ349117	1410	2001	20	
Henan	A/swine/Henan/wy/2004	DQ997253	1779	DQ997254	1398	2004	13	
Henan	A/chicken/Henan/wu/2004	DQ997219	1779	DQ997220	1398	2004	13	
Henan	A/chicken/Henan/1/2004	DQ080022	1748	AY950244	1398	2004	21	
Henan	A/treesparrow/Henan/1/2004	AY741215	1707	AY741216	1350	2004	22	
Henan	A/treesparrow/Henan/2/2004	AY741217	1707	AY741218	1350	2004	22	
Henan	A/treesparrow/Henan/3/2004	AY741219	1707	AY741220	1350	2004	22	
Henan	A/treesparrow/Henan/4/2004	AY741221	1707	AY741222	1410	2004	22	
Henan	A/chicken/jiyuan/1/03	DQ211922	1707	DQ211926	1350	2003	23	
Henan	A/chicken/zhengzhou/1/02	DQ211923	1707	DQ211927	1350	2002	23	
Henan	A/chicken/zhoukou/2/02	DQ211924	1707	DQ211928	1350	2002	23	
Hong Kong	A/chicken/Hong Kong/282/2006	DQ992836	1677	EF124200	1350	2006	10	67
Hong Kong	A/chicken/Hong Kong/947/2006	DQ992841	1695	EF124205	1347	2006	10	67
Hong Kong	A/crested myna/Hong Kong/540/2006	DQ992838	1671	EF124202	1350	2006	10	67
Hong Kong	A/common magpie/Hong Kong/645/2006	DQ992839	1662	EF124203	1350	2006	10	67
Hong Kong	A/house crow/Hong Kong/2648/2006	DQ992848	1695	EF124196	1347	2006	10	67
Hong Kong	A/house crow/Hong Kong/2858/2006	DQ992849	1695	EF124197	1347	2006	10	67
Hong Kong	A/Japanese white-eye/Hong Kong/1038/2006	DQ992842	1692	EF124190	1338	2006	10	67
Hong Kong	A/robin/Hong Kong/75/2006	DQ992835	1692	EF124190	1338	2006	10	67
Hong Kong	A/grey heron/Hong Kong/728/2004	DQ320923	1677	DQ321055	1407	2004	11	49
Hong Kong	A/grey heron/Hong Kong/837/2004	DQ320924	1698	DQ321056	1398	2004	11	49
Hong Kong	A/egret/Hong Kong/757.2/03	AY676034	1707	AY676042	1410	2003	24	
Hong Kong	A/Hong Kong/213/03	AB212054	1779	AB212056	1458	2003	25	
Hong Kong	A/chicken/Hong Kong/61.9/02	AY575876	1707	AY575888	1092	2002	26	
Hong Kong	A/chicken/Hong Kongi/86.3/2002	DQ320927	1698	DQ321058	1074	2002	11	
Hong Kong	A/duck/Hong Kong/821/02	AY676033	1707	AY676041	1350	2002	26	
Hong Kong	A/Hong Kong/97/98	AF102676	1656	AF102661	1350	1998	27	
Hong Kong	A/Chicken/Hong Kong/220/97	AF046080	1741	AF046081	1370	1997	28	
Hong Kong	A/chicken/Hong Kong/258/97	AF057291	1718	AF057292	1318	1997	29	
Hong Kong	A/Chicken/Hong Kong/786/97	AF082035	1726	AF098549	1318	1997	30	
Hong Kong	A/Chicken/Hong Kong/728/97	AF046099	1741	AF098548	1318	1997	31	
Hong Kong	A/Chicken/Hong Kong/915/97	AF046100	1741	AF098550	1318	1997	31	
Hong Kong	A/Duck/Hong Kong/p46/97	AF098543	1726	AF098552	1318	1997	31	
Hong Kong	A/Goose/Hong Kong/w355/97	AF098545	1726	AF098554	1318	1997	31	
Hong Kong	A/Hong Kong/156/97	AF046088	1741	AF036357	1399	1997	32	
Hong Kong	A/Hong Kong/481/97	AF046096	1741	AF102663	1350	1997	37	
Hong Kong	A/Hong Kong/483/97	AF046097	1741	AF102668	1350	1997	37	
Hong Kong	A/Hong Kong/491/97	AF102677	1656	AF102665	1350	1997	37	
Hong Kong	A/Hong Kong/503/97	AF102679	1656	AF102666	1350	1997	37	
Hong Kong	A/Hong Kong/514/97	AF102682	1656	AF102669	1350	1997	37	
Hong Kong	A/Hong Kong/532/97	AF102680	1656	AF102667	1350	1997	37	
Hong Kong	A/Hong Kong/538/97	AF102674	1656	AF102662	1350	1997	37	
Hong Kong	A/Hong Kong/542/97	AF102678	1656	AF102670	1350	1997	37	
Hubei	A/chicken/Hubei/327/2004	AY684706	1779	AY684708	1398	2004	33	
Hubei	A/chicken/Hubei/489/2004	AY770079	1779	AY770078	1398	2004	34	

Hubei	A/chicken/Hubei/wn/2003	DQ997147	1778	DQ997150	1399	2003	13	
Hubei	A/chicken/Hubei/wo/2003	DQ997156	1778	DQ997157	1398	2003	13	
Hubei	A/duck/Hubei/wp/2003	DQ997163	1779	DQ997164	1398	2003	13	
Hubei	A/chicken/Hubei/wf/2002	DQ997087	1630	DQ997088	1458	2002	13	
Hubei	A/duck/Hubei/wg/2002	DQ997094	1779	DQ997095	1398	2002	13	
Hubei	A/chicken/Hubei/wh/1997	DQ997102	1779	DQ997103	1458	1997	13	
Hubei	A/chicken/Hubei/wi/1997	DQ997111	1640	DQ997112	1276	1997	13	
Hubei	A/chicken/Hubei/wj/1997	DQ997122	1779	DQ997115	1399	1997	13	
Hubei	A/chicken/Hubei/wl/1997	DQ997133	1779	DQ997134	1458	1997	13	
Hubei	A/chicken/Hubei/wm/1997	DQ997140	1591	DQ997143	836	1997	13	
Hunan	A/duck/Hunan/344/2006	DQ992791	1647	EF124254	1350	2006	10	67
Hunan	A/duck/Hunan/856/2006	DQ992792	1692	EF124255	1350	2006	10	67
Hunan	A/duck/Hunan/5152/2005	DQ992788	1695	EF124251	1347	2006	10	67
Hunan	A/chicken/Hunan/999/2005	DQ320910	1695	DQ321042	1347	2005	11	
Hunan	A/duck/Hunan/1265/2005	DQ320911	1695	DQ321043	1347	2005	11	49
Hunan	A/duck/Hunan/127/2005	DQ320902	1686	DQ321034	1336	2005	11	
Hunan	A/duck/Hunan/139/2005	DQ320903	1686	DQ321035	1336	2005	11	
Hunan	A/duck/Hunan/152/2005	DQ320905	1686	DQ321037	1321	2005	11	
Hunan	A/duck/Hunan/157/2005	DQ320906	1644	DQ321038	1336	2005	11	
Hunan	A/duck/Hunan/1608/2005	DQ320912	1695	DQ321044	1311	2005	11	49
Hunan	A/duck/Hunan/182/2005	DQ320909	1686	DQ321041	1336	2005	11	
Hunan	A/blackbird/Hunan/1/2004	AY741213	1707	AY741214	1350	2004	35	
Indonesia	A/feline/Indonesia/CDC1/2006	CY014208	1659	CY014234	1331	2006	36	
Indonesia	A/Indonesia/CDC326/2006	CY014204	1659	CY014239	1331	2006	36	
Indonesia	A/Indonesia/CDC329/2006	CY014206	1659	CY014235	1331	2006	36	
Indonesia	A/Indonesia/CDC357/2006	CY014207	1659	CY014230	1331	2006	36	
Indonesia	A/Indonesia/CDC370/2006	CY014209	1659	CY014231	1331	2006	36	
Indonesia	A/Indonesia/CDC390/2006	CY014213	1659	CY014233	1331	2006	36	
Indonesia	A/Indonesia/CDC582/2006	CY014384	1659	CY014386	1378	2006	36	
Indonesia	A/Indonesia/CDC595/2006	CY014280	1707	CY014282	1379	2006	36	
Indonesia	A/Indonesia/CDC597/2006	CY014296	1707	CY014298	1379	2006	36	
Indonesia	A/Indonesia/CDC599/2006	CY014303	1659	CY014305	1379	2006	36	
Indonesia	A/Indonesia/CDC610/2006	CY014393	1659	CY014398	1374	2006	36	
Indonesia	A/Indonesia/CDC623/2006	CY014401	1659	CY014406	1370	2006	36	
Indonesia	A/Indonesia/CDC625/2006	CY014433	1659	CY014438	1371	2006	36	
Indonesia	A/Indonesia/CDC634/2006	CY014441	1659	CY014446	1370	2006	36	
Indonesia	A/Indonesia/CDC699/2006	CY014497	1707	CY014502	1378	2006	36	
Indonesia	A/Indonesia/CDC742/2006	CY014537	1659	CY014539	1378	2006	36	
Indonesia	A/Indonesia/CDC7/2005	CY014177	1659	CY014179	1331	2005	36	
Indonesia	A/chicken/Indonesia/R60/05	AM183670	1779	AM183682	1378	2005	37	
Indonesia	A/chicken/Bantul/BBVet-I/2005	DQ320932	1698	DQ321063	1347	2005	3	
Indonesia	A/chicken/Salatiga/BBVet-I/2005	DQ320928	1698	DQ321059	1347	2005	3	
Indonesia	A/chicken/Wajo/BBVM/2005	DQ320933	1698	DQ321064	1338	2005	3	
Indonesia	A/chicken/Dairi/BPPVI/2005	DQ497667	1698	DQ493015	1350	2005	38	
Indonesia	A/chicken/Deli Serdang/BPPVI/2005	DQ497668	1698	DQ493016	1350	2005	38	
Indonesia	A/chicken/Gunung Kidal/BBVW/2005	DQ497651	1698	DQ493000	1350	2005	38	
Indonesia	A/chicken/Magetan/BBVW/2005	DQ497643	1698	DQ492992	1350	2005	38	
Indonesia	A/chicken/Purworejo/BBVW/2005	DQ497648	1698	DQ492997	1350	2005	38	
Indonesia	A/chicken/Simalanggang/BPPVI/2005	DQ497665	1698	DQ493013	1350	2005	38	
Indonesia	A/chicken/Tarutung/BPPVI/2005	DQ497669	1698	DQ493017	1350	2005	38	
Indonesia	A/chicken/Tebing Tinggi/BPPVI/2005	DQ497666	1698	DQ493014	1350	2005	38	
Indonesia	A/duck/Parepare/BBVM/2005	DQ497659	1689	DQ493008	1341	2005	38	
Indonesia	A/chicken/Jembrana/BPPV6/2004	DQ497657	1659	DQ493006	1079	2004	38	
Indonesia	A/chicken/Kulon Progo/BBVet-XII-2/2004	DQ497650	1695	DQ492999	1325	2004	38	
Indonesia	A/chicken/Pangkalpinang/BPPV3/2004	DQ497663	1659	DQ493012	1079	2004	38	
Indonesia	A/chicken/Purwakarta/BBVet-IV/2004	DQ497653	1698	DQ493002	1350	2004	38	
Indonesia	A/chicken/Kupang-1-NTT/BPPV6/2004	DQ497662	1695	DQ493011	1350	2004	38	
Indonesia	A/chicken/Kupang-3-NTT/BPPV6/2004	DQ497661	1698	DQ493010	1350	2004	38	
Indonesia	A/chicken/Ngawi/BPPV4/2004	DQ497644	1646	DQ492993	1079	2004	38	
Indonesia	A/quail/Boyolali/BPPV4/2004	DQ497647	1659	DQ492996	1079	2004	38	
Indonesia	A/quail/Tasikmalaya/BPPV4/2004	DQ497654	1659	DQ493003	1350	2004	38	
Indonesia	A/quail/Yogjakarta/BBVet-IX/2004	DQ497649	1686	DQ492998	1350	2004	38	
Indonesia	A/turkey/Kedaton/BPPV3/2004	DQ497664	1659	DQ493018	1350	2004	38	
Indonesia	A/Dk/Indonesia/MS/2004	AY651322	1697	AY651434	1350	2004	39	
Indonesia	A/Ck/Indonesia/4/2004	AY651324	1685	AY651437	995	2004	39	
Indonesia	A/chicken/Kulon Progo/BBVet-XII-1/2004	DQ320931	1688	DQ321062	1347	2004	11	
Indonesia	A/chicken/Yogjakarta/BBVet-IX/2004	DQ320930	1687	DQ321061	1347	2004	11	
Indonesia	A/chicken/Wonosobo/BPPV4/2003	DQ320929	1659	DQ321060	1079	2003	11	
Indonesia	A/chicken/Pekalongan/BPPV4/2003	DQ497645	1659	DQ492994	1079	2003	38	
Indonesia	A/chicken/Sragen/BPPV4/2003	DQ497646	1659	DQ492995	1079	2003	38	
Indonesia	A/chicken/Indonesia/R134/03	AM183669	1482	AM183681	1398	2003	37	
Indonesia	A/Ck/Indonesia/2A/2003	AY651323	1689	AY651435	1315	2003	39	
Indonesia	A/Ck/Indonesia/BL/2003	AY651321	1659	AY651432	1350	2003	39	
Indonesia	A/Ck/Indonesia/PA/2003	AY651320	1696	AY651433	1350	2003	39	

Iraq	A/human/Iraq/207-NAMRU3/2006	DQ435202	1647	-	-	2006	40
Iraq	A/domestic goose/Iraq/812/2006	DQ435201	1647	DQ835389	1349	2006	40
Iraq	A/domestic cat/Iraq/820/2006	DQ435200	1647	DQ835390	1349	2006	40
Italy	A/cygnus olor/Italy/742/2006	CY017035	1722	CY017037	1351	2006	2
Italy	A/mallard/Italy/835/2006	CY016795	1731	CY016797	1351	2006	2
Italy	A/mallard/Italy/3401/2005	CY021397	1722	CY021399	1419	2005	6
Japan	A/blow fly/Kyoto/93/2004	AB212649	1728	AB212650	1398	2004	41
Japan	A/chicken/Kyoto/3/2004	AB188824	1704	AB189047	1350	2004	42
Japan	A/chicken/Oita/8/2004	AB188816	1704	AB188818	1350	2004	42
Japan	A/chicken/Yamaguchi/7/2004	AB166862	1704	AB166864	1350	2004	42
Japan	A/crow/Kyoto/53/2004	AB189053	1704	AB189055	1350	2004	42
Japan	A/crow/Osaka/102/2004	AB189061	1704	AB189063	1350	2004	42
Japan	A/duck/Yokohama/aq10/2003	AB212280	1707	AB212282	1410	2003	43
Jilin	A/chicken/Jilin/hk/2004	DQ997325	1779	DQ997326	1457	2004	13
Jilin	A/chicken/Jilin/ha/2003	DQ997268	1779	DQ997271	1398	2003	13
Jilin	A/chicken/Jilin/hj/2003	DQ997318	1777	DQ997319	1458	2003	13
Jilin	A/chicken/Jilin/hn/2003	DQ997352	1538	DQ997345	1398	2003	13
Jilin	A/chicken/Jilin/ho/2003	DQ997355	1779	DQ997356	1313	2003	13
Jilin	A/chicken/Jilin/hp/2003	DQ997361	1677	DQ997364	1398	2003	13
Jilin	A/chicken/Jilin/hq/2003	DQ997370	1590	DQ997373	1305	2003	13
Jilin	A/chicken/Jilin/xw/2003	DQ997547	1779	DQ997548	1398	2003	13
Jilin	A/goose/Jilin/hb/2003	DQ997276	1779	DQ997277	1399	2003	13
Jilin	A/chicken/Jilin/hd/2002	DQ997283	1779	DQ997284	1398	2002	13
Jilin	A/chicken/Jilin/he/2002	DQ997291	1775	DQ997292	1397	2002	13
Jilin	A/chicken/Jilin/hf/2002	DQ997377	1779	DQ997378	1399	2002	13
Jilin	A/chicken/Jilin/hh/2002	DQ997308	1779	DQ997309	1398	2002	13
Jilin	A/chicken/Jilin/xv/2002	DQ997538	1779	DQ997539	1399	2002	13
Mongolia	A/bar-headed goose/Mongolia/1/05	AB233319	1737	AB239304	1365	2005	44
Mongolia	A/whooperswan/Mongolia/3/05	AB233320	1737	AB239311	1376	2005	44
Mongolia	A/whooperswan/Mongolia/4/05	AB233321	1710	AB239318	1363	2005	44
Mongolia	A/whooperswan/Mongolia/6/05	AB233322	1737	AB239325	1360	2005	44
Niger	A/chicken/Niger/2130-7/2006	DQ838517	1707	-		2006	45
Niger	A/chicken/Niger/2130-8/2006	DQ838516	1707	-		2006	45
Niger	A/duck/Niger/914/2006	CY017027	1732	CY017029	1352	2006	2
Nigeria	A/chicken/Nigeria/SO494/2006	AM262572	1718	-	-	2006	46
Nigeria	A/chicken/Nigeria/SO493/2006	AM262553	1718	-	-	2006	46
Nigeria	A/chicken/Nigeria/SO452/2006	AM262547	1718	-	-	2006	46
Nigeria	A/chicken/Nigeria/SO300/2006	AM262546	1713	-	-	2006	46
Nigeria	A/chicken/Nigeria/BA211/2006	AM262543	1727	-	-	2006	46
Nigeria	A/chicken/Nigeria/BA209/2006	AM262541	1687	-	-	2006	46
Nigeria	A/guinea fowl/Nigeria/957-12/2006	CY017179	1731	CY017181	1357	2006	2
Nigeria	A/ostrich/Nigeria/1047-25/2006	CY016915	1740	CY016917	1351	2006	2
Nigeria	A/chicken/Nigeria/1047-54/2006	CY016923	1734	CY016925	1379	2006	2
Nigeria	A/chicken/Nigeria/1047-62/2006	CY016931	1733	CY016933	1379	2006	2
Nigeria	A/chicken/Nigeria/1047-34/2006	CY016947	1733	CY016949	1351	2006	2
Nigeria	A/chicken/Nigeria/641/2006	CY016276	1732	CY016278	1356	2006	2
Nigeria	A/chicken/Nigeria/957-20/2006	CY016284	1731	CY016286	1357	2006	2
Novosibirsk	A/duck/Novosibirsk/56/2005	DQ230522	1754	DQ230524	1373	2005	47
Novosibirsk	A/grebe/Novosibirsk/29/2005	DQ230521	1754	DQ230523	1373	2005	47
Novosibirsk	A/duck/Novosibirsk/02/05	DQ864711	1779	DQ864710	1387	2005	48
Novosibirsk	A/wild duck/Omsk/103-01/05	DQ864721	1780	-	-	2005	48
Qinghai	A/Bar-headed goose/Qinghai/59/05	DQ095612	1707	DQ095652	1361	2005	12
Qinghai	A/Bar-headed goose/Qinghai/60/05	DQ095615	1701	DQ095655	1379	2005	12
Qinghai	A/Bar-headed goose/Qinghai/65/05	DQ095622	1697	DQ095662	1361	2005	12
Qinghai	A/black-headed goose/Qinghai/1/2005	DQ100554	1555	DQ100562	1195	2005	49
Qinghai	A/black-headed goose/Qinghai/2/2005	DQ100555	1555	DQ100563	1195	2005	49
Qinghai	A/black-headed gull/Qinghai/1/2005	DQ100556	1555	DQ100564	1195	2005	49
Qinghai	A/Brown-headed gull/Qinghai/3/05	DQ095616	1707	DQ095656	1362	2005	12
Qinghai	A/Great black-headed gull/Qinghai/1/2005	DQ100557	1554	DQ100565	1195	2005	49
Qinghai	A/bar-headed goose/Qinghai/0510/05	DQ137873	1679	DQ137874	1350	2005	50
Shanghai	A/duck/Shanghai/xj/2002	DQ997531	1779	DQ997532	1398	2002	13
Shanghai	A/duck/Shanghai/35/2002	AY585368	1707	AY585415	1363	2002	14
Shanghai	A/duck/Shanghai/37/2002	AY585369	1708	AY585416	1361	2002	14
Shanghai	A/duck/Shanghai/08/2001	AY585370	1708	AY585413	1357	2001	14
Shanghai	A/duck/Shanghai/13/2001	AY585367	1708	AY585414	1417	2001	14
Shanghai	A/duck/Shanghai/38/2001	AY585376	1708	AY585417	1355	2001	14
Sudan	A/chicken/Sudan/2115-12/2006	CY020677	1744	CY020679	1351	2006	6

Sudan	A/chicken/Sudan/2115-9/2006	CY020669	1741	CY020671	1358	2006	6	
Sudan	A/chicken/Sudan/1784-7/2006	CY016292	1734	CY016294	1357	2006	6	
Sudan	A/chicken/Sudan/1784-10/2006	CY016300	1734	CY016302	1351	2006	6	
Sudan	A/chicken/Sudan/1784/2006	DQ862003	1690	-	-	2006	45	
Thailand	A/chicken/Thailand/NP-172/2006	DQ999872	1717	DQ999873	1338	2006	51	67
Thailand	A/chicken/Thailand/PC-170/2006	DQ999887	1718	DQ999888	1347	2006	51	
Thailand	A/chicken/Thailand/PC-168/2006	DQ999880	1710	DQ999881	1174	2006	51	
Thailand	A/chicken/Thailand/Kanchanaburi/CK-160/2005	DQ334760	1761	DQ334762	1358	2005	52	
Thailand	A/chicken/Thailand/Nontaburi/CK-162/2005	DQ334776	1726	DQ334778	1346	2005	52	
Thailand	A/quail/Thailand/Nakhon Pathom/QA-161/2005	DQ334768	1726	DQ334770	1352	2005	52	
Thailand	A/Thailand/HA20/2005	DQ885618	1686	-	-	2005	53	
Thailand	A/Thailand/NKFE/2005	DQ885610	1711	DQ885611	1336	2005	53	
Thailand	A/Thailand/NKNP/2005	DQ885612	1670	DQ885613	1336	2005	53	
Thailand	A/Thailand/RPFE/2005	DQ885614	1698	DQ885615	1339	2005	53	
Thailand	A/Thailand/RPNP/2005	DQ885616	1655	DQ885617	1330	2005	53	
Thailand	A/Thailand/NK165/2005	DQ372591	1713	DQ372593	1372	2005	54	
Thailand	A/chicken/Bangkok/Thailand/CU-20/04	DQ083568	1666	DQ083604	1319	2004	55	
Thailand	A/chicken/Bangkok/Thailand/CU-3/04	DQ083551	1712	DQ083587	1347	2004	55	
Thailand	A/crow/Bangkok/Thailand/CU-25/04	DQ083570	1648	DQ083606	1347	2004	55	
Thailand	A/crow/Bangkok/Thailand/CU-35/04	DQ083575	1645	DQ083611	1340	2004	55	
Thailand	A/crow/Bangkok/Thailand/CU-4/04	DQ083552	1697	DQ083588	1347	2004	55	
Thailand	A/Kalji pheasant/Bangkok/Thailand/CU-18/04	DQ083566	1660	DQ083602	1296	2004	55	
Thailand	A/rollers/Bangkok/Thailand/CU-26/04	DQ083571	1652	DQ083607	1167	2004	55	
Thailand	A/chicken/Nakhon Sawan/Thailand/CU-12/04	DQ083560	1649	DQ083596	1317	2004	56	
Thailand	A/chicken/Nakhon Sawan/Thailand/CU-13/04	DQ083561	1638	DQ083597	1319	2004	56	
Thailand	A/chicken/Nakhon Sawan/Thailand/CU-39/04	DQ083577	1664	DQ083613	1347	2004	56	
Thailand	A/cat/Thailand/KU-02/04	DQ236077	1712	DQ236078	1367	2004	57	
Thailand	A/chicken/Chachoengsao/Thailand/CU-11/04	DQ083559	1670	DQ083595	1325	2004	58	
Thailand	A/chicken/Chachoengsao/Thailand/CU-10/04	DQ083558	1666	DQ083594	1317	2004	58	
Thailand	A/chicken/Chonburi/Thailand/CU-7/04	DQ083555	1668	DQ083591	1306	2004	58	
Thailand	A/chicken/Lopburi/Thailand/CU-38/04	DQ083576	1668	DQ083612	1347	2004	58	
Thailand	A/chicken/Nakhon Pathom/Thailand/CU-14/04	DQ083562	1649	DQ083598	1321	2004	58	
Thailand	A/chicken/Prachinburi/Thailand/CU-8/04	DQ083556	1656	DQ083592	1318	2004	58	
Thailand	A/chicken/Ratchaburi/Thailand/CU-68/04	DQ083578	1717	DQ083614	1347	2004	58	
Thailand	A/chicken/Suphanburi/Thailand/CU-1/04	DQ083550	1700	DQ083586	1347	2004	58	
Thailand	A/chicken/Saraburi/Thailand/CU-17/04	DQ083565	1713	DQ083601	1327	2004	58	
Thailand	A/chicken/Saraburi/Thailand/CU-27/04	DQ083572	1668	DQ083608	1347	2004	58	
Thailand	A/chicken/Suphanburi/Thailand/CU-9/04	DQ083557	1660	DQ083593	1317	2004	58	
Thailand	A/duck/Nakhon Pathom/Thailand/CU-71/04	DQ083579	1700	DQ083615	1300	2004	58	
Thailand	A/duck/Saraburi/Thailand/CU-74/04	DQ083581	1700	DQ083617	1313	2004	58	
Thailand	A/ostrich/Samut Prakan/Thailand/CU-31/04	DQ083574	1680	DQ083610	1306	2004	58	
Thailand	A/Ostrich/Samut Prakan/Thailand/CU-19/04	DQ083567	1669	DQ083603	1323	2004	58	
Thailand	A/pigeon/Samut Prakan/Thailand/CU-202/04	DQ083583	1712	DQ083619	1346	2004	58	
Thailand	A/white peafowl/Bangkok/Thailand/CU-29/04	DQ083573	1719	DQ083609	1302	2004	58	
Thailand	A/Ck/Thailand/73/2004	AY651327	1697	DQ076202	1357	2004	39	
Thailand	A/Ck/Thailand/9.1/2004	AY651328	1697	AY651440	1350	2004	39	
Thailand	A/Dk/Thailand/71.1/2004	AY651331	1697	AY651443	1327	2004	39	
Thailand	A/Gs/Thailand/79/2004	AY651332	1697	AY651444	1350	2004	39	
Thailand	A/Qa/Thailand/57/2004	AY651329	1697	AY651442	1350	2004	39	
Thailand	A/kalij pheasant/Thailand/CU-4/2004	AY590569	1626	-	-	2004	59	
Thailand	A/tiger/Thailand/CU-T6/04	AY972541	1726	AY972545	1347	2004	60	
Thailand	A/tiger/Thailand/CU-T4/04	AY972539	1718	AY972543	1347	2004	60	
Thailand	A/leopard/Suphanburi/Thailand/Leo-1/04	AY646175	1712	AY646176	1334	2004	61	
Thailand	A/tiger/Suphanburi/Thailand/Ti-1/04	AY646167	1712	AY646168	1334	2004	61	
Thailand	A/Thailand/4(SP-528)/2004	AY626143	1723	AY577316	1396	2004	62	
Thailand	A/Thailand/5(KK-494)/2004	AY627885	1732	AY627886	1377	2004	62	
Thailand	A/Thailand/LFPN-2004/2004	AY679514	1704	AY679513	1350	2004	63	
Vietnam	A/duck/Vietnam/568/2005	DQ320939	1695	DQ321070	1347	2005	11	49
Vietnam	A/duck/Vietnam/S654/2005	DQ320936	1698	DQ321067	1347	2005	11	
Vietnam	A/Hanoi/30408/2005	AB239125	1776	AB239126	1417	2005	64	
Vietnam	A/Vietnam/PEV16T/2005	DQ535724	1533	DQ535726	1350	2005	65	
Vietnam	A/Vietnam/CL2009/2005	DQ497729	1697	DQ493078	1332	2005	38	
Vietnam	A/Vietnam/CL119/2005	DQ497728	1693	DQ493077	1326	2005	38	
Vietnam	A/Vietnam/CL115/2005	DQ497727	1689	DQ493076	1350	2005	38	
Vietnam	A/Vietnam/CL105/2005	DQ497726	1689	DQ493075	1350	2005	38	
Vietnam	A/wild bird/Vietnam/434/2005	DQ497705	1695	DQ493054	1350	2005	38	
Vietnam	A/quail/Vietnam/282/2005	DQ497711	1695	DQ493060	1332	2005	38	
Vietnam	A/duck/Vietnam/543/2005	DQ497717	1695	DQ493066	1350	2005	38	
Vietnam	A/duck/Vietnam/557/2005	DQ497709	1695	DQ493058	1350	2005	38	
Vietnam	A/duck/Vietnam/283/2005	DQ497708	1695	DQ493057	1348	2005	38	
Vietnam	A/duck/Vietnam/376/2005	DQ497704	1695	DQ493053	1350	2005	38	
Vietnam	A/chicken/Vietnam/393/2005	DQ497712	1695	DQ493061	1350	2005	38	
Vietnam	A/chicken/Vietnam/398/2005	DQ497703	1695	DQ493052	1350	2005	38	
Vietnam	A/chicken/Viet Nam/10/2005	CY016867	1731	CY016869	1351	2005	2	49
Vietnam	A/chicken/Viet Nam/17/2005	CY017059	1731	CY017061	1350	2005	2	49

Vietnam	A/chicken/Viet Nam/2/2005	CY016835	1731	CY016837	1357	2005	2	
Vietnam	A/chicken/Viet Nam/6/2005	CY016843	1729	CY016845	1351	2005	2	
Vietnam	A/duck/Viet Nam/1/2005	CY016827	1731	CY017189	1379	2005	2	
Vietnam	A/duck/Viet Nam/12/2005	CY016883	1731	CY016885	1351	2005	2	49
Vietnam	A/duck/Viet Nam/20/2005	CY016891	1731	CY016893	1379	2005	2	
Vietnam	A/duck/Viet Nam/18/2005	CY017067	1731	CY017069	1350	2005	2	
Vietnam	A/quail/Viet Nam/15/2005	CY017051	1728	CY017053	1364	2005	2	49
Vietnam	A/duck/Vietnam/TG24-O1/05	AM183677	1776	AM183678	1398	2005	37	
Vietnam	A/chicken/Viet Nam/DT-171/2004	DQ099759	1707	DQ321068	1347	2004	66	
Vietnam	A/Hatay/2004	AJ867074	1707	AJ867075	1366	2004	67	
Vietnam	A/Mallard duck/Vietnam/133/2004	DQ320940	1698	-	-	2004	68	
Vietnam	A/quail/Vietnam/36/04	AY818137	1707	AY818143	1350	2004	69	
Vietnam	A/VietNam/1203/2004	AY818135	1707	AY651447	1350	2004	69	
Vietnam	A/chicken/Vietnam/C58/04	AY818136	1707	AY818142	1350	2004	69	
Vietnam	A/Vietnam/CL100/2004	DQ497725	1633	DQ493074	1350	2004	38	
Vietnam	A/Vietnam/CL36/2004	DQ497724	1697	DQ493073	1350	2004	38	
Vietnam	A/Vietnam/CL26/2004	DQ497723	1696	DQ493072	1326	2004	38	
Vietnam	A/Vietnam/CL20/2004	DQ497722	1695	DQ493071	1350	2004	38	
Vietnam	A/Vietnam/CL02/2004	DQ497720	1626	DQ493069	1350	2004	38	
Vietnam	A/Vietnam/CL01/2004	DQ497719	1696	DQ493068	1350	2004	38	
Vietnam	A/chicken/Vietnam/52/2004	DQ497698	1695	DQ493047	1350	2004	38	
Vietnam	A/chicken/Vietnam/132/2004	DQ497718	1695	DQ493067	1350	2004	38	
Vietnam	A/chicken/Vietnam/133/2004	DQ497700	1695	DQ493049	1338	2004	38	
Vietnam	A/chicken/Vietnam/134/2004	DQ497714	1695	DQ493063	1350	2004	38	
Vietnam	A/chicken/Vietnam/159/2004	DQ497713	1695	DQ493062	1350	2004	38	
Vietnam	A/chicken/Vietnam/53/2004	DQ497710	1695	DQ493059	1350	2004	38	
Vietnam	A/chicken/Vietnam/147/2004	DQ497701	1695	DQ493050	1350	2004	38	
Vietnam	A/duck/Vietnam/148/2004	DQ497702	1695	DQ493051	1339	2004	38	
Vietnam	A/quail/Vietnam/177/2004	DQ497715	1695	DQ493064	1350	2004	38	
Vietnam	A/chicken/Vietnam/27/2003	DQ320938	1698	DQ321069	1322	2003	11	
Yunnan	A/duck/Yunnan/1126/2006	DQ992813	1662	-	-	2006	10	49
Yunnan	A/goose/Yunnan/1136/2006	DQ992814	1695	-	-	2006	10	67
Yunnan	A/goose/Yunnan/1143/2006	DQ992815	1674	-	-	2006	10	67
Yunnan	A/goose/Yunnan/1144/2006	DQ992816	1695	-	-	2006	10	67
Yunnan	A/goose/Yunnan/1338/2006	DQ992817	1695	-	-	2006	10	67
Yunnan	A/goose/Yunnan/1396/2006	DQ992818	1695	-	-	2006	10	67
Yunnan	A/Chicken/Yunnan/447/05	DQ095624	1698	DQ095664	1335	2005	12	
Yunnan	A/duck/Yunnan/4400/2005	DQ992797	1695	EF124293	1347	2005	10	49
Yunnan	A/duck/Yunnan/4589/2005	DQ992799	1695	EF124295	1347	2005	10	49
Yunnan	A/duck/Yunnan/5133/2005	DQ992801	1698	EF124217	1347	2005	10	
Yunnan	A/duck/Yunnan/5251/2005	DQ992803	1695	EF124213	1344	2005	10	49
Yunnan	A/duck/Yunnan/5877/2005	DQ992807	1650	EF124297	1347	2005	10	49
Yunnan	A/duck/Yunnan/6607/2005	DQ992812	1647	EF124216	1350	2005	10	49
Yunnan	A/goose/Yunnan/3315/2005	DQ992794	1689	EF124310	1347	2005	10	
Yunnan	A/goose/Yunnan/3720/2005	DQ992795	1695	EF124291	1347	2005	10	49
Yunnan	A/goose/Yunnan/4129/2005	DQ992796	1695	EF124292	1347	2005	10	49
Yunnan	A/goose/Yunnan/4494/2005	DQ992798	1695	EF124294	1344	2005	10	49
Yunnan	A/goose/Yunnan/4804/2005	DQ992800	1695	EF124296	1347	2005	10	49
Yunnan	A/goose/Yunnan/5539/2005	DQ992805	1650	EF124218	1350	2005	10	
Yunnan	A/goose/Yunnan/6027/2005	DQ992808	1644	EF124298	1329	2005	10	49
Yunnan	A/goose/Yunnan/6368/2005	DQ992811	1647	EF124215	1350	2005	10	49
Yunnan	A/goose/Yunnan/6169/2005	DQ992809	1623	-	-	2005	10	
Outgroup	A/chicken/Scotland/59	X07869	1768	AJ416625	1445	1959	70, 71	

The list includes full sample name, GenBank accession number, number of nucleotide sites originally sequenced, year of sample, sequencing group (authors), and membership in clade 2.3 samples of 49 and 67 isolates described in the text.

1. Ducatez, M.F., Tarnagda, Z., Tahita, M.C., Sow, A., De Landtsheer, S., Londt, B.Z., Brown, I.H., Osterhaus, A.D.M.E., Fouchier, R.A.M., Ouedraogo, J.B. and Muller,C.P. (2007) Genetic Characterization of HPAI (H5N1) Viruses from Poultry and Wild Vultures, Burkina Faso. *Emerging Infect. Dis.* 13: 611-613.

2. Spiro, D., Sengamalay, N., Boyne, A., Halpin, R., Wang, S., Ghedin, E., Zaborsky, J., Subbu, V., Sparenborg, J., Gallagher, T., Overton, L., Liu, X., Salzberg, S.L., Sitz, J., Katzel, D., Neupane, R., Shumway, M., Koo, H., Capua, I., Cattoli, G., Couacy-Hymann, E., Bao, Y., Bolotov, P., Dernovoy, D., Kiryutin, B., Lipman, D.J. and Tatusova, T. The NIAID Influenza Genome Sequencing Consortium. Submitted (10-OCT-

2006) on behalf of TIGR/Istituto Zooprofilattico Sperimentale delle Venezie/NCBI, National Center for Biotechnology Information, NIH, Bethesda, MD 20894, USA.

3. Scherbakova, L.O., Pchelkina, I.P., Andriyasov, A.V., Kolosov, S.N., Manin, T.B., Drygin, V.V. and Gruzdev, K.N. Direct Submission. Submitted (20-JUL-2006) Laboratory for Diagnosis of Avian Diseases, Federal Centre for Animal Health (FGI ARRIAH), Yur'evets, Vladimir 600901, Russia.

4. Lyapina, O. V., Usachev, E. V. & Prilipov, A.G. Direct submission. Submitted (06-FEB-2006) Molecular Genetic Laboratory, Ivanovsky Virology Institute RAMS, Gamalei 16, Moscow 123098, Russia.

5. Ternovoi, V.A., Agafonov, A.P., Grudinin, M.P., Blinov, V.M., Sergeev, A.N., Netesov, S.V. and Kiselev, O.I. Direct Submission. Submitted (25-MAY-2006) Institute of Influenza, 197376 Prof. Popova Str., 15/17 Saint Petersburg, SRV VB Vector, Koltsovo 630559, Russia.

6. Spiro, D., Sengamalay, N., Boyne, A., Halpin, R., Wang, S., Ghedin, E., Zaborsky, J., Subbu, V., Sparenborg, J., Gallagher, T., Overton, L., Liu, X., Salzberg, S.L., Sitz, J., Katzel, D., Neupane, R., Shumway, M., Koo, H., Capua, I., Cattoli, G., Mona, M.A., Bao, Y., Bolotov, P., Dernovoy, D., Kiryutin, B., Lipman, D.J. and Tatusova, T. The NIAID Influenza Genome Sequencing Consortium. Direct Submission. Submitted (22-MAR-2007) on behalf of TIGR/Istituto Zooprofilattico Sperimentale delle Venezie/NCBI, National Center for Biotechnology Information, NIH, Bethesda, MD 20894, USA.

7. Saad, M.D., Gamal-Eldein, M.A., Ahmed, L.S., Yingst, S.L., Parker, M.A. and Monteville, M.R. Direct Submission. Submitted (04-OCT-2006) Viral and Zoonotic Diseases Research Program, U.S. Naval Medical Research Unit No. 3, Extension of Ramses Street, Nasr City, Cairo 11517, Egypt.

8. Saad, M.D., Boynton, B.R., Earhart, K.C., Taha, M.M., Nassif, S.A., Ali, A.M., Mansour, M., Labib, E.M. and Monteville, M.R. Direct Submission. Submitted (06-JUL-2006) Viral and Zoonotic Diseases Research Program, United States Naval Medical Research Unit No. 3, Extension of Ramses Street, Nasr City, Cairo 11517, Egypt.

9. Saad, M.D., Boynton, B.R., Earhart, K.C., Elsayed, N.M., Yingst, S.L., Elassal, E.M. and Monteville, M.R. Direct Submission. Submitted (28-MAR-2006) Viral and Zoonotic Diseases Research Program, U.S. Naval Medical Research Unit No. 3, Extension of Ramses Street, Nasr City, Cairo 11517, Egypt.

10. Smith, G.J., Fan, X.H., Wang, J., Li, K.S., Qin, K., Zhang, J.X., Vijaykrishna, D., Cheung, C.L., Huang, K., Rayner, J.M., Peiris, J.S., Chen, H., Webster, R.G. and Guan, Y. (2006) Emergence and predominance of an H5N1 influenza variant in China. *Proc. Natl. Acad. Sci.* U.S.A. 103: 16936-16941.

11. Smith, G. J. D., Chen, H. & Guan, Y. Direct submission. Submitted (07-DEC-2005) State Key Laboratory of Emerging Infectious Diseases, Department of Microbiology, The University of Hong Kong, Sassoon Road, Pokfulam, Hong Kong SAR, China.

12. Chen, H., Smith, G. J. D., Zhang, S. Y., Qin, K., Wang, J., Li, K. S., Webster, R. G., Peiris, J. S. M. & Guan, Y. (2005) *Nature* 436: 191-192.

13. Liao, X., Zhang, X., Wang, J., Yu, J. and Liu, J. Direct Submission. Submitted (18-SEP-2006) Beijing Genomics Institute, Chinese Academy of Sciences, B-6, Beijing Airport Industrial Zone, Beijing 101300, China.

14. Chen, H., Deng, G., Li, Z., Tian, G., Li, Y., Jiao, P., Zhang, L., Liu, Z., Webster, R. G. & Yu, K. (2004) *Proc. Natl. Acad. Sci. U.S.A.* 101: 10452-10457.

15. Li, H. Y., Yu, K. Z., Yang, H. L., Xin, X. G., Chen, J. Y., Zhao, P., Bi, Y. Z. & Chen, H. L. Direct submission. Submitted (10-SEP-2004). National Key Laboratory of Veterinary Biotechnology and Animal Influenza Laboratory of the Ministry of Agriculture, Harbin Veterinary Research Institute of Chinese Academy of Agriculture Sciences, 427 Maduan Street, Nangang District.

16. Wang M, Di B, Zhou DH, Zheng BJ, Jing H, Lin YP, Liu YF, Wu XW, Qin PZ, Wang YL, Jian LY, Li XZ, Xu JX, Lu EJ, Li TG, Xu J. (2006) Food markets with live birds as source of avian influenza. *Emerg Infect Dis.* 12: 1773-5.

17. Wan, X. F., Ren, T., Luo, K. J., Liao, M., Zhang, G. H., Chen, J. D., Cao, W. S., Li, Y., Jin, N. Y., Xu, D. & Xin, C. A. (2005) *Arch Virol.* 150: 1257-1266.

18. Xu, X., Subbarao, K., Cox, N. J. & Guo, Y. (1999) *Journal of Virology*. 261: 15-19.

19. Chen, H., Smith, G. J., Li, K. S., Wang, J., Fan, X. H., Rayner, J. M., Vijaykrishna, D., Zhang, J. X., Zhang, L. J., Guo, C. T., *et al*. (2006) *Proc Natl Acad Sci U S A.* 103: 2845-2850.

20. He, G. M., Qiao, J. H. & Dong, C. G. Direct submission. Submitted (02-JAN-2006) College of Veterinary Medicine, China Agriculture University, Yuan Ming Yuan West Street, Beijing 100094, China.

21. Hu, J., Chen, Z., Zhang, X. & Yu, J. Direct submission. Submitted (28-FEB-2005). Beijing Genomics Institute, Beijing Airport Industrial Zone B6: Beijing 086/101300, China.

22. Kou, Z., Lei, F. M., Yu, J., Fan, Z. J., Yin, Z. H., Jia, C. X., Xiong, K. J., Sun, Y. H., Zhang, X. W., Wu, X. M., *et al*. (2005) *J. Virol.* 79: 15460-15466.

23. Chen, H.-Y., Cui, B.-A., Wei, Z.-Y., Li, P., Liu, Z.-T., Jin, X.-X. & Niu, C.-L. Direct submission. Submitted (15-SEP-2005). The College of Animal Husbandry, Henan Agricultural University, Wenhualu, Zhengzhou, Henan 450002, China.

24. Lee, C. W., Suarez, D. L., Tumpey, T. M., Sung, H. W., Kwon, Y. K., Lee, Y. J., Choi, J. G., Joh, S. J., Kim, M. C., Lee, E. K., *et al*. (2005) *J. Virol.* 79: 3692-3702.

25. Shinya, K., Hatta, M., Yamada, S., Takada, A., Watanabe, S., Halfmann, P., Horimoto, T., Neumann, G., Kim, J. H., Lim, W., *et al*. (2005) *J. Virol.* 79: 9926-9932.

26. Guan, Y., Poon, L. L., Cheung, C. Y., Ellis, T. M., Lim, W., Lipatov, A. S., Chan, K. H., Sturm-Ramirez, K. M., Cheung, C. L., Leung, Y. H., *et al.* (2004) *Proc. Natl. Acad. Sci. U.S.A.* 101: 8156-8161.

27. Bender, C., Hall, H., Huang, J., Klimov, A., Cox, N., Hay, A., Gregory, V., Cameron, K., Lim, W. and Subbarao, K. (1999) *Virology*. 254: 115-123.

28. Suarez, D. L., Perdue, M. L., Cox, N., Rowe, T., Bender, C., Huang, J., Swayne, D. E. (1998) *J. Virol.* 72: 6678-6688.

29. Claas, E. C., Osterhaus, A. D., van Beek, R., De Jong, J. C., Rimmelzwaan, G. F., Senne, D. A., Krauss, S., Shortridge, K. F. & Webster, R. G. (1998) *Lancet*. 351: 472-477.

30. Matrosovich, M., Zhou, N., Kawaoka, Y. and Webster, R. (1999) The surface glycoproteins of H5 influenza viruses isolated from humans, chickens, and wild aquatic birds have distinguishable properties. *J. Virol.* 73: 1146-1155.

31. Zhou, N. N., Shortridge, K. F., Claas, E. C. J., Krauss, S. L. & Webster, R. G. (1999) *J. Virol.* 73: 3366-3374.

32. Subbarao, K., Klimov, A., Katz, J., Regnery, H., Lim, W., Hall, H., Perdue, M., Swayne, D., Bender, C., Huang, J., *et al.* (1998) *Science*. 279: 393-396.

33. Zhou, H., Jin, M., Chen, H., Huag, Q. and Yu, Z. Genome-sequence Analysis of the Pathogenic H5N1 Avian Influenza A Virus Isolated in China in 2004. (2006) *Virus Genes* 32: 85-95.

34. Wu, S., Xu, J., Qu, S., Zheng, C., Xiao, G. and Tian, B. Direct Submission. Submitted (04-OCT-2004) School of Life Science, Wuhan University, Luojiashan, Wuhan, Hubei 430072, China.

35. Li, T., Kou, Z., Chen, S., Fan, Z., Xiong, K. & Zhang, Z. Direct submission. Submitted (02-SEP-2004) China Center For General Virus Culture Collection, Wuhan Institute of Virology, CAS, Xiaohongshan 44, Wuhan, Hubei 430071, China.

36. Balish, A., Bright, R., Deyde, V., Garten, R., Jadhao, S., Lindstrom, S., Loftin, L., Matsuoka, Y., Shaw, M., Shu, B., Smith, C., Smith, E. and Xu, X. Direct Submission. Submitted (30-AUG-2006) WHO Collaborating Center for Surveillance, Epidemiology and Control of Influenza, Influenza Branch, Centers for Disease Control and Prevention, 1600 Clifton Road NE, Atlanta, GA 30333, USA. In collaboration with the Indonesian Ministry of Health.

37. Starick, E., Werner, O., Harder, T. and Beer, M. Direct Submission. Submitted (29-JAN-2006) Starick E., Fed Res Inst for Animal Health, Fr.-Loeffler-Institut, Boddenblick 5a, 17493 Greifswald-Insel Riems, GERMANY.

38. Smith, G.J., Naipospos, T.S., Nguyen, T.D., de Jong, M.D., Vijaykrishna, D., Usman, T.B., Hassan, S.S., Nguyen, T.V., Dao, T.V., Bui, N.A., Leung, Y.H., Cheung, C.L., Rayner, J.M., Zhang, J.X., Zhang, L.J., Poon, L.L., Li, K.S., Nguyen, V.C., Hien, T.T., Farrar, J., Webster, R.G., Chen, H., Peiris, J.S. and Guan, Y. (2006) Evolution and adaptation of H5N1 influenza virus in avian and human hosts in Indonesia and Vietnam. *Virology* 350: 258-268.

39. Li, K.S., Guan, Y., Wang, J., Smith, G.J., Xu, K.M., Duan, L., Rahardjo, A.P., Puthavathana, P., Buranathai, C., Nguyen, T.D., Estoepangestie, A.T., Chaisingh, A., Auewarakul, P., Long, H.T., Hanh, N.T., Webby, R.J., Poon, L.L., Chen, H., Shortridge, K.F., Yuen, K.Y., Webster, R.G. and Peiris, J.S. (2004) Genesis of a highly pathogenic and potentially pandemic H5N1 influenza virus in eastern Asia. *Nature* 430: 209-213.

40. Yingst, S.L., Saad, M.D. and Felt, S.A. (2006) Qinghai-like H5N1 from Domestic Cats, Northern Iraq. *Emerging Infect. Dis.* 12 (8), 1295-1297.

41. Sawabe, K., Hoshino, K., Isawa, H. & Sasaki, T. Direct submission. Submitted (28-APR-2005) Kyoko Sawabe, National Institute of Infectious Diseases, Department of Medical Entomology; Toyama 1-23-1, Shinjuku-ku, Tokyo, 162-8640, Japan (E-mail:sawabe@nih.go.jp, Tel:81-3-5285-1111(ex.2423), Fax:81-3-5285-1147).

42. Mase, M., Tsukamoto, K., Imada, T., Imai, K., Tanimura, N., Nakamura, K., Yamamoto, Y., Hitomi, T., Kira, T., Nakai, T., *et al.* (2005) *Virology* 332: 167-176.

43. Mase, M., Eto, M., Tanimura, N., Imai, K., Tsukamoto, K., Horimoto, T., Kawaoka, Y., Yamaguchi, S. (2005) *Virology*. 339: 101-109.

44. Onishi, S., Kida, H. & Sakoda, Y. Direct submission. Submitted (21-OCT-2005). Satoko Onishi, Hokkaido University, Graduate School of Veterinary Medicine; kita-ku, kita18 nisi9: Sapporo, Hokkaido 060-0818, Japan (E-mail:onishi@vetmed.hokudai.ac.jp, Tel:81-11-706-5209: Fax:81-11-706-5273).

45. Capua, I., Aboubackar, S., Cattoli, G. and De Benedictis, P. Direct Submission. Submitted (07-JUL-2006) Virology, Istituto Zooprofilattico Sperimentale delle Venezie, OIE/FAO and National Reference Laboratory for Avian Influenza and New Castle Disease, Viale dell Universita 10: Legnaro, PD 35020, Italy.

46. Ducatez, M.F., Olinger, C.M., Owoade, A.A., De Landtsheer, S., Ammerlaan, W., Niesters, H.G., Osterhaus, A.D., Fouchier, R.A. and Muller, C.P. (2006) Avian flu: multiple introductions of H5N1 in Nigeria. *Nature* 442: 37.

47. Prilipov, A. G., Sadykova, G. K. & Lyapina, O.V. Direct submission. Submitted (04-OCT-2005). Molecular Genetics, Ivanovsky Virology Institute RAMS, Gamalei 16, Moscow 123098, Russia.

48. Scherbakova, L.O., Pchelkina, I.P., Andriyasov, A.V., Kolosov, S.N., Manin, T.B., Drygin, V.V. and Gruzdev, K.N. Direct Submission. Submitted (20-JUL-2006) Laboratory for Diagnosis of Avian Diseases, Federal Centre for Animal Health (FGI ARRIAH), Yur'evets, Vladimir 600901, Russia.

49. Liu, J., Xiao, H., Lei, F., Zhu, Q., Qin, K., Zhang, X. W., Zhang, X. L., Zhao, D., Wang, G., Feng, Y., *et al.* (2005) *Science*. 309: 1206.

50. Zhou, J.Y., Shen, H.G., Chen, H.X., Tong, G.Z., Liao, M., Yang, H.C. and Liu, J.X. (2006) Characterization of a highly pathogenic H5N1 influenza virus derived from bar-headed geese in China. *J. Gen. Virol*. 87: 1823-1833.

51. Chutinimitkul, S., Songserm, T., Amonsin, A., Payungporn, S.,Suwannakarn, K., Damrongwatanapokin, S., Chaisingh, A., Nuansrichay, B., Chieochansin, T., Theamboonlers, A. and Poovorawan, Y. (2007) New Strain of Influenza A Virus (H5N1), Thailand. *Emerging Infect. Dis.* 13: 506-507.

52. Amonsin, A., Chutinimitkul, S., Pariyothorn, N., Songserm, T.,Damrongwantanapokin, S., Puranaveja, S., Jam-On, R., Sae-Heng, N., Payungporn, S., Theamboonlers, A., Chaisingh, A., Tantilertcharoen, R., Suradhat, S., Thanawongnuwech, R. and Poovorawan, Y. (2006) Genetic characterization of influenza A viruses (H5N1) isolated from 3rd wave of Thailand AI outbreaks. *Virus Res.* 122: 194-199.

53. Sutthent, R. Direct Submission. Submitted (03-AUG-2006) Department of Microbiology, Faculty of Medicine Siriraj Hospital, Mahidol University, 2 Prannok Rd; Bangkok-noi, Bangkok 10700, Thailand.

54. Chutinimitkul, S., Bhattarakosol, P., Srisuratanon, S., Eiamudomkan, A., Kongsomboon, K., Damrongwatanapokin, S., Chairingh, A., Suwannakarn, K., Chieochansin, T., Theamboonlers, A. and Poovorawan, Y. (2006) H5N1 Influenza A Virus and Infected Human Plasma. *Emerging Infect. Dis*. 12: 1041-1043.

55. Amonsin, A., Pariyothorn, N., Tantilertcharoen, R., Thanawongnuwech, R., Viseshakul, N., Suradhat, S., Wongyanin, P., Payungporn, S., Chutinimitkul, S., Theamboonlers, A. & Poovorawan, Y. Direct submission. Submitted (06-OCT-2004) Chulalongkorn University, Rama 4 Road, Pathumwan, Bangkok 10330, Thailand.

56. Amonsin, A., Pariyothorn, N., Payungporn, S., Theamboonlers, A., Chutinimitkul, S., Tantilertcharoen, R., Wongyanin, P., Thanawongnuwech, R., Suradhat, S., Chaisingh, A., Buranathai, C. & Poovorawan, Y. Direct submission. Submitted (02-JUN-2005) Chulalongkorn University, Rama 4 Rd., Pathumwan, Bangkok 10330, Thailand.

57. Songserm, T., Amonsin, A., Jam-on, R., Sae-Heng, N., Meemak, N., Pariyothorn, N., Payungporn, S., Theamboonlers, A. and Poovorawan, Y. (2006) Avian Influenza H5N1 in Naturally Infected Domestic Cat. *Emerging Infect. Dis.* 12: 681-683.

58. Amonsin, A., Pariyothorn, N., Payungporn, S., Theamboonlers, A., Chutinimitkul, S., Tantilertcharoen, R., Wongyanin, P., Thanawongnuwech, R., Suradhat, S., Chaisingh, A., Buranathai, C. and Poovorawan, Y. Direct Submission. Submitted (02-JUN-2005) Chulalongkorn University, Rama 4 Rd., Pathumwan, Bangkok 10330, Thailand.

59. Payungporn, S., Chutinimitkul, S., Chaisingh, A., Damrongwantanapokin, S., Buranathai, C., Amonsin, A., Theamboonlers, A. and Poovorawan, Y. (2006) Single step multiplex real-time RT-PCR for H5N1 influenza A virus detection. *J. Virol. Methods* 131: 143-147.

60. Amonsin, A., Payungporn, S., Theamboonlers, A., Thanawongnuwech, R., Suradhat, S., Pariyothorn, N., Tantilertcharoen, R., Damrongwantanapokin, S., Buranathai, C., Chaisingh, A., Songserm, T. and Poovorawan, Y. (2006) Genetic characterization of H5N1 influenza A viruses isolated from zoo tigers in Thailand. *Virology* 344: 480-491.

61. Keawcharoen, J., Oraveerakul, K., Kuiken, T., Fouchier, R.A., Amonsin, A., Payungporn, S., Noppornpanth, S., Wattanodorn, S., Theambooniers, A., Tantilertcharoen, R., Pattanarangsan, R., Arya, N., Ratanakorn, P., Osterhaus, D.M. and Poovorawan, Y. (2004) Avian influenza H5N1 in tigers and leopards. *Emerging Infect. Dis.* 10: 2189-2191.

62. Puthavathana, P., Auewarakul, P., Charoenying, P.C., Sangsiriwut, K., Pooruk, P., Boonnak, K., Khanyok, R., Thawachsupa, P., Kijphati, R. and Sawanpanyalert, P. (2005) Molecular characterization of the complete genome of human influenza H5N1 virus isolates from Thailand. *J. Gen. Virol*. 86: 423-433.

63. Ng, L.F.P., Gupta, S., Chia, J.-M., Liu, J., Hibberd, M.L., Agathe, L.V., Lim, C.-W., Ng, F.-K. and Ren, E.-C. Direct Submission. Submitted (05-JUL-2004) Cell and Medical Biology, Genome Institute of Singapore, 60 Biopolis Street, Genome 02-01, Singapore 138672, Singapore.

64. Le, Q. M., Kiso, M., Someya, K., Sakai, Y. T., Nguyen, T. H., Nguyen, K. H., Pham, N. D., Ngyen, H. H., Yamada, S., Muramoto Y, *et al.* (2005) *Nature* 437: 1108.

65. de Jong, M.D., Simmons, C.P., Thanh, T.T., Hien, V.M., Smith, G.J.D., Chau, T.N.B., Hoang, D.M., Chau, N.V.V., Khanh, T.H., Dong, V.C., Qui, P.T., Cam, B.V., Ha, D.Q., Guan, Y., Peiris, J.S.M., Chinh, N.T., Hien, T.T. and Farrar, J. (2006) Fatal outcome of human influenza A (H5N1) is associated with high viral load and hypercytokinemia. *Nat. Med.* 12: 1203-1207.

66. Cao, B. V., Vo, H. H., Phan, V. T., Le, H. D., Nguyen, T. N., Dong, M. H., Ngo, T. L., Hoang, K. L., Nguyen, T. H., Truong, X. L., Ha, B. K. & Nguyen, K. T. Direct submission. Submitted (12-AUG-2004). Molecular Biology Department, Pasteur Institute, 167 Pasteur, Dist. 3, Ho Chi Minh, Viet Nam.

67. Duong Hong, Q., Nguyen Tien, M., Le Thanh, H., Kumar, P., Lal, S.K., Le Tran, B. & Dinh Duy, K. Direct submission. Submitted (01-DEC-2004). Dinh Duy K., Molecular Microbiology Lab, Institute of Biotechnology (IBT), VAST, 18, Hoang Quoc Viet, Cau Giay, Hanoi, 10000, VIET NAM.

68. Chen, H., Smith, G. J., Li, K. S., Wang, J., Fan, X. H., Rayner, J. M., Vijaykrishna, D., Zhang, J. X., Zhang, L. J., Guo, C. T., *et al*. (2006) *Proc Natl Acad Sci U S A.* 103: 2845-2850.

69. Govorkova, E. A., Rehg, J. E., Krauss, S., Yen, H. L., Guan, Y., Peiris, M., Nguyen, T. D., Hanh, T. H., Puthavathana, P., Long, H. T., *et al*. (2005) *J. Virol*. 79: 2191-2198.

70. De, B. K., Brownlee, G. G., Kendal, A. P. & Shaw, M. W. (1988) *Nucleic Acids Res.* 16: 4181-4182.

71. Rousset, J. A. F. Direct submission. Submitted (12-OCT-2001). Rousset J.A.F., Vipac, AFSSA-Ploufragan, Zoopole des Croix BP53, 22440 Ploufragan, FRANCE.
