# Supplementary material for: Influenza A H5N1 Immigration Is Filtered Out at Some International Borders
Source: PLoS One. 2008 Feb 27;3(2):e1697. doi: 10.1371/journal.pone.0001697 (PMC2244808; doi:10.1371/journal.pone.0001697)
Supplement: Table S2 — Global and a posteriori convergence across distance matrices (CADM) tests for six distance matrices defined by the H5N1 hemagglutinin phylogeny. (0.02 MB DOC) [file pone.0001697.s002.doc]

**Table S2.** Global and *a posteriori* convergence across distance matrices (CADM) tests for six distance matrices defined by the H5N1 hemagglutinin phylogeny.

**Global CADM test**

H_0_*: The six distance matrices are incongruent*

Friedman's Chi-square = 919.93

Kendall's *W* = 0.4067 *P* = 0.0001 Reject *H_0_*

***A posteriori* CADM tests**

H_0_*: This distance matrix is incongruent with all other five*

Symmetricized migration events *P* = 0.3049 Do not reject *H_0_*

Sample size difference *P* = 0.0385 Reject *H_0_*

Geographic distance *P* = 0.0049 Reject *H_0_*

UniFrac distance *P* = 0.0001 Reject *H_0_*

MPD distance *P* = 0.0001 Reject *H_0_*

MNND distance *P* = 0.0001 Reject *H_0_*
