# Supplementary material for: Influenza A H5N1 Immigration Is Filtered Out at Some International Borders
Source: PLoS One. 2008 Feb 27;3(2):e1697. doi: 10.1371/journal.pone.0001697 (PMC2244808; doi:10.1371/journal.pone.0001697)
Supplement: Figure S1 — a) Maximum likelihood radial phylogeny for 481 sequences of H5N1 hemagglutinin (HA) sampled across 28 localities in Eurasia and Africa. Tree is rooted by isolate A/chicken/Scotland/59. b) 50% majority rule consensus tree with bootstrap values for 1000 replicates. (0.16 MB PDF) [file pone.0001697.s003.pdf]

a

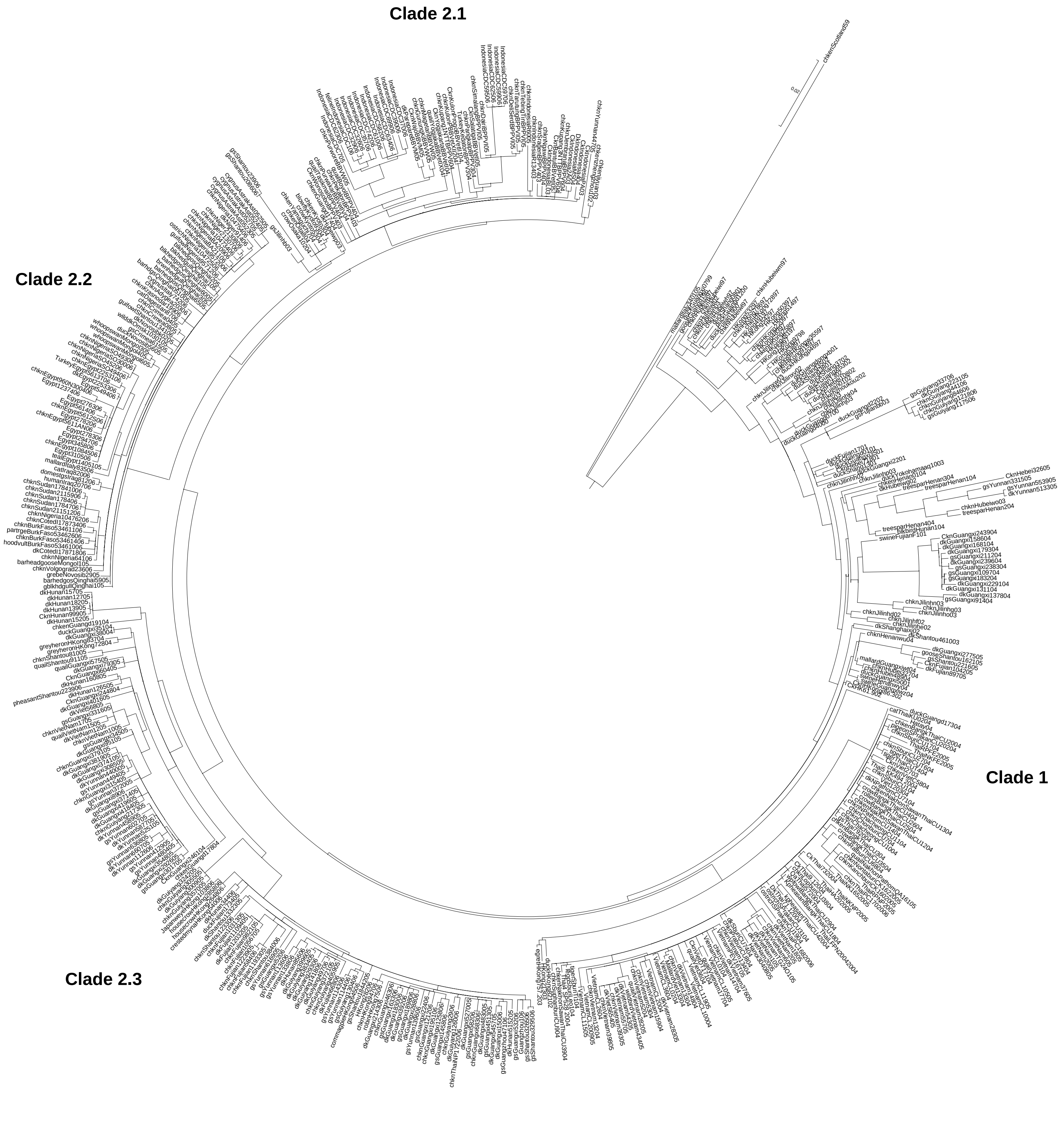

b

```
/------------------------------------------------------------------------------------------------ mallardItaly3401(1)
|
|    /------------------------------------------------------------------------------------------ duckGuangxi0799(3)
|    |
|    |                                                                /----- duckShang3702(4)
|    |                                                                |
|    |                                                                +----- duckGuangxi5302(5)
|    |                                                                |
|    |                                                                +----- duckFujian0102(6)
|    |                                                           /-94--+
|    |                                                           |     +----- CknHebei10802(7)
|    |                                                           |     |
|    |                                                           |     +----- duckFujian1302(8)
|    |                                                           |     |
|    |                                                      /-75-+     \----- chkenzhoukou202(9)
|    |                                                      |    |
|    |                                                      |    +----------- duckShang3801(10)
|    |            /----------------------------99------------------------------+    |
|    |            |                                         |    \----------- duckShang3502(11)
|    |            |                                         |
|    |            |                                         \---------------- gsGuangdongxb01(12)
|    |            |
|    |            +---------------------------------------------------------------------------- chknJilinxv02(13)
|    |            |
|    |            +---------------------------------------------------------------------------- chknJilinhp03(16)
|    |            |
|    |            +---------------------------------------------------------------------------- duckGuangd4000(44)
|    |            |
|    |            +---------------------------------------------------------------------------- duckGuangd0700(45)
|    |            |
|    |            |                                                   /----- chknJilinhk04(46)
|    |            |                                              /-97--+
|    |            |                                              |     \----- chknJilinhj03(47)
|    |            +--------------------------------88---------------------------------+
|    |            |                                              \----------- chknJilinha03(48)
|    |            |
|    |            |                                                   /----- chknGuiyang12180(49)
|    |            |                                              /-73--+
|    |            |                                              |     \----- gsGuiyang117506(50)
|    |            |                                         /-88-+
|    |            |                                         |    \----------- chknGuiyang84606(51)
|    |            |                                   /-85--+
|    |            |                                   |     \---------------- chknGuiyang44106(52)
|    |            |                                   |
|    |            |                              /100-+---------------------- gsGuiyang33706(53)
|    |            |                              |    |
|    |            |                         /-55-+    \---------------------- dkGuiyang223105(54)
|    |            |                         |    |
|    |            +-----------------------97-----------------------+    \-------------------------- gsFujianbb03(55)
|    |            |                         |
|    |            |                         \-------------------------------- duckGuangd2202(56)
|    |            |
|    |            +---------------------------------------------------------------------------- duckFujian1701(57)
|    |            |
|    |            +---------------------------------------------------------------------------- duckShang1301(58)
|    |            |
|    |            |                                                   /----- duckGuangxi3501(59)
|    |            +-----------------------------------73-----------------------------------+
|    |            |                                                   \----- CknHebei71801(60)
|    |            |
|    |            +---------------------------------------------------------------------------- duckGuangd0101(61)
|    |            |
|    |            +---------------------------------------------------------------------------- duckGuangxi2201(62)
|    |            |
|    |            |                                                   /----- gsYunnan553905(63)
|    |            |                                              /-100-+
|    |            |                                              |     \----- dkYunnan513305(64)
|    |            |                                         /100-+
|    |            |                                         |    \----------- gsYunnan331505(65)
|    |            |                                         |
|    |            +------------------------------54------------------------------+---------------- CknHebei32605(66)
```

```
|   |          |                                                 |
|   |          |                                                 |       /----- chknHubeiwo03(67)
|   |          |                                                 \---100----+
|   |          |                                                         \----- treesparHenan204(68)
|   |          |
|   |          +------------------------------------------------------------------------------- treesparHenan104(69)
|   |          |
|   |          +------------------------------------------------------------------------------- treesparHenan304(70)
|   |          |
|   |          |                                                         /----- treesparHenan404(71)
|   |          +-----------------------------------65-----------------------------------+
|   |          |                                                         \----- blkbirdHunan104(72)
|   |          |
|   |          |                                                         /----- gsShantou329506(73)
|   |          |                                                     /-94--+
|   |          |                                                     |   \----- gsShantou326506(74)
|   |          |                                                     |
|   |          |                                        /------70-------+----------- Guangzhou106(75)
|   |          |                                        |            |
|   |          |                                        |            \----------- gsGuangxi53206(76)
|   |          |                                        |
|   |          |                                        +-------------------------- DuckFujian173405(77)
|   |          |                                        |
|   |          |                                        |                /----- dkGuangxi15006(78)
|   |          |                                        |            /-86--+
|   |          |                                        |            |   \----- dkGuangxi545705(79)
|   |          |                                        +------56-------+
|   |          |                                        |            \----------- gsGuangzhou47106(80)
|   |          |                                        |
|   |          |                                        |                /----- chknGuangxi68306(81)
|   |          |                                        +---------99----------+
|   |          |                                        |                \----- gsGuangxi58206(82)
|   |          |                                        |
|   |          |                                        +-------------------------- dkGuangxi527005(83)
|   |          |                                        |
|   |          |                                        +-------------------------- gsGuangxi451305(84)
|   |          |                                        |
|   |          |                                        +-------------------------- dkGuangxi483005(85)
|   |          |                                        |
|   |          |                                        +-------------------------- chknThaiNP172200(86)
|   |          |                                        |
|   |          |                                        |                /----- dkHunan34406(87)
|   |          |                                        +---------80----------+
|   |          |                                        |                \----- crestedmynaHKong(136)
|   |          |                                        |
|   |          |                                        +-------------------------- gsShantou1844205(88)
|   |          |                                        |
|   |          |                                        +-------------------------- dkHunan515205(89)
|   |          |                                        |
|   |          |                                        |                /----- dkGuangxi125806(90)
|   |          |                                        |            /-88--+
|   |          |                                        |            |   \----- chknGuangxi19510(91)
|   |          |                                        +------82-------+
|   |          |                                        |            \----------- gsGuangxi145806(92)
|   |          |                                        |
|   |          |                                        +-------------------------- chknGuangxi12120(93)
|   |          |                                        |
|   |          |                                        +-------------------------- gsGuangxi22406(94)
|   |          |                                        |
|   |          |                                        +-------------------------- gsYunnan139606(95)
|   |          |                                        |
|   |          |                                        |                /----- chknHKong94706(96)
|   |          |                                        |                |
|   |          |                                        +---------94----------+----- HKong28206(97)
|   |          |                                        |                |
|   |          |                                        |                \----- robinHKong7506(98)
|   |          |                                        |
|   |          |                                        |                /----- dkGuiyang126006(99)
|   |          |                                        +---------50----------+
|   |          |                                        |                \----- chknGuiyang2906(100)
|   |          |                                        |
|   |          |                                        |                /----- dkGuangxi183006(101)
|   |          |                                        +---------60----------+
|   |          |                                        |                \----- gsGuangxi163306(103)
|   |          |                                        |
|   |          |                                        +-------------------------- dkGuangxi39206(102)
|   |          |                                        |
```

```
    |   |           |                                   +------------------------- chknGuangxi46306(104)
    |   |           |                                   |
    |   |           |                                   +------------------------- dkGuangxi28806(105)
    |   |           |                               /100-+
    |   |           |                               |   +------------------------- gsGuangxi189806(106)
    |   |           |                               |   |
    |   |           |                               |   +------------------------- dkGuangxi214306(107)
    |   |           |                               |   |
    |   |           |                               |   |               /----- gsYunnan114406(108)
    |   |           |                               |   |           /-96--+
    |   |           |                               |   |           |     \----- gsYunnan114306(109)
    |   |           |                               |   |       /-70-+
    |   |           |                               |   |       |    \---------- gsGuiyang130406(110)
    |   |           |                               |   |   /-69--+
    |   |           |                               |   |   |     \--------------- commagpieHKong64(111)
    |   |           |                               |   |   |
    |   |           |                               |   |   +--------------------- dkFujian66806(112)
    |   |           |                               |   |   |
    |   |           |                               |   |   |           /----- dkGuiyang49706(113)
    |   |           |                               |   |   |       /-65--+
    |   |           |                               |   |   |       |     \----- dkGuiyang141806(115)
    |   |           |                               |   |   |       |
    |   |           |                               |   |   |       |     /----- dkGuiyang383405(114)
    |   |           |                               |   |   |       +-67--+
    |   |           |                               |   +-54-+          |     \----- dkGuiyang399605(117)
    |   |           |                               |   |   +----62----+
    |   |           |                               |   |   |       +---------- chknGuiyang37210(116)
    |   |           |                               |   |   |       |
    |   |           |                               |   |   |       +---------- chknGuiyang40590(118)
    |   |           |                               |   |   |       |
    |   |           |                               |   |   |       \---------- dkGuiyang29306(119)
    |   |           |                               |   |   |
    |   |           |                               |   |   |           /----- gsYunnan133806(120)
    |   |           |                               |   |   |       /-97--+
    |   |           |                               |   |   |       |     \----- gsYunnan113606(121)
    |   |           |                               |   |   \----65----+
    |   |           |                               |   |           \---------- dkHunan85606(122)
    |   |           |                               |   |
    |   |           |                   /---------78----------+    |                /----- chknFujian58406(123)
    |   |           |                   |           |   |           /-93--+
    |   |           |                   |           |   |           |     \----- chknFujian122390(124)
    |   |           |                   |           |   |       /100-+
    |   |           |                   |           |   |       |    \---------- chknFujian119330(125)
    |   |           |                   |           |   |       |
    |   |           |                   |           |   |       |         /----- dkFujian1203205(126)
    |   |           |                   |           |   |   /-58--+          |
    |   |           |                   |           |   |   |     |    /-68--+----- dkFujian1109405(128)
    |   |           |                   |           |   |   |     |    |     |
    |   |           |                   |           |   |   |     |    |     \----- chknFujian103130(129)
    |   |           |                   |           |   |   |     \-98-+
    |   |           |                   |           |   +-60-+          +---------- chknFujian105670(127)
    |   |           |                   |           |   |   |       |
    |   |           |                   |           |   |   |       \---------- chknFujian982105(130)
    |   |           |                   |           |   |   |
    |   |           |                   |           |   |   \--------------------- chknShantou38400(131)
    |   |           |                   |           |   |
    |   |           |                   |           |   +------------------------- gsYunnan616905(132)
/----+   |           |                   |           |   |
|   |   |           |                   |           |   +------------------------- chknShantou12330(133)
|   |   |           |                   |           |   |
|   |   |           |                   |           |   +------------------------- dkShantou1332305(134)
|   |   |           |                   |           |   |
|   |   |           |                   |           |   +------------------------- gsGuangxi5206(135)
|   |   |           |                   |           |   |
|   |   |           |                   |           |   |               /----- housecrowHKong28(137)
|   |   |           |                   |           |   |               |
|   |   |           |                   |           |   \---------99----------+----- housecrowHKong26(138)
|   |   |           |                   |           |                   |
|   |   |           |                   |           |                   \----- JapanweyeHKong10(139)
|   |   |           |                   |           |
|   |   |           |                   |           |                   /----- chknGuiyang35700(140)
|   |   |           |                   |           |                   |
|   |   |           |                   |           |                   +----- dkGuiyang300905(141)
|   |   |           |                   |           \-----------100------------+
|   |   |           |                   |                               +----- chknGuiyang30550(142)
|   |   |           |                   |                               |
|   |   |           |                   |                               \----- dkGuiyang324205(143)
```

```
|   |    |            |                     |
|   |    |            |                     |                                      /----- gsGuangxi301705(144)
|   |    |            |                     |                        /-------91-------+
|   |    |            |                     |                        |             \----- dkGuangxi292605(145)
|   |    |            |                     |                        |
|   |    |            |                     |                        |        /----------- dkYunnan112606(146)
|   |    |            |                     |                        |        |
|   |    |            |                     |                        +----90----+    /----- dkYunnan660705(147)
|   |    |            |                     |                        |        \-98--+
|   |    |            |                     |                        |             \----- gsYunnan636805(148)
|   |    |            |                     |                        |
|   |    |            |                     |                        +---------------------- dkYunnan587705(149)
|   |    |            |                     |                        |
|   |    |            |                     |                        +---------------------- dkYunnan525105(150)
|   |    |            |                     |                        |
|   |    |            |                     |                        +---------------------- gsYunnan412905(151)
|   |    |            |                     |                        |
|   |    |            |                     |                        +---------------------- dkYunnan458905(152)
|   |    |            |                     |                        |
|   |    |            |                     |                        +---------------------- gsYunnan602705(153)
|   |    |            |                     |                        |
|   |    |            |                     |                        +---------------------- chknGuiyang21730(154)
|   |    |            |                     |                        |
|   |    |            |                     |                        +---------------------- gsYunnan480405(155)
|   |    |            |                     |                    /-92-+
|   |    |            |                     |                    |   +---------------------- gsYunnan372005(156)
|   |    |            |                     |                    |   |
|   |    |            |                     |                    |   |             /----- gsYunnan449405(157)
|   |    |            |                     |                    |   |    /----88----+
|   |    |            |                     |                    |   |    |        \----- dkYunnan440005(158)
|   |    |            |                     |                    |   |    |
|   |    |            |                     |                    |   |    |        /----- dkGuangxi381905(165)
|   |    |            |                     |                    |   +-62--+        |
|   |    |            |                     |                    |   |    |   /-71--+----- dkGuangxi374105(166)
|   |    |            |                     |                    |   |    |   |    |
|   |    |            |            /----98----+                      |    |   \-88-+   \----- chknGuangxi37910(168)
|   |    |            |            |        |                    |   |        |
|   |    |            |            |        |                    |   |        \----------- dkGuangxi308505(167)
|   |    |            |            |        |                    |   |
|   |    |            |            |        |                /-95-+   +---------------------- chknGuangxi31540(159)
|   |    |            |            |        |                |   |   |
|   |    |            |            |        |                |   |   +---------------------- dkGuangxi354805(160)
|   |    |            |            |        |                |   |   |
|   |    |            |            |        |                |   |   |             /----- dkGuangxi418405(161)
|   |    |            |            |        |                |   |   |             |
|   |    |            |            |        |                |   |   |             +----- gsGuangxi371405(162)
|   |    |            |            |        |                |   |   \-------84-------+
|   |    |            |            |        |                |   |                 +----- dkGuangxi419605(163)
|   |    |            |            |        |                |   |                 |
|   |    |            |            |        |                |   |                 \----- dkGuangxi8906(164)
|   |    |            |            |        |                |   |
|   |    |            |            |        |           /-83--+   +--------------------------- dkGuangxi95105(169)
|   |    |            |            |        |           |    |   |
|   |    |            |            |        |           |    |   \--------------------------- GsGuangxi34505(170)
|   |    |            |            |        |           |    |
|   |    |            |            |        |           |    |                     /----- quailVietNam1505(171)
|   |    |            |            |        |           |    |                /-96--+
|   |    |            |            |        |           |    |                |    \----- chknVietNam1705(172)
|   |    |            |            |        |           |    |                |
|   |    |            |            |        |           |    |                +----------- dkVietNam1205(173)
|   |    |            |            |        |           |    |            /100-+
|   |    |            |            |        |           |    |            |   +----------- gsGuangxi331605(174)
|   |    |            |            |        |           |    |            |   |
|   |    |            |            |        |       /-67-+    \------98-------+   \----------- dkViet56805(175)
|   |    |            |            |        |       |   |                |
|   |    |            |            |        |       |   |                \--------------- chknVietNam1005(176)
|   |    |            |            |        |       |   |
|   |    |            |            |        |       |   +------------------------------------ dkGuangxi401605(177)
|   |    |            |            |        |       |   |
|   |    |            |            |        |       |   |                        /----- pheasantShantou2(178)
|   |    |            |            |        |  /-84--+   |                      /-68--+
|   |    |            |            |        |  |    |   |                      |    \----- dkHunan160805(180)
|   |    |            |            |        |  |    |   +------------76------------+
|   |    |            |            |        |  |    |   |                      \----------- dkHunan126505(181)
|   |    |            |            |        |  |    |   |
|   |    |            |            |        |  |    |   \------------------------------------ CknGuangxi244804(182)
|   |    |            |            |        |  |    |
```

```
|  |  |      |      |     |  |  \------------------------------------------ CknGuangxi246104(183)
|  |  |      |      |     |  |
|  |  |      |      |     |  |                                /----- greyheronHKong72(179)
|  |  |      |      |     |  +--------------------97---------------------+
|  |  |      |      |     |  |                                \----- greyheronHKong83(190)
|  |  |      |      |     |  |
|  |  |      |      |     +-87-+                                  /----- dkGuangxi79305(184)
|  |  |      |      |     |  |                                |
|  |  |      |      |     |  +--------------------99---------------------+----- quailGuangxi5750(185)
|  |  |      |      |     |  |                                |
|  |  |      |      |     |  |                                \----- CknGuangxi60405(186)
|  |  |      |      |     |  |
|  |  |      |      |     |  +------------------------------------------------ chkenGuangd17804(187)
|  |  |      |      |     |  |
|  |  |      |      |     |  |                                /----- dkGuangxi38004(188)
|  |  |      |      |     |  +--------------------100--------------------+
|  |  |      |      |     |  |                                \----- duckGuangxi35104(189)
|  |  |      |      |     |  |
|  |  |      |      |     |  |                                /----- QuailShantou9110(191)
|  |  |      |      |     |  \--------------------100--------------------+
|  |  |      |      |     |                                   \----- ChknShantou81005(192)
|  |  |      |      |     |
|  |  |      |      |     |                                   /----- dkHunan13905(193)
|  |  |      |      |     |                                   |
|  |  |      |      |     |                              /-63--+----- dkHunan18205(194)
|  |  |      |      |     |                              |     |
|  |  |      |      |     |                              |     \----- CknHunan99905(198)
|  |  |      |      |     |                              |
|  |  |      |      |     |                          /100-+----------- dkHunan12705(195)
|  |  |      |      |     |                          |    |
|  |  |      |      |     |                          |    +----------- dkHunan15205(196)
|  |  |      |      |     \-----------------77------------------+    |
|  |  |      |      |                                |    \----------- dkHunan15705(197)
|  |  |      |      |                                |
|  |  |      |      |                                \---------------- chkenGuangd19104(199)
|  |  |      |      |
|  |  |      |      |                                         /----- chknBurkFaso5346(200)
|  |  |      |      |                                /----75----+
|  |  |      |      |                                |          \----- partrgeBurkFaso5(201)
|  |  |      |      |                                |
|  |  |      |      |                                |          /----- chknBurkFaso5346(202)
|  |  |      |      |                                +----59----+
|  |  |      |      |                                |          \----- chknCotedI178734(250)
|  |  |      |      |                                |
|  |  |      |      |                                +---------------- hoodvultBurkFaso(203)
|  |  |      |      |                                |
|  |  |      |      |                                +---------------- dkCotedI17871806(251)
|  |  |      |      |                   /------62-------+
|  |  |      |      |                   |            +---------------- chknNigeria64106(252)
|  |  |      |      |                   |            |
|  |  |      |      |                   |            +---------------- chknNigeria10476(253)
|  |  |      |      |                   |            |
|  |  |      |      |                   |            |          /----- chknSudan1784706(254)
|  |  |      |      |                   |            |    /-68--+
|  |  |      |      |                   |            |    |     \----- chknSudan178406(255)
|  |  |      |      |                   |            |    |
|  |  |      |      |                   |            |    |     /----- chknSudan2115906(256)
|  |  |      |      |                   |            \-99-+-68--+
|  |  |      |      |                   |                 |     \----- chknSudan1784100(257)
|  |  |      |      |                   |                 |
|  |  |      |      |                   |                 \----------- chknSudan2115120(258)
|  |  |      |      |                   |
|  |  |      |      |                   |                      /----- chknKrasnodar199(204)
|  |  |      |      |                   |                      |
|  |  |      |      |                   |                      +----- chknAdygea20306(205)
|  |  |      |      |                   +------------97------------+
|  |  |      |      |                   |                      +----- CygnusItaly74206(206)
|  |  |      |      |                   |                      |
|  |  |      |      |                   |                      \----- catDagestan8706(207)
|  |  |      |      |                   |
|  |  |      |      |                   +-------------------------------- barhdgsQinghai05(208)
|  |  |      |      |                   |
|  |  |      |      |                   +-------------------------------- barhedgosQinghai(209)
|  |  |      |      |                   |
|  |  |      |      |                   +-------------------------------- barhedgosQinghai(210)
|  |  |      |      |                   |
|  |  |      |      |                   +-------------------------------- brwnhedgullQingh(211)
```

```
|   |    |            |            |                          |
|   |    |            |            |                          +-------------------------------- barhedgosQinghai(212)
|   |    |            |            |                          |
|   |    |            |            |                          +-------------------------------- grebeNovosib2905(213)
|   |    |            |            |                          |
|   |    |            |            |                          +-------------------------------- blkhedgullQingha(214)
|   |    |            |            |                          |
|   |    |            |            |                          +-------------------------------- blkhedgosQinghai(215)
|   |    |            |            |                          |
|   |    |            |            |                          +-------------------------------- gblkhdgullQingha(216)
|   |    |            |            |                          |
|   |    |            |            |                          +-------------------------------- blkhedgosQinghai(217)
|   |    |            |            |                          |
|   |    |            |            |                          |                    /----------- Egypt294706(218)
|   |    |            |            |                          |                    |
|   |    |            |            |                          |                    +----------- chknEgypt1084506(219)
|   |    |            |            |                          |                    |
|   |    |            |            |                          |                    +----------- Egypt278306(220)
|   |    |            |            |                          |                    |
|   |    |            |            |                          |                    +----------- Egypt345806(221)
|   |    |            |            |                          |                    |
|   |    |            |            |                          |                    +----------- Egypt549406(222)
|   |    |            |            |                          |                    |
|   |    |            |            |                          |                    +----------- TurkeyEgypt5613T(223)
|   |    |            |            |                          |                    |
|   |    |            |            |                          |                    +----------- Egypt278206(228)
|   |    |            |            |                          |                    |
|   |    |            |            |                          |                    |     /----- Egypt1237406(229)
|   |    |            |            |                          |               /-94-+-73--+
|   |    |            |            |                          |               |    |     \----- chknEgypt960N300(230)
|   \-100-+           |            |                          |               |    |
|        |            |            |                          |               |    +----------- Egypt310506(231)
|        |            |            |                          |               |    |
|        |            |            |                          |               |    +----------- Egypt561406(232)
|        |            |            |                          |               |    |
|        |            |            |                          |               |    +----------- ChknEgypt5612S06(233)
|        |            |            |                          |               |    |
|        |            |            |                          |               |    +----------- ChknEgypt5611AN0(234)
|        |            |            |                          |               |    |
|        |            |            |                          |         /-94--+    +----------- Egypt276306(235)
|        |            |            |                          |         |     |    |
|        |            |            |                          |         |     |    +----------- chknEgypt2253106(236)
|        |            |            |                    /-100-+         |     |    |
|        |            |            |                    |     |         |     |    \----------- dkEgypt2253306(237)
|        |            |            |                    |     |         |     |
|        |            |            |                    |     |         |     |          /----- chknNigeriaSO494(224)
|        |            |            |                    |     |    /-76-+     |    /-69--+
|        |            |            |                    |     |    |    |     |    |     \----- chknNigeriaSO452(225)
|        |            |            |                    |     |    |    |     |    |
|        |            |            |                    |     |    |    |     \-93-+----------- chknNigeriaSO300(226)
|        |            |            |                    |     |    |    |          |
|        |            |            |                    |     +-93-+    |          \----------- chknNigeriaSO493(227)
|        |            |            |                    |     |    |    |
|        |            |            |                    |     |    |    \---------------------- tealEgypt1405105(238)
|        |            |            |                    |     |    |
|        |            |            |                    |     |    \-------------------------- mallardItaly8350(239)
|        |            |            |                    |     |
|        |            |            |                    |     +-------------------------------- barheadgooseMong(240)
|        |            |            |                    |     |
|        |            |            |                    |     |                          /----- whoopswanMongol4(241)
|        |            |            |                    |     |                    /-90--+
|        |            |            |                    |     |                    |     \----- whoopswanMongol3(242)
|        |            |            |                    |     +---------94---------+
|        |            |       /-83-+                    |     |                    \----------- whoopswanMongol6(243)
|        |            |       |    |                    |     |
|        |            |       |    |                    |     |                          /----- chknNigeriaBA211(244)
|        |            |       |    |                    |     |                    /-62--+
|        |            |       |    |                    |     |                    |     \----- chknNigeriaBA209(245)
|        |            |       |    |                    |     |                    |
|        |            |       |    |                    |     |                    +----------- guifowlNigeria95(266)
|        |            |       |    |                    |     |                    |
|        |            |       |    |                    |     |                    +----------- chknNigeria95720(267)
|        |            |       |    |                    |     |                    |
|        |            |       |    |                    |     |                    +----------- ostrichNigeria10(268)
|        |            |       |    |                    |     |               /-82-+
|        |            |       |    |                    |     |               |    |     /----- chknNiger2130706(269)
|        |            |       |    |                    |     |               |    +-56--+
```

```
|        |            |        |   |                      |    |            |   |    \----- chknNiger2130806(270)
|        |            |        |   |                      |    |            |   |
|        |            |        |   |                      |    |            |   +----------- chknNigeria10473(271)
|        |        /-55-+        |   |                      |    |            |   |
|        |        |   |        |   |                      |    |            |   |    /----- dkNiger91406(272)
|        |        |   |        |   |                      |    |            |   \-53--+
|        |        |   |        |   |                      |    +------73-------+         \----- chknNigeria10475(273)
|        |        |   |        |   |                      |    |            |
|        |        |   |        |   |                      |    |            |        /----- CygnusAstrakAst0(274)
|        |        |   |        |   |                      |    |            |        |
|        |        |   |        |   +------------90------------+    |            +----98----+----- CygnusAstrakAst0(275)
|        |        |   |        |   |                      |    |            |        |
|        |        |   |        |   |                      |    |            |        \----- CygnusAstrakAst0(276)
|        |        |   |        |   |                      |    |            |
|        |        |   |        |   |                      |    |            \---------------- CygnusAstrakAst0(277)
|        |        |   |        |   |                      |    |
|        |        |   |        |   |                      |    |                 /----- domestgsIraq8120(246)
|        |        |   |        |   |                      |    |             /-78--+
|        |        |   |        |   |                      |    |             |     \----- catIraq82006(247)
|        |        |   |        |   |                      |    +---------99---------+
|        |        |   |        |   |                      |    |             \----------- humanIraq20706(248)
|        |        |   |        |   |                      |    |
|        |        |   |        |   |                      |    +-------------------------------- chknVolgograd236(249)
|        |        |   |        |   |                      |    |
|        |        |   |        |   |                      |    |                 /----- chknCrimea0805(259)
|        |        |   |        |   |                      |    +------------91------------+
|        |        |   |        |   |                      |    |                 \----- chknCrimea0405(260)
|        |        |   |        |   |                      |    |
|        |        |   |        |   |                      |    +-------------------------------- GuifowlShantou13(261)
|        |        |   |        |   |                      |    |
|        |        |   |        |   |                      |    +-------------------------------- duckNovosib5605(262)
|        |        |   |        |   |                      |    |
|        |        |   |        |   |                      |    |                 /----- gsCrimea61505(263)
|        |        |   |        |   |                      |    +------------57------------+
|        |        |   |        |   |                      |    |                 \----- wilddkOmsk103010(264)
|        |        |   |        |   |                      |    |
|        |        |   |        |   |                      |    \-------------------------------- dkNovosibk0205(265)
|        |        |   |        |   |                      |
|        |        |   |        |   |                      |                      /----- gsShantou208606(278)
|        |        |   |        |   |                      |                  /-100-+
|        |        |   |        |   |                      |                  |     \----- gsShantou23906(279)
|        |        |   |        |   |                      +------------89------------+
|        |        |   |        |   |                      |                  \----------- gsJilinhb03(280)
|        |        |   |        |   |                      |
|        |        |   |        |   |                      +-------------------------------------- dkHubeiwp03(281)
|        |        |   |        |   |                      |
|        |        |   |        |   |                      +-------------------------------------- chkenGuangd17404(282)
|        |        |   |        |   |                      |
|        |        |   |        |   |                      |                      /----- blowflyKyoto9304(283)
|        |        |   |        |   |                      |                  /-74--+
|        |        |   |        |   |                      |                  |     \----- chkenKyoto304(284)
|        |        |   |        |   |                      |              /-88-+
|        |        |   |        |   |                      |              |   \----------- crowKyoto5304(285)
|        |        |   |        |   |                      |              |
|        |        |   |        |   |                      |              +---------------- chkenYamaguchi70(286)
|        |        |   |        |   |                      \---------100---------+
|        |        |   |        |   |                                     +---------------- crowOsaka10204(287)
|        |        |   |        |   |                                     |
|        |        |   |        |   |                                     \---------------- chkenOita804(288)
|        |        |   |        |   |
|        |        |   |        |   |                                          /----- IndonesiaCDC3290(289)
|        |        |   |        |   |                                      /-75--+
|        |        |   |        |   |                                      |     \----- IndonesiaCDC3260(290)
|        |        |   |        |   |                                  /-88-+
|        |        |   |        |   |                                  |   \----------- felineIndonesiaC(291)
|        |        |   |        |   |                                  |
|        |        |   |        |   |                             /-88--+---------------- IndonesiaCDC3570(292)
|        |        |   |        |   |                             |    |
|        |        |   |        |   |                        /-63-+     \---------------- IndonesiaCDC7420(293)
|        |        |   |        |   |                        |    |
|        |        |   |        |   |                        |    \--------------------- IndonesiaCDC6230(294)
|        |        |   |        |   |                        |
|        |        |   |        |   |                        |                 /----- IndonesiaCDC6340(295)
|        |        |   |        |   |                   /100-+                       |
|        |        |   |        |   |                   |    |                 +----- IndonesiaCDC6100(296)
|        |        |   |        |   |                   |    |             /-66--+
|        |        |   |        |   |                   |    |             |     +----- IndonesiaCDC5820(297)
```

```
|        |        |   |        |   |                          |   |            |    |
|        |        |   |        |   |                          |   \------62-------+    \----- IndonesiaCDC6990(300)
|        |        |   |        |   |                     /-97--+                   |
|        |        |   |        |   |                     |    |                |    /----- IndonesiaCDC3900(298)
|        |        |   |        |   |                     |    |                \-80--+
|        |        |   |        |   |                     |    |                     \----- IndonesiaCDC3700(299)
|        |        |   |        |   |                     |    |
|        |        |   |        |   |                /-98-+    \------------------------------- IndonesiaCDC705(301)
|        |        |   |        |   |                |    |
|        |        |   |        |   |                |    |                          /----- dkPareparеBBVM05(302)
|        |        |   |        |   |                |    |                     /-99--+
|        |        |   |        |   |                |    |                     |    \----- CknWajoBBVM05(303)
|        |        |   |        |   |           /-66--+    \-----------63------------+
|        |        |   |        |   |           |    |                          |    /----- chknGunungKiBBVW(304)
|        |        |   |        |   |           |    |                          \-96--+
|        |        |   |        |   |           |    |                               \----- chknMagetanBBVW0(305)
|        |        |   |        |   |      /-58-+    |
|        |        |   |        |   |      |   |    \----------------------------------------- chknPurworeBBVW0(308)
|        |        |   |        |   |      |   |
|        |        |   |        |   |      |   |                               /----- quailYogjakaBBVe(306)
|        |        |   |        |   |  /-80-+   \-------------------100--------------------+
|        |        |   |        |   |  |   |                                   \----- CknYogjakartaBBV(307)
|        |        |   |        |   |  |   |
|        |        |   |        |   |  |   \----------------------------------------------------- quailBoyolBPPV40(329)
|        |        |   |        |   |  |
|        |        |   |        |   |  |                                 /----------- chknKupang1NTTBP(309)
|        |        |   |        |   |  |                                 |
|        |        |   |        |   |  +---------------------52-----------------------+     /----- chknKulonPBBVetX(310)
|        |        |   |        |   |  |                                 \-100-+
|        |        |   |        |   |  |                                      \----- CknKulonProgoBBV(311)
|        |        |   |        |   |  |
|        |        |   |        |   |  |                                      /----- chknKupang3NTTBP(312)
|        |        |   |        |   |  |                                      |
|        |        |   |        |   |  +------------------------68-------------------------+----- CknBantulBBVetI0(313)
|        |        |   |        |   |  |                                      |
|        |        |   |        |   |  |                                      \----- chknJembranaBPPV(334)
|        |        |   |        |   |  |
|        |        |   |        |   |  |                                      /----- CkIndonesia2A03(314)
|        |        |   |        |   |  +------------------------78-------------------------+
|        |        |   |        |   |  |                                      \----- DkIndonesiaMS04(315)
|        |        |   |        |   |  |
|        |        |   |        |   |  +--------------------------------------------------------- chknPurwakarBBVe(316)
|        |        |   |        |   |  |
|        |        |   |        |   |  |                                      /----- IndonesiaCDC6250(317)
|        |        |   |        |   |  |                                      |
|        |        |   |        |   |  |                                      +----- IndonesiaCDC5990(318)
|        |        |   |        |   |  |                                 /-100-+
|        |        |   |        |   |  |                                 |     +----- IndonesiaCDC5970(319)
|        |        |   |        |   |  |                                 |     |
|        |        |   |        |   |  |                            /-66-+     \----- IndonesiaCDC5950(320)
|        |        |   |        |   |  |                            |    |
|        |        |   |        |   |  |                            |    \----------- chknDairiBPPVI05(321)
|        |        |   |        |   |  |                            |
|        |        |   |        |   +-98--+                          +---------------- chknSimalangBPPV(322)
|        |        |   |        |   |  |                       /-100-+
|        |        |   |        |   |  |                       |    |         /----- chknTarutungBPPV(323)
|        |        |   |        |   |  |                       |    |    /-73--+
|        |        |   |        |   |  |                       |    |    |    \----- chknTebingTinBPP(324)
|        |        |   |        |   |  |                   /-75-+    \-63-+
|        |        |   |        |   |  |                   |   |         \----------- chknDeliSerdBPPV(325)
|        |        |   |        |   |  |                   |   |
|        |        |   |        |   |  +--------------53---------------+    \---------------------- CknSalatigaBBVet(326)
|        |        |   |        |   |  |                   |
|        |        |   |        |   |  |                   \-------------------------- chknPangkalpBPPV(327)
|        |        |   |        |   |  |
|        |        |   |        |   |  +--------------------------------------------------------- turkeyKedatonBPP(328)
|        |        |   |        |   |  |
|        |        |   |        |   |  +--------------------------------------------------------- CkIndonesiaBL03(330)
|        |        |   |        |   |  |
|        |        |   |        |   |  +--------------------------------------------------------- CknWonosoboBPPV4(331)
|        |        |   |        |   |  |
|        |        |   |        |   |  +--------------------------------------------------------- chknNgawiBPPV404(332)
|        |        |   |        |   |  |
|        |        |   |        |   |  +--------------------------------------------------------- chknSragenBPPV40(333)
|        |        |   |        |   |  |
|        |        |   |        |   |  +--------------------------------------------------------- chknPekalonBPPV4(335)
|        |        |   |        |   |  |
```

```
|     |     |  |     |  |  +-------------------------------------------------- quailTasikmalBPP(336)
|     |     |  |     |  |  |
|     |     |  |     |  |  +-------------------------------------------------- CkIndonesia404(337)
|     |     |  |     |  |  |
|     |     |  |     |  |  +-------------------------------------------------- CkIndonesiaPA03(338)
|     |     |  |     |  |  |
|     |     |  |     |  |  +-------------------------------------------------- chknIndonesiaR60(339)
|     |     |  |     |  |  |
|     |     |  |     |  |  \-------------------------------------------------- chknIndonesiaR13(340)
|     |     |  |     |  |
|     |     |  |     |  |                                    /----- chkenzhengzhou10(341)
|     |     |  |     |  |                                /-100-+
|     |     |  |     |  |                                |     \----- chkenjiyuan03(342)
|     |     |  |     |  \-------------------------80--------------------------+
|     |     |  |     |                                   \----------- ChknYunnan44705(343)
|     |     |  |     |
|     |     |  |     |                                     /----- pigeonSPrakanCU2(344)
|     |     |  |     |                  /------------87------------+
|     |     |  |     |                  |                  \----- chknSburiCU1704(345)
|     |     |  |     |                  |
|     |     |  |     |                  |                  /----- leopSburiLeo104(346)
|     |     |  |     |                  +------------54------------+
|     |     |  |  /-57--+                  |                  \----- tigerSburiTi104(347)
|     |     |  |  |  |                  |
|     |     |  |  |  |                  +-------------------------------- Thai4 SP528 2004(348)
|     |     |  |  |  |                  |
|     |     |  |  |  |                  +-------------------------------- Thai5 KK494 2004(349)
|     |     |  |  |  |                  |
|     |     |  |  |  |                  +-------------------------------- chkenBangkThaiCU(350)
|     |     |  |  |  |                  |
|     |     |  |  |  |                  +-------------------------------- CkThai9.12004(351)
|     |     |  |  |  |                  |
|     |     |  |  |  |                  +-------------------------------- crowBangkThaiCU2(352)
|     |     |  |  |  |                  |
|     |     |  |  |  |                  +-------------------------------- chkenNakhonSawan(353)
|     |     |  |  |  |                  |
|     |     |  |  |  |                  +-------------------------------- rollersBangkThai(354)
|     |     |  |  |  |                  |
|     |     |  |  |  |                  +-------------------------------- chkenNakhonSawan(355)
|     |     |  |  |  |                  |
|     |     |  |  |  |                  |   /-------------------------- crowBangkThaiCU3(356)
|     |     |  |  |  |                  |   |
|     |     |  |  |  |                  |   |      /---------------- quailNakhonPatho(431)
|     |     |  |  |  |                  |   |      |
|     |     |  |  |  |                  |   |      |      /----- chknKanchburiCK1(432)
|     |     |  |  |  |                  +-53-+      |      |
|     |     |  |  |  |                  |   |   /-98--+   /-94--+----- ThaiRPNP2005(433)
|     |     |  |  |  |                  |   |   |   |   |   |
|     |     |  |  |  |                  |   |   |   |   |   \----- chknNontaburiCK1(434)
|     |     |  |  |  |                  |   |   |   \-54-+
|     |     |  |  |  |                  |   \-87-+      |   /----- chknThaiPC170200(435)
|     |     |  |  |  |                  |       |      \-99--+
|     |     |  |  |  |                  |       |          \----- ThaiNK1652005(436)
|     |     |  |  |  |                  |       |
|     |     |  |  |  |                  |       \---------------------- chknRatburiCU680(437)
|     |     |  |  |  |                  |
|     |     |  |  |  |                  +-------------------------------- chkenNakhonSawan(357)
|     |     |  |  |  |                  |
|     |     |  |  |  |                  +-------------------------------- chkenBangkThaiCU(358)
|     |     |  |  |  |                  |
|     |     |  |  |  |                  |        /---------------- VietnamPEV16T05(359)
|     |     |  |  |  |                  |        |
|     |     |  |  |  |                  |        |      /----- VietnamCL200905(397)
|     |     |  |  |  |                  |        +---100----+
|     |     |  |  |  |                  |        |      \----- VietnamCL11505(398)
|     |     |  |  |  |                  |        |
|     |     |  |  |  |                  |        |      /----- dkVietnam55705(399)
|     |     |  |  |  |                  |        |      |
|     |     |  |  |  |                  |        |      +----- dkVietS65405(400)
|     |     |  |  |  |                  |        |      |
|     |     |  |  |  |                  |        |      +----- dkVietnam54305(401)
|     |     |  |  |  |                  |        |   /-79--+
|     |  /-59--+  |  |  |                  |        |   |   +----- chknVietnam39805(402)
|     |  |  |  |  |  |                  |        |   |   |
|     |  |  |  |  |  |                  |        |   |   +----- chknVietnam39305(403)
|     |  |  |  |  |  |                  |        +-95-+   |
|     |  |  |  |  |  |                  |        |   |   \----- wildbirdVietnam4(404)
```

```
|       |  |   |  |  |   |                         |           |  |
|       |  |   |  |  |   |                         |           |  \----------- quailVietnam2820(405)
|       |  |   |  |  |   |                         |           |
|       |  |   |  |  |   |                         |           |     /----- VietnamCL11905(406)
|       |  |   |  |  |   |                         |           +----99----+
|       |  |   |  |  |   |                         |           |     \----- VietnamCL10004(407)
|       |  |   |  |  |   |                         |           |
|       |  |   |  |  |   |                         |           |     /----- chknVietnam5304(408)
|       |  |   |  |  |   |                         |           +----93----+
|       |  |   |  |  |   |                         |           |     \----- chknVietnam5204(409)
|       |  |   |  |  |   |                         |           |
|       |  |   |  |  |   |                         |           |     /----- VietnamCL10505(410)
|       |  |   |  |  |   |                         |           +----95----+
|       |  |   |  |  |   |                         |           |     \----- quailVietnam1770(411)
|       |  |   |  |  |   |                         +------57-------+
|       |  |   |  |  |   |                         |           +---------------- VietnamCL0204(412)
|       |  |   |  |  |   |                         |           |
|       |  |   |  |  |   |                         |           +---------------- VietnamCL3604(413)
|       |  |   |  |  |   |                         |           |
|       |  |   |  |  |   |                         |           +---------------- VietnamCL2604(414)
|       |  |   |  |  |   |                         |           |
|       |  |   |  |  |   |                         |           +---------------- VietnamCL0104(415)
|       |  |   |  |  |   |                         |           |
|       |  |   |  |  |   |                         |           +---------------- CknVietDT17104(416)
|       |  |   |  |  |   |                         |           |
|       |  |   |  |  |   |                         |           +---------------- chknVietnam13404(417)
|       |  |   |  |  |   |                         |           |
|       |  |   |  |  |   |                         |           +---------------- chknVietnam13204(418)
|       |  |   |  |  |   |                         |           |
|       |  |   |  |  |   |                         |           +---------------- quailViet3604(419)
|       |  |   |  |  |   |                         |           |
|       |  |   |  |  |   |                         |           +---------------- chknVietnam14704(420)
|       |  |   |  |  |   |                         |           |
|       |  |   |  |  |   |                         |           +---------------- VietnamCL2004(421)
|       |  |   |  |  |   |                     /-96--+              |
|       |  |   |  |  |   |                     |   |           +---------------- dkVietnam28305(422)
|       |  |   |  |  |   |                     |   |           |
|       |  |   |  |  |   |                     |   |           +---------------- chknVietnam15904(423)
|       |  |   |  |  |   |                     |   |           |
|       |  |   |  |  |   |                     |   |           |     /----- chknVietnam13304(424)
|       |  |   |  |  |   |                     |   |           +----72----+
|       |  |   |  |  |   |                     |   |           |     \----- MallardViet13304(425)
|       |  |   |  |  |   |                     |   |           |
|       |  |   |  |  |   |                     |   |           +---------------- dkVietnam14804(426)
|       |  |   |  |  |   |                     |   |           |
|       |  |   |  |  |   |                     |   |           \---------------- dkVietnam37605(427)
|       |  |   |  |  |   |                     |   |
|       |  |   |  |  |   |                     |   +-------------------------------- chknSuphanburiCU(360)
|       |  |   |  |  |   |                     |   |
|       |  |   |  |  |   |                     |   +-------------------------------- chknNPathomCU140(361)
|       |  |   |  |  |   |                     |   |
|       |  |   |  |  |   |                     |   |                 /----- ThaiRPFE2005(362)
|       |  |   |  |  |   |                     |   +------------75------------+
|       |  |   |  |  |   |                     |   |                 \----- ThaiNKFE2005(363)
|       |  |   |  |  |   |                     |   |
|       |  |   |  |  |   |                     |   |                 /----- ostrichSPrakanCU(364)
|       |  |   |  +-66-+   |                     |   +------------77------------+
|       |  |   |  |  |   |                     |   |                 \----- OstrichSPrakanCU(365)
|       |  |   |  |  |   |                     |   |
|       |  |   |  |  |   |                     |   +-------------------------------- chknChacheongCU1(366)
|       |  |   |  |  |   |                     |   |
|       |  |   |  |  |   |                     |   +-------------------------------- chknChacheongCU1(367)
|       |  |   |  |  |   |                     |   |
|       |  |   |  |  |   |                     |   +-------------------------------- chknChonburiCU70(368)
|       |  |   |  |  |   |                     |   |
|       |  |   |  |  |   |                     |   |                 /----- chknVietNam205(369)
|       |  |   |  |  |   |                     |   |             /-53--+
|       |  |   |  |  |   |                     |   |             |   \----- chknVietNam605(370)
|       |  |   |  |  |   |                     |   |             |
|       |  |   |  |  |   |                     |   |          /-62-+    /----- dkVietNam105(371)
|       |  |   |  |  |   |                     |   |          |  |    |
|       |  |   |  |  |   |                     |   |          |  |    +----- dkVietnamTG24O10(372)
|       |  |   |  |  |   |                     |   |          |  \-86--+
|       |  |   |  |  |   |                     |   +------100------+        +----- dkVietNam2005(373)
|       |  |   |  |  |   |                     |   |          |         |
|       |  |   |  |  |   |                     |   |          |         \----- Hanoi3040805(374)
|       |  |   |  |  |   |                     |   |          |
```

```
|     |  |  |  |  |  |                |  |         \---------------- dkVietNam1805(375)
|     |  |  |  |  |  |                |  |
|     |  |  |  |  |  |                |  +-------------------------------- GsThai792004(376)
|     |  |  |  |  |  |                |  |
|     |  |  |  |  |  |                |  +-------------------------------- dkSburiCU7404(377)
|     |  |  |  |  |  |                |  |
|     |  |  |  |  |  +--------------89---------------+   +-------------------------------- chknPraburiCU804(378)
|     |  |  |  |  |  |                |  |
|     |  |  |  |  |  |                |  |                    /----- ThaiLFPN20042004(379)
|     |  |  |  |  |  |                |  |                /-52--+
|     |  |  |  |  |  |                |  |                |     \----- kpheasantThaiCU4(380)
|     |  |  |  |  |  |                |  |                |
|     |  |  |  |  |  |                |  |                +----------- KPheasantBangkTh(381)
|     |  |  |  |  |  |                |  |                |
|     |  |  |  |  |  |                |  +---------55---------+----------- wpfowlBangkThaiC(382)
|     |  |  |  |  |  |                |  |                |
|     |  |  |  |  |  |                |  |                +----------- chknLopburiCU380(383)
|     |  |  |  |  |  |                |  |                |
|     |  |  |  |  |  |                |  |                \----------- QaThai572004(384)
|     |  |  |  |  |  |                |  |
|     |  |  |  |  |  |                |  |                      /----- tigerThaiCUT604(385)
|     |  |  |  |  |  |                |  |                /-95--+
|     |  |  |  |  |  |                |  |                |     \----- tigerThaiCUT404(386)
|     |  |  |  |  |  |                |  +---------52---------+
|     |  |  |  |  |  |                |  |                \----------- chknSburiCU2704(387)
|     |  |  |  |  |  |                |  |
|     |  |  |  |  |  |                |  |                      /----- ThaiNKNP2005(388)
|     |  |  |  |  |  |                |  +------------83------------+
|     |  |  |  |  |  |                |  |                      \----- ThaiHA202005(389)
|     |  |  |  |  |  |                |  |
|     |  |  |  |  |  |                |  +-------------------------------- Viet120304(390)
|     |  |  |  |  |  |                |  |
|     |  |  |  |  |  |                |  +-------------------------------- crowBangkThaiCU4(391)
|     |  |  |  |  |  |                |  |
|     |  |  |  |  |  |                |  +-------------------------------- dkNPathomCU7104(392)
|     |  |  |  |  |  |                |  |
|     |  |  |  |  |  |                |  +-------------------------------- chknSburiCU104(393)
|     |  |  |  |  |  |                |  |
|     |  |  |  |  |  |                |  +-------------------------------- CkThai732004(394)
|     |  |  |  |  |  |                |  |
|     |  |  |  |  |  |                |  +-------------------------------- Hatay04(395)
|     |  |  |  |  |  |                |  |
|     |  |  |  |  |  |                |  +-------------------------------- DkThai71.12004(396)
|     |  |  |  |  |  |                |  |
|     |  |  |  |  |  |                |  +-------------------------------- CknViet2703(428)
|     |  |  |  |  |  |                |  |
|     |  |  |  |  |  |                |  +-------------------------------- chknThaiPC168200(429)
|     |  |  |  |  |  |                |  |
|     |  |  |  |  |  |                |  +-------------------------------- chkenVietC5804(430)
|     |  |  |  |  |  |                |  |
|     |  |  |  |  |  |                |  \-------------------------------- catThaiKU0204(438)
|     \-78-+  |  |  |  |                |
|        |  |  |  |  |                |                      /----- HKong21303(439)
|        |  |  |  |  |                |                /-87--+
|        |  |  |  |  |                |                |     \----- egretHKong757.20(440)
|        |  |  |  |  |                \------------78------------+
|        |  |  |  |  |                                 \----------- duckHKong82102(441)
|        |  |  |  |  |
|        |  |  |  |  \----------------------------------------------------------------- duckGuangd17304(442)
|        |  |  |  |
|        |  |  |  |                                           /----- CknFujian104205(443)
|        |  |  |  |                                       /-91--+
|        |  |  |  |                                       |     \----- dkFujian89705(444)
|        |  |  |  |                                   /-79-+
|        |  |  |  |                                   |    \----------- GsShantou221605(445)
|        |  |  |  |                              /-100-+
|        |  |  |  |                              |     \---------------- GooseShantou1621(446)
|        |  |  |  +-------------------------100-------------------------+
|        |  |  |  |                              \--------------------- dkGuangxi277505(447)
|        |  |  |  |
|        |  |  |  |                                           /----- swineHenanwy04(448)
|        |  |  |  +----------------------------------95----------------------------------+
|        |  |  |  |                                           \----- swineGuangxiwz04(449)
|        |  |  |  |
|        |  |  |  +---------------------------------------------------------------------------- duckGuangxi5001(450)
|        |  |  |  |
|        |  |  |  |                                           /----- chknHubei32704(451)
```

```
|            |    |    |    +----------------------------------87----------------------------------+
|            |    |    |    |                                                         \----- chknHubei48904(452)
|            |    |    |    |
|            |    |    |    +---------------------------------------------------------------------------- chknHenanwu04(453)
|            |    |    |    |
|            |    |    |    +---------------------------------------------------------------------------- mallardGuangxiwt(454)
|            |    |    |    |
|            |    |    |    |                                                         /----- CknHKongi86.302(455)
|            |    |    |    +----------------------------------65----------------------------------+
|            |    |    |    |                                                         \----- CkHK61.902(456)
|            |    |    |    |
|            |    |    |    |                                                         /----- dkShanghaixj02(457)
|            |    |    |    \---------------------------------59----------------------------------+
|            |    |    |                                                              \----- dkShantou461003(458)
|            |    |    |
|            |    |    +-------------------------------------------------------------------------------- chknJilinhh02(459)
|            |    |    |
|            |    |    +-------------------------------------------------------------------------------- dkHubeiwg02(460)
|            |    |    |
|            |    |    +-------------------------------------------------------------------------------- chkenHenan0104(461)
|            |    |    |
|            |    |    +-------------------------------------------------------------------------------- duckYokohamaaq10(462)
|            |    |    |
|            |    |    |                                                              /----- dkGuangxi239604(463)
|            |    |    |                                                         /-83--+
|            |    |    |                                                         |     \----- GsGuangxi238304(464)
|            |    |    |                                                         |
|            |    |    |                                                         +----------- GsGuangxi109704(465)
|            |    |    |                                                         |
|            |    |    |                                                         +----------- dkGuangxi229104(466)
|            |    |    |                                                         |
|            |    |    |                                                         +----------- dkGuangxi179304(467)
|            |    |    |                                                         |
|            |    |    |                                                    /-65-+----------- dkGuangxi168104(468)
|            |    |    |                                                    |    |
|            |    |    |                                                    |    +----------- dkGuangxi158604(469)
|            |    |    |                                                    |    |
|            |    |    |                                                    |    +----------- GsGuangxi211204(470)
|            |    |    |                                               /-97--+    |
|            |    |    |                                               |     |    +----------- GsGuangxi183204(471)
|            |    |    |                                               |     |    |
|            |    |    |                                               |     |    \----------- dkGuangxi131104(472)
|            |    |    |                                          /100-+     |
|            |    |    |                                          |    |     \---------------- CknGuangxi243904(473)
|            |    |    |                                          |    |
|            |    |    |                                          |    |                   /----- dkGuangxi137804(474)
|            |    |    |                                          |    \------100-------+
|            |    |    |                                          |                        \----- GsGuangxi91404(475)
|            |    |    +-------------------------61-------------------------+
|            |    |    |                                          +-------------------------- swineFujianF101(476)
|            |    |    |                                          |
|            |    |    |                                          |             /----------- chknJilinhn03(480)
|            |    |    |                                          |             |
|            |    |    |                                          \------100------+     /----- chknJilinhq03(481)
|            |    |    |                                                        \-92--+
|            |    |    |                                                              \----- chknJilinho03(482)
|            |    |    |
|            |    |    +-------------------------------------------------------------------------------- chknJilinhd02(477)
|            |    |    |
|            |    |    |                                                              /----- chknJilinhf02(478)
|            |    |    \------------------------------------73------------------------------------+
|            |    |                                                                   \----- chknJilinhe02(479)
|            |    |
|            |    \------------------------------------------------------------------------------------ chknJilinxw03(43)
|            |
|            |                                                                        /----- chknHubeiwi97(14)
|            +------------------------------------------67-----------------------------------------+
|            |                                                                        \----- chknHubeiwf02(15)
|            |
|            +---------------------------------------------------------------------------------------- chknHubeiwn03(17)
|            |
|            +---------------------------------------------------------------------------------------- chknHubeiwh97(18)
|            |
|            +---------------------------------------------------------------------------------------- chknHubeiwj97(19)
|            |
|            +---------------------------------------------------------------------------------------- duckGuangd1200(20)
|            |
```

```
|         +--------------------------------------------------------------------- duckFujian1900(21)
|         |
|         +--------------------------------------------------------------------- duckShang0801(22)
|         |
|         +--------------------------------------------------------------------- GooseGuangd196(23)
|         |
|         |                                                     /----- chknHubeiwl97(24)
|         +--------------------------------67--------------------------------+
|         |                                                     \----- chknHubeiwm97(32)
|         |
|         |                                                     /----- HKong54297(25)
|         |                                                /-56--+
|         |                                                |     \----- HKong50397(27)
|         |                                            /-74-+
|         |                                            |    \----------- HKong49197(26)
|         |                                            |
|         |                                       /-78--+---------------- ChknHKong78697(36)
|         |                                       |    |
|         |                                  /-71-+    \---------------- ChknHKong72897(37)
|         |                                  |    |
|         |                                  |    \--------------------- HKong53297(28)
|         |                                  |
|         |                             /-66-+         /---------------- HKong51497(29)
|         |                             |    |         |
|         |                             |    |         |         /----- HKong48397(33)
|         |                             |    \----86----+    /-59--+
|         |                             |              |    |     \----- chkenHKong25897(34)
|         |                             |              \-63-+
|         |                             |                   \----------- ChknHKong22097(35)
|         |                             |
|         \----------------------------87----------------------------+                /----------- HKong9798(30)
|                                       |                   |
|                                       |                   +----------- HKong53897(31)
|                                       |              /-98-+
|                                       |              |    |     /----- ChknHKong91597(38)
|                                       |              |    |     |
|                                       |              |    \-54--+----- DuckHKongp4697(39)
|                                       |              |          |
|                                       \------66-------+          \----- GooseHKongw35597(40)
|                                                      |
|                                                      +---------------- HKong48197(41)
|                                                      |
|                                                      \---------------- HKong15697(42)
|
\----------------------------------------------------------------------------------------------- chkenScotland59(2)
```
