# Supplementary material for: Influenza A H5N1 Immigration Is Filtered Out at Some International Borders
Source: PLoS One. 2008 Feb 27;3(2):e1697. doi: 10.1371/journal.pone.0001697 (PMC2244808; doi:10.1371/journal.pone.0001697)
Supplement: Figure S2 — Migration events defined via DELTRAN parsimony through H5N1 maximum likelihood phylogeny of 481 hemagglutinin sequences sampled across 28 localities in Eurasia and Africa. (0.25 MB PDF) [file pone.0001697.s004.pdf]

	N	Guangxi	Thailand	Indonesia	Vietnam	Hong Kong	Guangdong	Yunnan	Guizhou	Fujian	Egypt	Jilin	Nigeria	Hubei	Hunan	Crimea	Henan	Qinghai	Japan	Shanghai	Sudan	Novosibirsk	Mongolia	Burkina Faso	Niger	Italy	Hebei	Iraq	Cote d'Ivoire	
Guangxi	65	0	1	0	1	1	2	4	1	0	0	1	0	2	1	0	2	0	0	0	0	0	0	0	0	1	0	0	0	17
Thailand	53	0	0	0	4	0	0	0	0	0	0	0	0	0	0	0	0	0	0	0	0	0	0	0	0	0	0	0	0	4
Indonesia	52	0	0	0	0	0	0	0	0	0	0	0	0	0	0	0	0	0	0	0	0	0	0	0	0	0	0	0	0	0
Vietnam	48	1	3	0	0	0	0	0	0	0	0	0	0	0	0	0	0	0	0	0	0	0	0	0	0	0	0	0	0	4
Hong Kong	32	0	0	0	0	0	0	0	0	0	0	0	0	0	0	0	0	0	0	0	0	0	0	0	0	0	0	0	0	0
Guangdong	28	9	1	1	1	6	0	3	2	6	0	2	0	1	3	0	1	1	1	2	0	0	0	0	0	0	1	0	0	41
Yunnan	22	0	0	0	0	1	0	0	3	1	0	0	0	0	1	0	0	0	0	0	0	0	0	0	0	0	0	0	0	6
Guizhou	21	0	0	0	0	0	0	0	0	0	0	0	0	0	0	0	0	0	0	0	0	0	0	0	0	0	0	0	0	0
Fujian	18	0	0	0	0	0	0	0	0	0	0	0	0	0	0	0	1	0	0	0	0	0	0	0	0	0	1	0	0	2
Egypt	17	0	0	0	0	0	0	0	0	0	0	0	1	0	0	0	0	0	0	0	0	0	0	0	0	0	0	0	0	1
Jilin	14	1	0	0	0	0	3	0	0	1	0	0	0	0	0	0	1	0	1	1	0	0	0	0	0	0	0	0	0	8
Nigeria	13	0	0	0	0	0	0	0	0	0	0	0	0	0	0	0	0	0	0	0	1	0	0	1	2	0	0	0	1	5
Hubei	12	0	0	0	0	0	1	0	0	1	0	0	0	0	0	0	0	0	0	1	0	0	0	0	0	0	0	0	0	3
Hunan	12	0	0	0	0	0	1	0	0	0	0	0	0	0	0	0	0	0	0	0	0	0	0	0	0	0	0	0	0	1
Crimea	11	0	0	0	0	0	0	0	0	0	0	0	0	0	0	0	0	0	0	0	0	0	0	0	0	1	0	0	0	1
Henan	10	0	0	0	0	0	0	1	0	0	0	0	0	2	1	0	0	0	0	0	0	0	0	0	0	0	1	0	0	5
Qinghai	9	0	0	0	0	0	0	0	0	0	0	0	1	0	0	2	0	0	0	0	0	2	0	0	0	0	0	0	0	5
Japan	7	0	0	0	0	0	0	0	0	0	0	0	0	0	0	0	0	0	0	0	0	0	0	0	0	0	0	0	0	0
Shanghai	6	1	0	0	0	0	0	0	0	1	0	0	0	0	0	0	0	0	0	0	0	0	0	0	0	0	0	0	0	2
Sudan	5	0	0	0	0	0	0	0	0	0	0	0	0	0	0	0	0	0	0	0	0	0	0	0	0	0	0	0	0	0
Novosibirsk	4	0	0	0	0	0	1	0	0	0	0	0	0	0	0	2	0	0	0	0	0	0	1	0	0	0	0	0	0	4
Mongolia	4	0	0	0	0	0	0	0	0	0	1	0	1	0	0	1	0	0	0	0	0	0	0	0	0	1	0	1	0	5
Burkina Faso	4	0	0	0	0	0	0	0	0	0	0	0	0	0	0	0	0	0	0	0	0	0	0	0	0	0	0	0	1	1
Niger	3	0	0	0	0	0	0	0	0	0	0	0	0	0	0	0	0	0	0	0	0	0	0	0	0	0	0	0	0	0
Italy	3	0	0	0	0	0	0	0	0	0	0	0	0	0	0	0	0	0	0	0	0	0	0	0	0	0	0	0	0	0
Hebei	3	0	0	0	0	0	0	0	0	0	0	0	0	0	0	0	0	0	0	0	0	0	0	0	0	0	0	0	0	0
Iraq	3	0	0	0	0	0	0	0	0	0	0	0	0	0	0	0	0	0	0	0	0	0	0	0	0	0	0	0	0	0
Cote d'Ivoire	2	0	0	0	0	0	0	0	0	0	0	0	0	0	0	0	0	0	0	0	0	0	0	0	0	0	0	0	0	0
	**481**	12	6	1	5	8	8	8	6	10	1	3	3	5	6	5	5	1	2	4	1	2	1	1	2	3	3	1	2	**115**
