# Supplementary figures and images for: Influenza A H5N1 Immigration Is Filtered Out at Some International Borders
Source: PLoS One. 2008 Feb 27;3(2):e1697. doi: 10.1371/journal.pone.0001697 (PMC2244808; doi:10.1371/journal.pone.0001697)

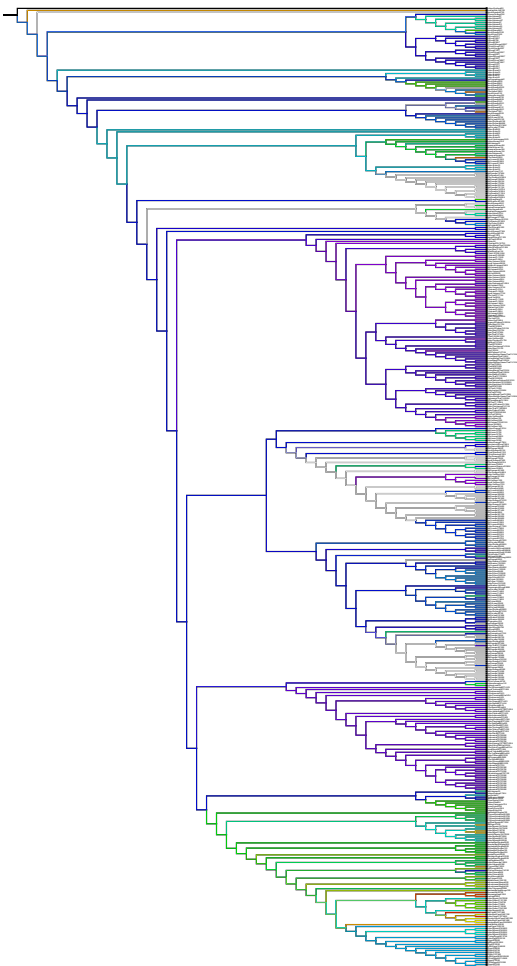

Supplement: Figure S3 — H5N1 migration events assigned on H5N1 hemagglutinin tree by DELTRAN parsimony. Change in color represents migration event. PAUP assignments may represent only one of several possible most-parsimonious traces. Mesquite trace here shows several nodes support multiple localities. Branches that support multiple localities are striped with associated colors. For the best view of isolate names and branch colors, zoom in to at least 800% in Adobe Acrobat. (0.08 MB PDF) [file pone.0001697.s005.pdf]

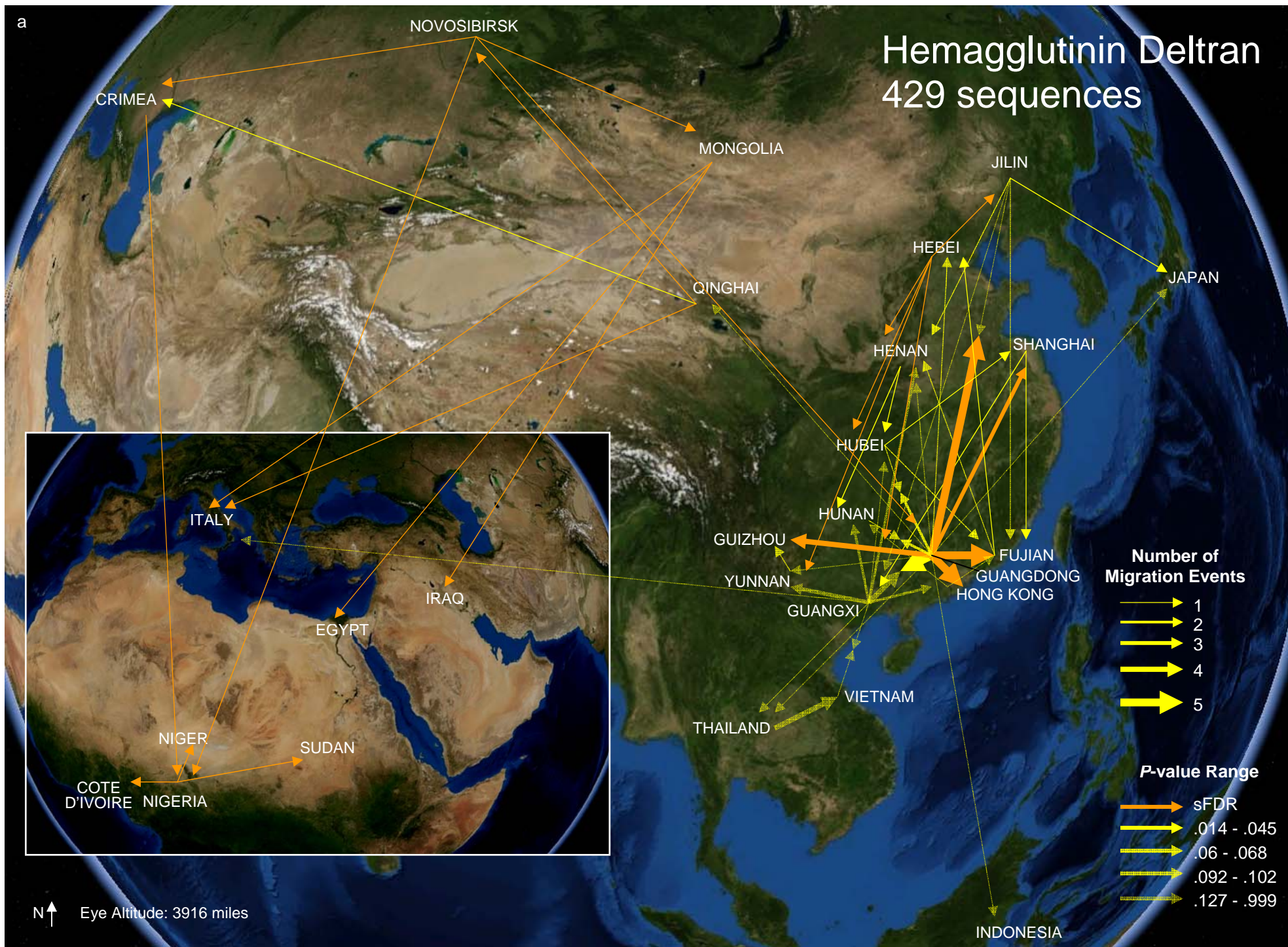

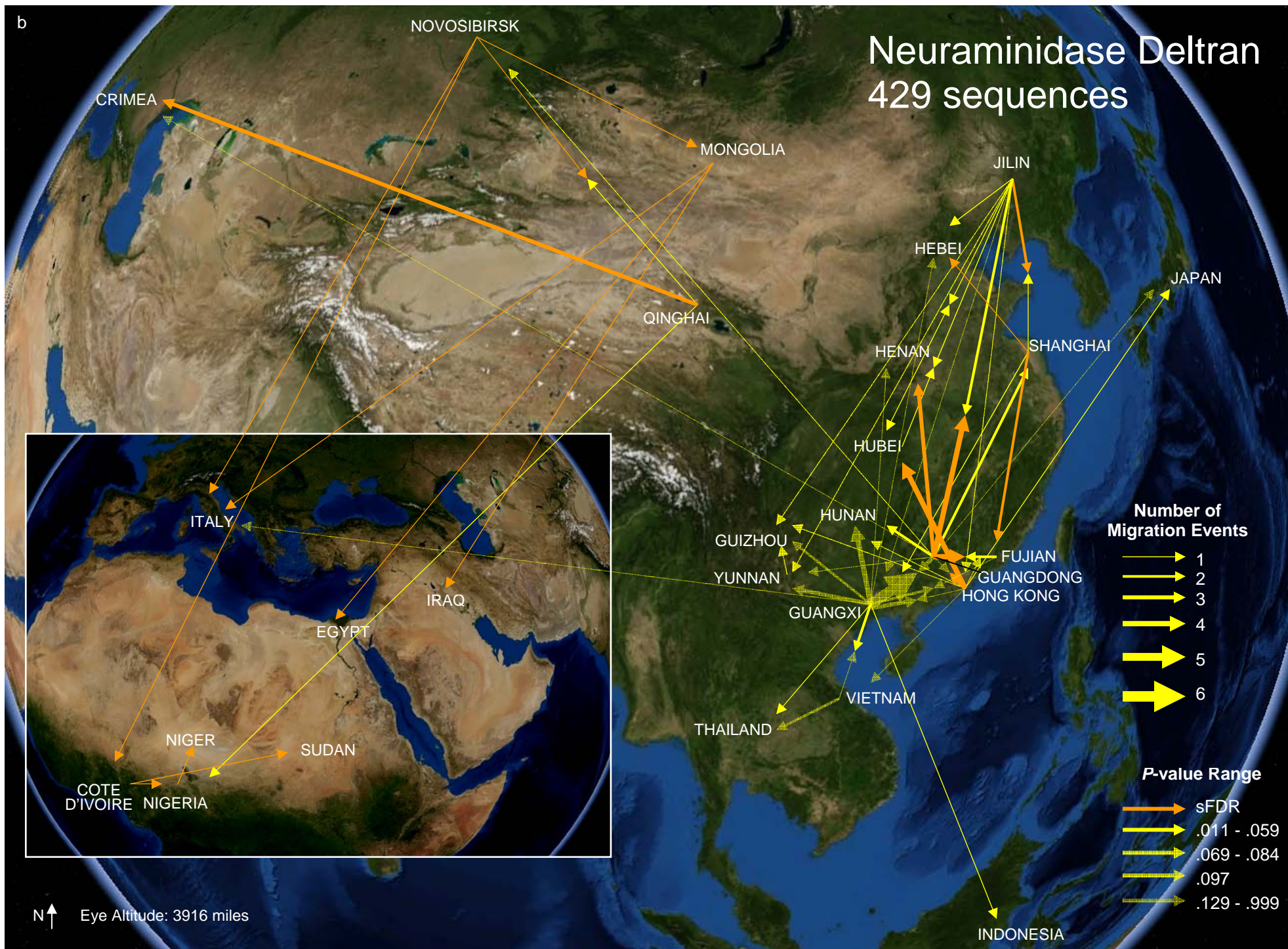

Supplement: Figure S4 — Map of DELTRAN H5N1 migration events inferred through maximum-likelihood phylogeny for 429 hemagglutinin (a) and neuraminidase (b) nucleotide sequences sampled across 28 Eurasian and African localities 1996–2006 (1≤n≤64 isolates per locality). Orange vectors are statistically significant (α = .05) under an upper-tail Monte Carlo test of 10,000 trials and a sparse false discovery rate (sFDR) correction. Non-significant vectors are color-coded by Monte Carlo P value: the brighter the yellow, the greater the support. Quintiles are defined by breaks in ranked P values of more than .01, except within the final quintile. The map is based on satellite photos made available in World Wind 1.4 (http://worldwind.arc.nasa.gov/). (0.54 MB PDF) [file pone.0001697.s006.pdf]

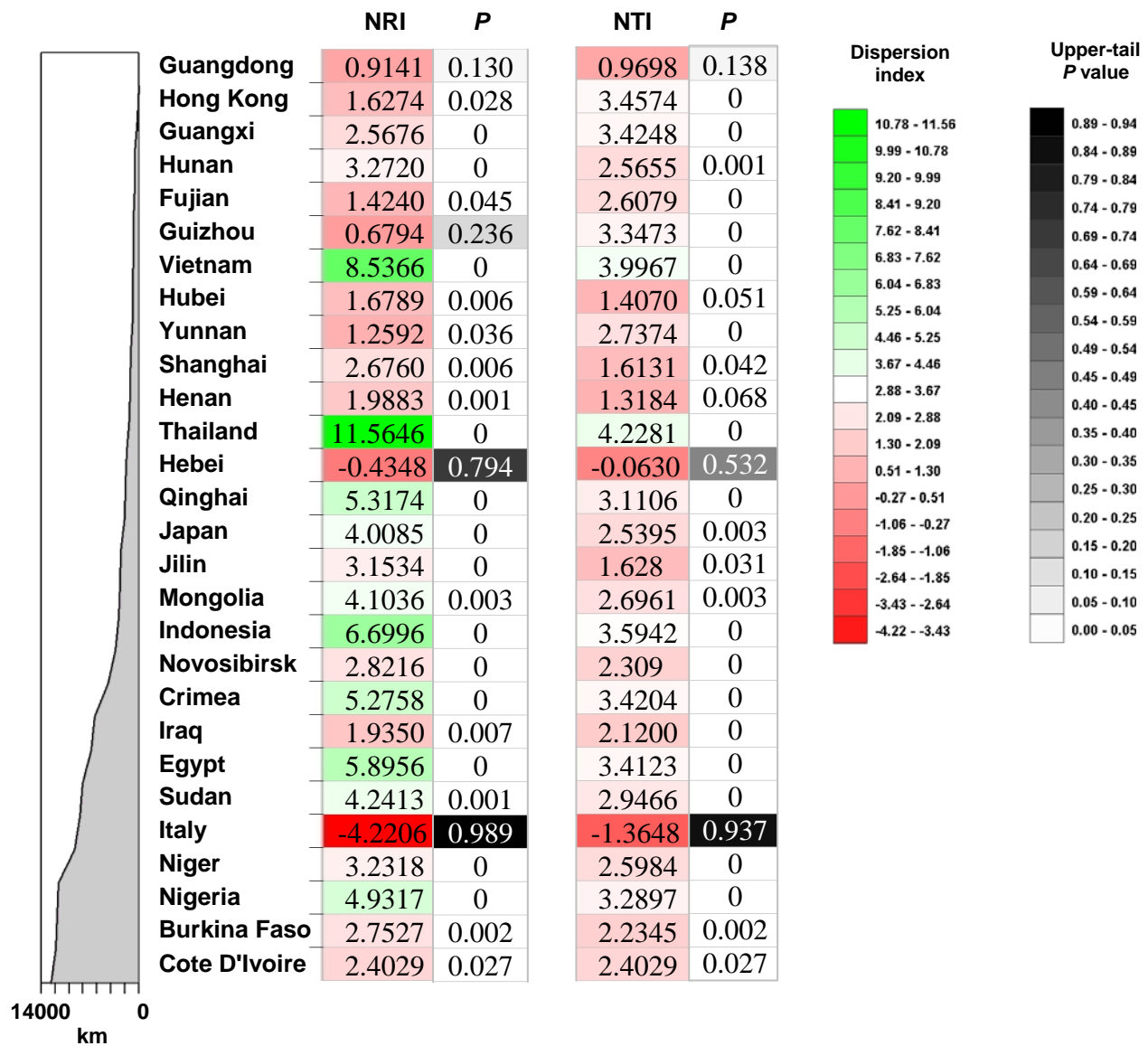

Supplement: Figure S5 — Net relatedness index (NRI), nearest taxon index (NTI), and associated upper-tail P values across 28 localities listed by great circle distance from Guangdong. The original phylogenetic tree of 481 hemagglutinin sequences from which the indices were calculated was constructed by maximum likelihood (Figure S1). (0.11 MB PDF) [file pone.0001697.s007.pdf]
