# Supplementary material for: Influenza A H5N1 Immigration Is Filtered Out at Some International Borders
Source: PLoS One. 2008 Feb 27;3(2):e1697. doi: 10.1371/journal.pone.0001697 (PMC2244808; doi:10.1371/journal.pone.0001697)
Supplement: Figure S6 — A scalar product test for phylogeographic concordance. Degrees difference between eigenvectors for a round robin comparison of migration matrices for the H5N1 hemagglutinin phylogeny of 429 sequences (HA429), the neuraminidase phylogeny of 429 sequences (NA429), and the hemagglutinin phylogeny of 481 sequences (HA481). For HA481 migration events to Burkina Faso were left out as no Burkina Faso neuraminidase sequences were available. For clarity's sake, only the real components of the differences in the eigenvectors are shown. The imaginary components, between eigenvectors 3 and 11 across the three comparisons, suggest pulsing transient diffusion for H5N1 phylogeography. Many of the eigenvectors differ orthogonally (by around 90 degrees) with the smallest average differences for HA429/NA429 and NA429/HA481. (0.16 MB PDF) [file pone.0001697.s008.pdf]

Eigenvector	DELTRAN HA429/ NA429	HA429/ HA481	NA429/ HA481
1	28.17	17.58	31.91
2	147.87	142.98	44.50
3	70.63	60.70	86.18
4	97.03	119.68	101.40
5	96.40	72.60	101.75
6	87.18	72.60	78.83
7	78.98	90.57	89.43
8	97.30	83.61	85.10
9	80.19	93.99	83.91
10	85.15	91.43	66.57
11	80.22	91.43	58.13
12	96.40	58.88	90.00
13	74.62	126.27	90.00
14	100.89	64.76	86.80
15	94.92	101.19	88.44
16	91.03	109.81	99.92
17	59.06	48.63	58.55
18	124.54	126.27	58.55
19	97.42	126.27	90.00
20	91.62	108.79	88.87
21	124.54	126.27	58.55
22	55.46	126.27	121.45
23	55.46	126.27	121.45
24	55.46	134.68	126.92
25	84.36	53.73	90.00
26	124.54	126.27	58.55
27	55.46	126.27	121.45
**Mean**	86.47	97.32	84.34

Degrees difference
141.35 - 147.87
134.84 - 141.35
128.32 - 134.84
121.81 - 128.32
115.30 - 121.81
108.78 - 115.30
102.27 - 108.78
95.75 - 102.27
89.24 - 95.75
82.72 - 89.24
76.21 - 82.72
69.70 - 76.21
63.18 - 69.70
56.67 - 63.18
50.15 - 56.67
43.64 - 50.15
37.12 - 43.64
30.61 - 37.12
24.10 - 30.61
17.58 - 24.10
